# Impact of Baseline SARS-CoV-2 Load in Plasma and Upper Airways on the Incidence of Acute Extrapulmonary Complications of COVID-19: A Multicentric, Prospective, Cohort Study

**DOI:** 10.1093/cid/ciae469

**Published:** 2024-09-13

**Authors:** Tomas O Jensen, Katrina Harper, Shaili Gupta, Sean T Liu, Nila J Dharan, Jason V Baker, Sarah L Pett, Kathryn Shaw-Saliba, Aliasgar Esmail, Minh Q Ho, Eyad Almasri, Robin L Dewar, Jens Lundgren, David M Vock

**Affiliations:** Centre of Excellence for Health, Immunity, and Infections, Rigshospitalet, University of Copenhagen, Copenhagen, Denmark; Division of Biostatistics and Health Data Science, University of Minnesota, Minneapolis, Minnesota, USA; Department of Medicine, Veterans Affairs Connecticut Healthcare System, West Haven, Connecticut, USA; Department of Medicine, Yale School of Medicine, New Haven, Connecticut, USA; Division of Infectious Diseases, Department of Medicine, Icahn School of Medicine at Mount Sinai, New York, New York, USA; Kirby Institute, University of New South Wales Sydney, Sydney, New South Wales, Australia; Division of Infectious Diseases and International Medicine, University of Minnesota, Minneapolis, Minnesota, USA; Division of Infectious Diseases, Hennepin Healthcare, Minneapolis, Minnesota, USA; The Medical Research Council Clinical Trials Unit at UCL, University College London, London, United Kingdom; National Institute of Allergy and Infectious Diseases, National Institutes of Health, Bethesda, Maryland, USA; Division of Pulmonology, Department of Medicine, Centre for Lung Infection and Immunity, University of Cape Town Lung Institute, Cape Town, South Africa; Department of Infectious Diseases, Orlando VA Medical Center, Orlando, Florida, USA; Department of Pulmonology, University of California San Francisco, Fresno, California, USA; Virus Isolation and Serology Laboratory, Frederick National Laboratory for Cancer Research, Frederick, Maryland, USA; Centre of Excellence for Health, Immunity, and Infections, Rigshospitalet, University of Copenhagen, Copenhagen, Denmark; Division of Biostatistics and Health Data Science, University of Minnesota, Minneapolis, Minnesota, USA

**Keywords:** complications, COVID-19, extrapulmonary, nucleocapsid phosphoprotein, SARS-CoV-2 viral load

## Abstract

**Background:**

Extrapulmonary complications (EPCs) are common in patients hospitalized for coronavirus disease 2019 (COVID-19), but data on their clinical consequences and association with viral replication and systemic viral dissemination are lacking.

**Methods:**

Patients hospitalized for COVID-19 and enrolled in the Therapeutics for Inpatients with COVID-19 (TICO) platform trial at 114 international sites between August 2020 and November 2021 were included in a prospective cohort study. We categorized EPCs into 39 event types within 9 categories and estimated their frequency through day 28 and their association with clinical outcomes through day 90. We analyzed the association between baseline viral burden (plasma nucleocapsid antigen [N-Ag] level and upper airway viral load) and EPCs, adjusting for other baseline factors.

**Results:**

A total of 2625 trial participants were included in the study. Their median age was 57 years (interquartile range, 46–68 years), 57.7% were male, and 537 (20.5%) had ≥1 EPC. EPCs were associated with higher day-90 all-cause mortality rate (hazard ratio, 9.6 [95% confidence interval, 7.3–12.7]) after adjustment for other risk factors. The risk of EPCs increased with increasing baseline plasma N-Ag level (hazard ratio, 1.21 per log_10_ ng/L increase [95% confidence interval, 1.09–1.34]), and upper airway viral load (1.12 per log_10_ copies/mL increase [1.04–1.19), after adjustment for comorbid conditions, disease severity, inflammatory markers, and other baseline factors. Trial treatment allocation had no effect on EPC risk.

**Conclusions:**

Systemic viral dissemination as evidenced by high plasma N-Ag level and high respiratory viral burden are associated with development of EPCs in COVID-19, which in turn are associated with higher 90-day mortality rates.

## Teaser

Among 2625 hospitalized patients with coronavirus disease 2019, extrapulmonary complications were common and associated with higher mortality rates. Their frequency increased with plasma nucleocapsid antigen level and upper airway viral load, indicating a likely pathogenetic role of systemic viral dissemination.

## Graphical Abstract

**Graphical Abstract:**
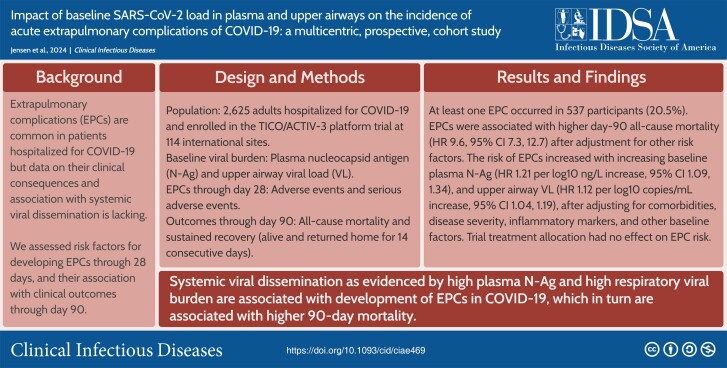
This graphical abstract is also available at Tidbit: https://tidbitapp.io/tidbits/impact-of-baseline-sars-cov-2-load-in-plasma-and-upper-airways-on-the-incidence-of-acute-extrapulmonary-complications-of-covid-19-a-multicentric-prospective-cohort-study-0e7da400-5fb2-41c3-a9f2-2d0a973c8ea4

Extrapulmonary complications (EPCs) including cardiac, gastrointestinal, neurological, renal, and thromboembolic events, are common in coronavirus disease 2019 (COVID-19) and other recently emerged coronavirus infections, such as severe acute respiratory syndrome (SARS) and Middle East respiratory syndrome [1–12]. While a hyperinflammatory state, hypoxia, and general critical illness may all contribute to the development of EPCs, limited data suggest that the frequency of EPCs in COVID-19 is higher than what is expected with critical illness and acute respiratory distress syndrome [3].

Two smaller, single-center studies have suggested that age, COVID-19 severity, and various blood test abnormalities are risk factors for EPCs among patients hospitalized for COVID-19 [13, 14]. Findings of small studies from the United States and China suggested that plasma SARS coronavirus 2 (SARS-CoV-2) RNA levels are correlated with the risk of EPCs [15, 16]. Multiple reports have documented the detection of SARS-CoV-2 in various organ systems [4, 5, 8, 10, 12, 17–19]. In a prospective study of 89 297 hospitalized patients in the United Kingdom, treatment with remdesivir and dexamethasone was associated with decreased frequency of neurological complications [20].

Uncontrolled disseminated SARS-CoV-2 replication may thus play a role in the risk of developing EPCs, and antiviral treatment may modify this risk. Further characterization of the relationship between viral burden and EPCs has the potential to provide valuable insight into the pathogenesis of COVID-19 and to inform the development of future therapies.

Therapeutics for Inpatients with COVID-19 (TICO) under the Accelerating COVID-19 Therapeutic Interventions and Vaccines (ACTIV-3) initiative was an international, multicenter, platform trial designed to evaluate novel treatments for hospitalized COVID-19 patients [21]. None of the first 5 agents in TICO/ACTIV-3 (4 neutralizing monoclonal antibodies [nMAbs] [22–24] and a designed ankyrin repeat protein [DARPin] [25]) demonstrated an effect on the primary outcome of time to sustained recovery, and all trials except one were stopped early for futility. We leveraged data from TICO/ACTIV-3 to describe the frequency and clinical implications of EPCs and evaluate the influence of baseline factors on risk of developing EPCs.

## METHODS

### Study Design and Participants

Participants from trials of the first 5 agents in TICO/ACTIV-3 were eligible for inclusion in this prospective cohort study. Patients hospitalized for COVID-19 were enrolled in TICO/ACTIV-3 between August 2020 and November 2021 if they had laboratory-confirmed COVID-19, were symptomatic for ≤12 days, and had no acute organ failure. Participants were randomized to placebo or a trial agent: bamlanivimab (nMAb; Eli Lilly; August–October 2020), sotrovimab (nMAb; Vir and GlaxoSmithKline; December 2020 to March 2021), amubarvimab-romlusevimab (nMAb; Brii; December 2020 to March 2021), tixagevimab-cilgavimab (nMAb; AstraZeneca; February–September 2021), or ensovibep (DARPin; Molecular Partners; June–November 2021) [22–25]. Remdesivir was provided to all participants unless contraindicated.

Participants were enrolled at 114 sites in Denmark, Greece, Nigeria, Poland, Singapore, Spain, Switzerland, Uganda, the United Kingdom, and the United States. Approvals for the ACTIV-3/TICO trial protocols, including secondary analyses, were obtained from institutional review boards at all sites.

### Procedures

Demographic characteristics and information on comorbid conditions (Supplementary Table 1), concomitant medication, vaccination status, and symptom duration were collected at enrollment (baseline). Disease severity was recorded on a 7-category pulmonary ordinal scale (Supplementary Table 2) at baseline, and blood and respiratory samples were collected for central analysis at the same time.

EPCs were defined as grade 3 or 4 adverse events (AEs), serious AEs (SAEs), and protocol-specified exempt serious events (PSESEs) that occurred through 28 days of follow-up. AEs and SAEs were coded according to the Medical Dictionary for Regulatory Activities (MedDRA). PSESEs were defined in the TICO/ACTIV-3 protocol as SAEs with a simplified reporting pathway since they were expected to occur as part of COVID-19 [21].

In our study, grade 3/4 AEs, SAEs, and PSESEs were recategorized using 2 hierarchical levels: 9 EPC categories, mainly representing organ systems, and specific EPC types within each EPC category. The 9 EPC categories were cardiovascular, gastrointestinal, hematological, hepatic, infectious, miscellaneous, neurological, renal, and venous thromboembolism. The infectious EPC category consisted of all infections other than COVID-19, and the miscellaneous EPC category contained specific EPC types that could not be classified under any of the other 8 categories. The specific EPC types were defined as clinically meaningful acute events, and some represented recategorizations with combination of ≥2 AE/SAE MedDRA preferred terms and/or PSESEs. If a participant experienced the same specific EPC type more than once during the 28-day follow-up period, only the first occurrence was included in the analysis. If a participant experienced multiple specific EPC types within the same EPC category, only 1 occurrence was counted for the purpose of analyzing associations with EPC categories. Two clinical outcomes were used through day-90: all-cause mortality rate and sustained recovery, defined as alive and returned to home for 14 consecutive days.

The plasma nucleocapsid antigen (N-Ag) level was quantified using the Quanterix assay (Quanterix; lower limit of quantification, 3 ng/L). C-reactive protein (CRP) and interleukin 6 (IL-6) were measured using electrochemiluminescence (Meso Scale Discovery), with 10 mg/L and 2 ng/L used as the upper limits of normal for CRP and IL-6, respectively. The presence of the Delta SARS-CoV-2 variant was determined on midturbinate nasal swab samples collected from all participants enrolled after 1 May 2021, using reverse-transcriptase polymerase chain reaction (RT-PCR). Participants enrolled before this date were considered infected with a non-Delta variant. The viral load (VL) was determined on the same sample by quantitative RT-PCR analysis, as described by the US Centers for Disease Control and Prevention [26].

### Statistical Analysis

The frequencies of EPCs during the first 28 days of follow-up after enrollment (baseline) were summarized by EPC categories and specific EPC types within each EPC category. Overlaps between EPC categories were anticipated, and these were visualized with a heat matrix showing overlap size by absolute numbers and percentages of the different EPC categories. Overlap and potential synergy between plasma N-Ag level and baseline pulmonary ordinal scale as risk factors for EPCs were explored by plotting subgroups defined by baseline plasma N-Ag tertile and pulmonary ordinal scale category on a 3-dimensional column chart.

The cumulative incidences of EPCs, of any type and for each of the 9 EPC categories, were assessed using the Aalen-Johansen estimator treating death as competing risk. Results were stratified by baseline plasma N-Ag level using a cutoff of 1000 ng/L, a threshold shown to be associated with worse general outcomes [27]. Sensitivity analyses were performed using a cutoff of 1500 ng/L (the rounded median value; see Table 1) and rounded-cutoff quartiles, as in a more recent analysis of the TICO/ACTIV-3 cohort [28].

**Table 1.** Baseline Participant Characteristics

Characteristic	Participants, No. (%)^a^ (n = 2625)
Age, median (IQR)	57 (46–68)
Female sex	1111 (42.3)
Geographic region	
Africa	131 (5.0)
Asia	42 (1.6)
Europe	393 (15.0)
North America	2059 (78.4)
Race/ethnicity	
Asian	121 (4.6)
Black	628 (23.9)
Hispanic	483 (18.4)
White	1304 (49.7)
Other	89 (3.4)
Comorbid conditions	
Cardiovascular disease	1253 (47.7)
CKD	260 (9.9)
CLD	401 (15.3)
Diabetes	740 (28.2)
Hepatic impairment	40 (1.5)
HIV	42 (1.6)
Immunocompromise	403 (15.4)
Obesity	1376 (52.4)
Any comorbid condition	2176 (82.9)
Vaccination status	
Fully vaccinated^b^	300 (11.4)
Partially vaccinated	226 (8.6)
Not vaccinated	2099 (80.0)
SARS-CoV-2 variant	
Delta	985 (37.5)
Other	1640 (62.5)
Plasma N-Ag	
Positive	2410 (94.8)
Positive, ≥1000 ng/L	1455 (57.2)
Level, median (IQR), ng/L	1445 (234–4731)
Upper airway VL, median (IQR), copies/mL	35 065 (2835–487 271)
Symptom duration, median (IQR), d	8 (6–10)
Pulmonary ordinal scale	
No supplemental oxygen	658 (25.1)
Oxygen <4 L/min	950 (36.2)
Oxygen ≥4 L/min	731 (27.8)
High-flow oxygen or NIV	286 (10.9)
Concomitant medication	
Corticosteroids, ≥10 mg of prednisolone or equivalent	1786 (68.0)
Remdesivir, started before or at enrollment	2431 (92.6)
Heparin, therapeutic dose	112 (4.3)
Trial treatment allocation	
Active	1478 (56.3)
Placebo	1147 (43.7)

Abbreviations: CKD, chronic kidney disease; CLD, chronic lung disease; HIV, human immunodeficiency virus; IQR, interquartile range; N-Ag, nucleocapsid antigen; NIV, noninvasive ventilation; SARS-CoV-2, severe acute respiratory syndrome coronavirus 2; VL, viral load.

^a^Data represent no. (%) of participants unless otherwise specified.

^b^Full primary vaccination course completed; symptoms started ≥14 days after the last dose.

Associations between having ≥1 EPC of any type or any EPC in 1 of the 9 EPC categories during the first 28 days of follow-up and time to clinical outcomes through day 90 were assessed in multivariable analyses. Cox proportional hazards models were constructed to assess the associations with mortality rate through day 90, treating the EPC event as a time-varying covariate. Similar proportional hazards models were constructed to assess the associations with the cause-specific hazard of sustained recovery through day 90, treating the EPC event as a time-varying covariate and censoring at the competing risk of death. Adjustment was made for baseline pulmonary ordinal scale, age, geographic region, chronic kidney disease, immunocompromise, and viral variant since these covariates were associated with worse clinical outcomes in a separate analysis of the same cohort [28].

Two additional Cox proportional hazards models were constructed. The first assessed the association between baseline plasma N-Ag level and the cause-specific hazard of developing ≥1 EPC of any type or ≥1 EPC within each specific EPC category, both during the 28-day follow-up period. The model was adjusted for age, sex, comorbid conditions, COVID-19 vaccination status, viral variant, symptom duration in days, baseline pulmonary ordinal scale, baseline corticosteroid use, treatment allocation in trial, and levels of host inflammatory markers (CRP and IL-6; log_2_). In the second model, plasma N-Ag level was replaced by upper airway VL, and the remaining variables remained identical to the first model. Plasma N-Ag level and upper airway VL were both analyzed as continuous variables after log_10_ transformation. We multiply imputed data sets to address missing host inflammatory markers, using the Markov chain Monte Carlo approach. Parameter estimates were combined using Rubin's combining rules.

Finally, trial treatment allocation, combined and by drug class (nMAb vs placebo or DARPin vs placebo) was analyzed as interaction parameters in the above models. Differences were considered significant at *P* ≤ .05. As these analyses are hypothesis generating, no adjustment for multiple comparisons was performed. All statistical analyses were conducted using SAS software (version 9.4).

## RESULTS

A total of 2625 participants were eligible for inclusion (Table 1). Their median age (interquartile range [IQR]) was 57 (46–68) years, 1514 (57.7%) were male, and 2176 (82.9%) had ≥1 comorbid condition. The median symptom duration (IQR) was 8 (6–10) days, and most participants (n = 2339 [89.1%]) required no oxygen or oxygen supplementation without need for high-flow nasal oxygen or noninvasive ventilation. Remdesivir was prescribed to 2431 participants (92.6%), and 1786 (68.0%) were on a corticosteroid at the time of enrollment. The median plasma N-Ag level (IQR) was 1445 (234–4731) ng/L (Supplementary Figure 1). The number of missing values differed for the baseline variables included in the different analyses (Supplementary Figure 2).

At least 1 EPC was reported during 28-day follow-up for 20.5% of participants (537 of 2625; Table 2). The most common EPC categories were cardiovascular (n = 230 [8.8%]), infectious (n = 191 [7.3%]), miscellaneous (n = 183 [7.0%]), and neurological (n = 137 [5.2%]). Of participants who had ≥1 EPC, 64.8% (348 of 537) experienced the first EPC within 5 days after enrollment. Of the 537 with ≥1 EPC, 328 (61.1%) experienced 1–2 events, and 96 (17.9%) experienced ≥5 (Supplementary Figure 3).

**Table 2.** Extrapulmonary Complications Through 28 Days of Follow-up and Multivariable Analysis of Associations With Clinical Outcomes^a^

EPCs	Participants, No. (%)	Association With Clinical Outcomes at 90 d	
		All-Cause Mortality Rate: HR (95% CI)	Sustained Recovery: RRR (95% CI)
Any EPC	537 (20.5)	9.6 (7.3–12.7)	0.34 (.30–.39)
Cardiovascular EPC, any	230 (8.8)	10.1 (7.7–13.2)	0.23 (.18–.29)
Arrhythmia	50 (1.9)	…	…
Heart failure	12 (0.5)	…	…
Hypotension	196 (7.5)	…	…
Myocardial ischemia	12 (0.5)	…	…
Myocarditis	2 (0.1)	…	…
Pericardial disease	4 (0.2)	…	…
Peripheral artery ischemia	6 (0.2)	…	…
GI EPC, any	64 (2.4)	5.8 (3.8–8.9)	0.46 (.33–.64)
Abdominal pain	10 (0.4)	…	…
Anorexia	26 (1.0)	…	…
Constipation	2 (0.1)	…	…
Diarrhea	14 (0.5)	…	…
Intestinal ischemia	1 (0.04)	…	…
Intestinal obstruction/perforation	2 (0.1)	…	…
Nausea/vomiting	14 (0.5)	…	…
Pancreatitis	1 (0.04)	…	…
Hematological EPC, any	36 (1.4)	6.9 (4.3–10.9)	0.19 (.09–.41)
Anemia	25 (1.0)	…	…
DIC	6 (0.2)	…	…
Pancytopenia	2 (0.1)	…	…
Thrombocytopenia	4 (0.2)	…	…
Hepatic EPC, any	28 (1.1)	12.5 (7.1–22.0)	0.55 (.31–.98)
Infectious EPC	191 (7.3)	6.6 (5.0–8.7)	0.22 (.17–.28)
Miscellaneous EPC, any	183 (7.0)	4.2 (3.2–5.7)	0.44 (.36–.54)
Abnormal bleeding	30 (1.1)	…	…
Fever and/or chills	30 (1.1)	…	…
Hyperglycemia	29 (1.1)	…	…
Weakness and fatigue	101 (3.9)	…	…
Neurological EPC, any	137 (5.2)	4.9 (3.5–6.8)	0.46 (.36–.58)
Cerebral vascular event	12 (0.5)	…	…
Encephalitis	2 (0.1)	…	…
Guillain-Barré syndrome	1 (0.04)	…	…
Headache	20 (0.8)	…	…
Mental/conscious state change	98 (3.7)	…	…
Meningitis	1 (0.04)	…	…
Seizures	4 (0.2)	…	…
Sensory disturbance	2 (0.1)	…	…
Vertigo and dizziness	6 (0.2)	…	…
Renal EPC, any	87 (3.3)	8.5 (6.1–11.8)	0.11 (.06–.20)
Acute kidney injury	74 (2.8)	…	…
Fluid and/or electrolyte disorder	18 (0.7)	…	…
Venous thromboembolism, any	72 (2.7)	4.0 (2.6–6.2)	0.29 (.20–.42)
Deep vein thrombosis	37 (1.4)	…	…
Pulmonary embolism	38 (1.5)	…	…
Superficial vein thrombosis	1 (0.04)	…	…

Abbreviations: CI, confidence interval; DIC, disseminated intravascular coagulation; EPC, extrapulmonary complication; GI, gastrointestinal; HR, hazard ratio; RRR, recovery rate ratio.

^a^With adjustment for baseline pulmonary ordinal scale, age, geographic region, viral variant, chronic kidney disease, and immunocompromise.

Through 90-day follow-up, 261 (9.9%) of 2625 participants died, and 2244 (84.5%) achieved sustained recovery. Having ≥1 EPC through day 28 follow-up was significantly associated with both a higher 90-day all-cause mortality rate (hazard ratio [HR], 9.6 [95% confidence interval (CI): 7.3–12.7]) and a lower rate of sustained recovery through day 90 (recovery rate ratio, 0.34 [.30–.39]), after adjustment for baseline disease severity (as pulmonary ordinal scale), chronic comorbid conditions, and other baseline factors known to be associated with worse clinical outcomes (Table 2). Similar statistically significant associations were found for all the individual EPC categories. The most marked increase in risk of death was seen with hepatic EPCs (HR, 12.5 [95% CI: 7.1–22.0]), cardiovascular EPCs (10.1 [7.7–13.2]), and renal EPCs (8.5 [6.1–11.8]).

**Figure 1.**
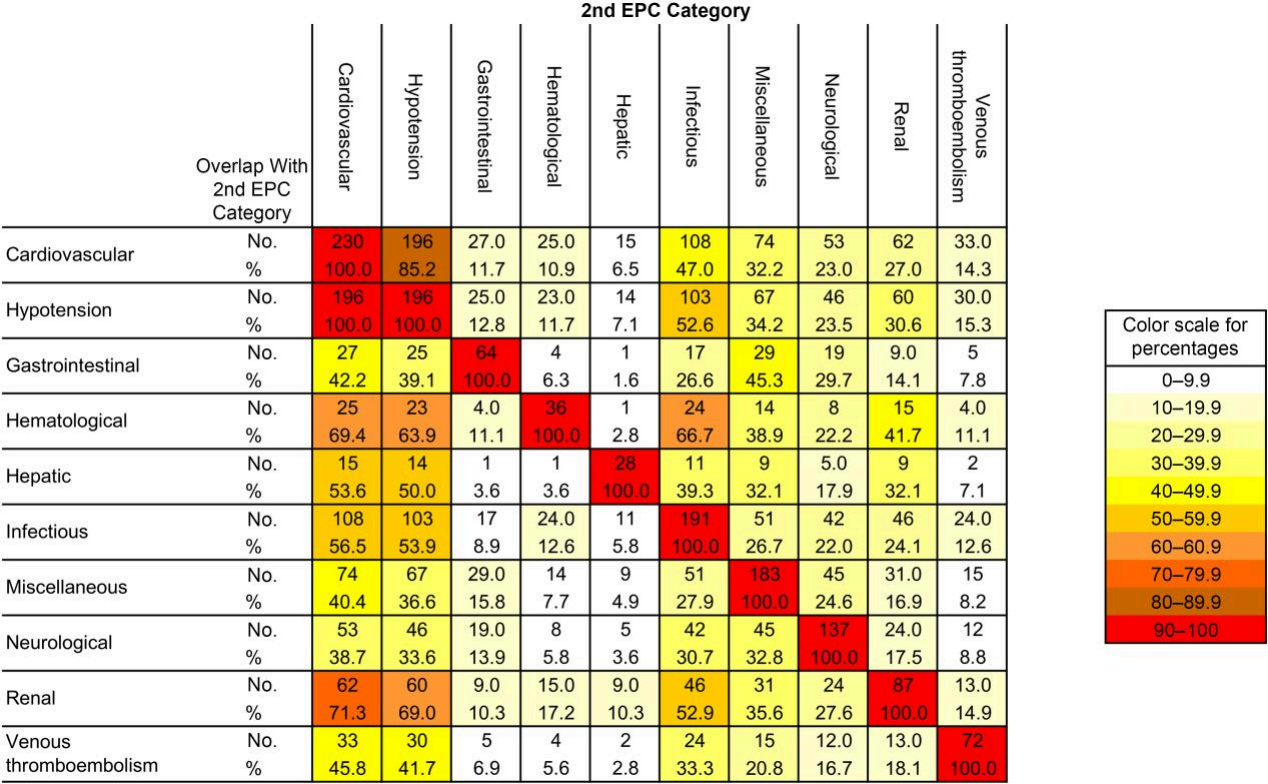
Overlap between 9 categories of extrapulmonary complications (EPCs) through 28 days of follow-up; the specific hypotension EPC type, which is also counted within the cardiovascular EPC category, has been added, as described in Results.

There was substantial overlap among the different EPC categories (Figure 1). A high pattern of overlap, ranging between 38.7% and 71.3%, emerged between noncardiovascular EPC categories and having an EPC in the cardiovascular category. Hypotension was the most common cardiovascular EPC (Table 2). Since hypotension can lead to dysfunction in most other organ systems due to hypoperfusion, we further explored the overlap with hypotension by including this specific EPC type together with the EPC categories in Figure 1. In this analysis, we saw more frequent overlap with hypotension (range 33.6%–69.0% among noncardiovascular EPC categories) compared with any of the noncardiovascular EPC categories.

**Figure 2.**
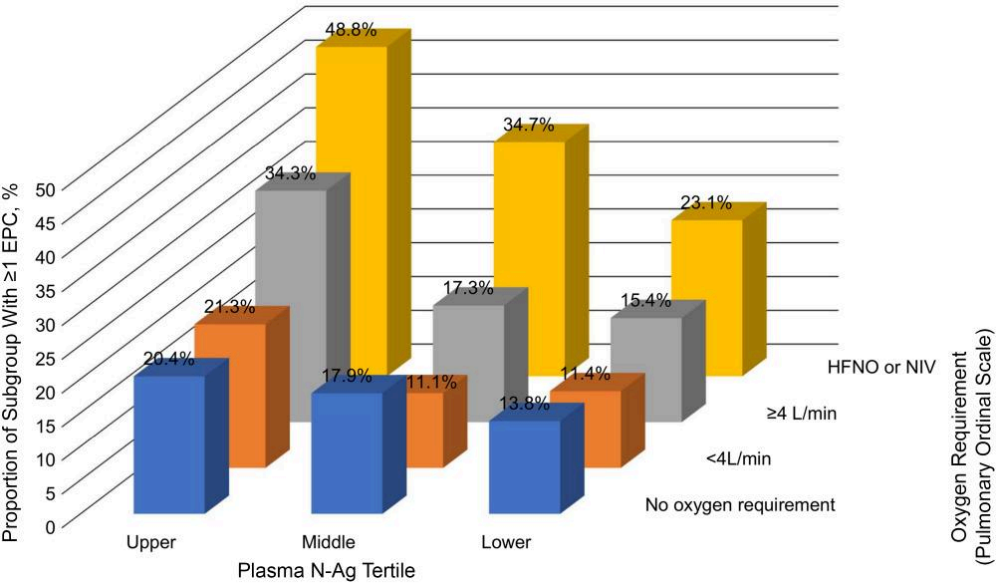
Frequency (as percentage) of any extrapulmonary complication (EPC) through day 28 of follow-up, in subgroups defined by baseline viral burden (plasma nucleocapsid antigen [N-Ag] tertile) and disease severity (oxygen requirement as pulmonary ordinal scale). Abbreviations: HFNO, high-flow nasal oxygen; NIV, noninvasive ventilation.

When dividing the cohort into 12 subgroups based on baseline plasma N-Ag tertile and baseline pulmonary ordinal scale, higher frequencies of EPCs were observed with increases in both plasma N-Ag level and pulmonary ordinal scale (Figure 2). The highest frequency of EPCs (48.8%) was seen in the subgroup with both a plasma N-Ag level in the upper tertile and requirement for high-flow nasal oxygen or noninvasive ventilation.

In the univariate analysis, we found an association between having a plasma level N-Ag ≥1000 ng/L and an EPC of any type (*P* < .001; Figure 3). Similar associations were seen for cardiovascular (*P* < .001), hematological (*P* = .003), hepatic (*P* = .01), infectious (*P* < .01), miscellaneous (*P* < .001), neurological (*P* = .005), and renal (*P* < .001) EPCs (Supplementary Figures 4–12). The cumulative incidence curves demonstrated that most of the excess risk for EPCs among participants with high plasma N-Ag levels was apparent within 2 weeks of enrollment. No substantial differences were observed when using other plasma N-Ag cutoffs (Supplementary Figures 13–32).

**Figure 3.**
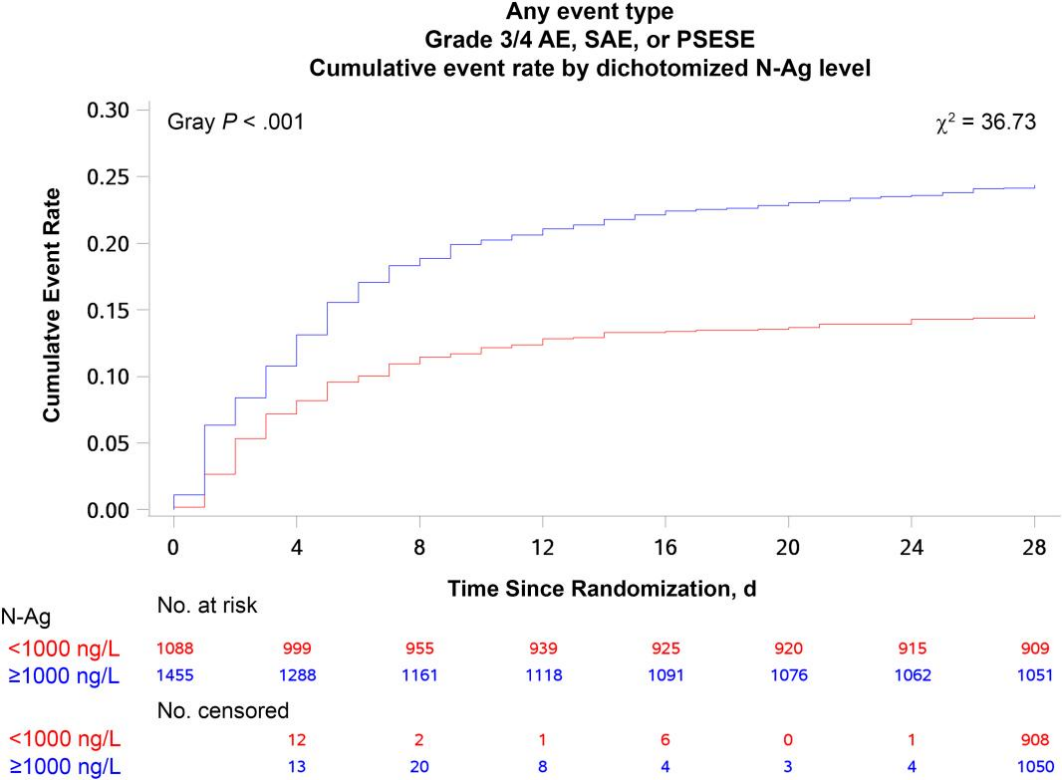
Cumulative incidence of extrapulmonary complications of any type by baseline plasma nucleocapsid antigen (N-Ag) level, using a cutoff of 1000 ng/L. Abbreviations: AE, adverse event; PSESE, protocol-specified exempt serious event; SAE, serious AE.

In the multivariable analysis, a significant association was demonstrated between higher plasma N-Ag level and having ≥1 EPC of any type, with an HR of 1.21 per log_10_ ng/L increase (95% CI: 1.09–1.34) (Table 3). An association was also found between increasing plasma N-Ag and having an EPC in most EPC categories: cardiovascular (HR, 1.63 [95% CI: 1.35–1.96]), hematological (1.79 [1.10–2.92]), infectious (1.42 [1.17–1.71]), miscellaneous (1.29 [1.07–1.56]), and renal (1.41 [1.05–1.90]). The EPC categories in which no association was demonstrated were mostly categories with low numbers of events: gastrointestinal (n = 66), hepatic (n = 28), neurological (n = 137), and venous thromboembolism (n = 84) (Table 2). Supplementary Figure 33 shows the relationship between increasing plasma N-Ag and the HR of having an EPC of any type via a restricted cubic spline curve plot.

**Table 3.** Multivariable Analysis of Associations Between Baseline Factors and Extrapulmonary Complications Through 28 Days of Follow-up^a^

Baseline Factors	HR (95% CI) by EPC Type									
	Any EPC	Cardiovascular	GI	Hematological	Hepatic	Infectious	Miscellaneous	Neurological	Renal	Venous Thromboembolism
Model A^b^ (n = 2513**)**										
Plasma N-Ag, log_10_ ng/L	1.21 (1.09–1.34)	1.63 (1.35–1.96)	1.04 (.78–1.39)	1.79 (1.10–2.92)	1.47 (.89–2.44)	1.42 (1.17–1.71)	1.29 (1.07–1.56)	1.21 (.98–1.50)	1.41 (1.05–1.90)	0.96 (.74–1.25)
Age^c^	1.17 (1.09–1.26)	1.28 (1.14–1.43)	1.05 (.86–1.28)	1.52 (1.13–2.03)	0.84 (.62–1.14)	1.29 (1.14–1.45)	1.14 (1.01–1.28)	1.36 (1.18–1.57)	1.33 (1.10–1.61)	1.10 (.91–1.35)
Comorbid conditions										
CVD	1.10 (.90–1.35)	0.97 (.71–1.32)	1.23 (.68–2.23)	0.58 (.26–1.26)	0.71 (.27–1.83)	1.08 (.77–1.50)	1.11 (.79–1.56)	1.21 (.80–1.82)	1.06 (.62–1.81)	1.08 (.62–1.88)
CKD	1.42 (1.10–1.85)	1.55 (1.03–2.31)	0.72 (.27–1.89)	1.28 (.48–3.43)	1.10 (.31–3.84)	1.10 (.72–1.70)	0.83 (.50–1.38)	1.55 (.95–2.53)	2.77 (1.60–4.79)	0.95 (.41–2.21)
CLD	1.37 (1.10–1.72)	1.30 (.90–1.87)	1.00 (.48–2.05)	1.98 (.87–4.51)	1.75 (.64–4.82)	1.59 (1.10–2.28)	1.42 (.98–2.08)	1.36 (.88–2.10)	1.48 (.86–2.58)	1.64 (.90–2.99)
Diabetes	1.01 (.83–1.24)	1.30 (.95–1.77)	1.26 (.70–2.29)	1.81 (.85–3.88)	1.39 (.53–3.69)	1.08 (.77–1.51)	1.06 (.75–1.50)	0.93 (.62–1.39)	1.31 (.80–2.15)	1.45 (.84–2.50)
Immunocompromise	1.40 (1.12–1.75)	1.38 (.99–1.93)	1.16 (.57–2.34)	3.59 (1.67–7.70)	2.39 (.97–5.89)	1.82 (1.29–2.59)	1.32 (.90–1.95)	0.96 (.59–1.58)	1.60 (.95–2.70)	1.22 (.65–2.28)
Obesity	1.07 (.89–1.29)	1.06 (.79–1.42)	1.21 (.69–2.14)	0.46 (.21–1.00)	0.15 (.05–.46)	0.83 (.61–1.14)	0.96 (.70–1.32)	1.41 (.96–2.07)	1.18 (.73–1.90)	1.09 (.65–1.83)
Pulmonary scale										
No oxygen	Ref	Ref	Ref	Ref	Ref	Ref	Ref	Ref	Ref	Ref
Oxygen <4 L/min	0.80 (.61–1.04)	1.34 (.74–2.40)	0.48 (.23–.99)	1.31 (.39–4.40)	1.86 (.46–7.55)	1.36 (.81–2.31)	0.73 (.46–1.17)	0.75 (.44–1.26)	0.97 (.42–2.23)	1.78 (.69–4.59)
Oxygen ≥4 L/min	1.20 (.92–1.57)	3.65 (2.13–6.27)	0.44 (.20–.99)	1.29 (.37–4.42)	2.98 (.76–11.7)	2.14 (1.28–3.58)	1.19 (.76–1.87)	1.11 (.66–1.88)	2.10 (.98–4.51)	2.31 (.90–5.96)
HFNO/NIV	1.85 (1.35–2.53)	7.39 (4.16–13.2)	1.14 (.48–2.74)	5.88 (1.74–19.9)	1.53 (.26–9.07)	4.12 (2.34–7.26)	1.35 (.78–2.34)	1.19 (.59–2.36)	4.43 (1.94–10.1)	4.15 (1.49–11.5)
Active trial agent	0.88 (.74–1.05)	0.89 (.67–1.17)	0.62 (.37–1.04)	0.98 (.48–2.00)	0.86 (.39–1.89)	0.91 (.68–1.23)	0.83 (.62–1.13)	0.81 (.57–1.16)	1.43 (.89–2.31)	0.89 (.55–1.44)
Inflammatory markers										
CRP (log_2_ mg/L)	1.07 (1.00–1.15)	1.03 (.92–1.14)	1.11 (.91–1.35)	1.19 (.89–1.59)	1.10 (.79–1.53)	1.11 (.99–1.25)	1.03 (.92–1.15)	0.99 (.86–1.14)	1.17 (.97–1.41)	1.16 (.96–1.40)
IL-6 (log_2_ ng/L)	1.11 (1.06–1.17)	1.19 (1.11–1.28)	1.07 (.92–1.25)	1.09 (.90–1.31)	1.14 (.93–1.40)	1.14 (1.06–1.24)	1.12 (1.03–1.22)	1.08 (.97–1.20)	1.19 (1.06–1.34)	1.11 (.97–1.26)
Model B^d^ (n = 2174**)**										
VL (log_10_ copies/mL)	1.12 (1.04–1.19)	1.17 (1.05–1.29)	1.08 (.89–1.31)	1.20 (.92–1.57)	1.07 (.79–1.46)	1.19 (1.07–1.33)	1.05 (.94–1.18)	1.14 (1.00–1.31)	0.96 (.81–1.14)	1.10 (.91–1.33)

Abbreviations: CI, confidence interval; CVD, cardiovascular disease; CKD, chronic kidney disease; CLD, chronic lung disease; CRP, C-reactive protein; EPC, extrapulmonary complication; GI, gastrointestinal; HFNO, high-flow nasal oxygen; HR, hazard ratio; IL-6, interleukin 6; N, number; N-Ag, nucleocapsid antigen; NIV, noninvasive ventilation; Ref, reference; VL, viral load.

^a^Estimates are presented for having 1 EPC of any type and for having 1 EPC of any type within each of the EPC categories. Two models presented, model A using baseline plasma N-Ag as a measure of viral burden and model B using upper airway VL.

^b^Adjustment for all other covariates listed, in addition to sex, vaccination status, viral variant, symptom duration, and corticosteroid use. Covariates that are not shown can be found in Supplementary Table 3.

^c^Age categorized in 10-year bins as age decades.

^d^Adjustment as in model A, but with plasma N-Ag replaced by upper airway VL. Covariates that are not shown can be found in Supplementary Table 4.

Similar associations, although with lower HRs, were observed between upper airway VL and any EPC (HR, 1.12 per log_10_ copies/mL increase [95% CI: 1.04–1.19]), cardiovascular EPCs (1.17 [1.05–1.29]), and infectious EPCs (1.19 [1.07–1.33]) (Table 3 and Supplementary Table 4). EPCs were also associated with older age, some comorbid conditions (chronic kidney disease, chronic lung disease, and immunocompromise), worse pulmonary ordinal scale at baseline, and increasing levels of CRP and IL-6 (Table 3). No association was demonstrated between EPCs and viral variant, symptom duration, or treatment with corticosteroids. Finally, an association was found with not being fully vaccinated (Supplementary Table 3).

No association of EPCs with treatment allocation was observed in the trial (Table 3). The association between plasma N-Ag level and having ≥1 EPC was also not attenuated when treatment allocation was added as an interaction parameter (*P* > .05).

## DISCUSSION

In this cohort of patients hospitalized for COVID-19, we found that ≥1 EPC occurred in 20.5% of participants within 28 days, indicating marked extrapulmonary morbidity and mortality rates; to our knowledge, this is the largest prospective, international cohort to provide such data. We also found that EPCs were associated with poor clinical outcomes, including increased 90-day all-cause mortality rate, and clearly related in a dose-response relationship with markers of viral burden as assessed by baseline plasma N-Ag level and, to a lesser extent, upper airway VL. Importantly, these associations persisted after adjustment for baseline disease severity, level of inflammatory markers, and other baseline clinical factors known to be associated with worse clinical outcomes.

The associations of EPCs with plasma N-Ag levels and upper airway VL suggests that active viral replication and viral dissemination with a direct viral effect in extrapulmonary compartments contribute to the pathogenesis and excess risk of EPCs. This is supported by previous studies detecting SARS-CoV-2 from brain, gastrointestinal tract, heart, kidneys, liver, and spleen [4, 5, 8, 10, 12, 17–19]. Two smaller studies have shown an association between SARS-CoV-2 viremia and EPCs in hospitalized patients [15, 16], and a separate analysis of the ACTIV-3/TICO data set demonstrated that baseline plasma N-Ag level and upper airway VL are associated with 90-day mortality rate [28]. We found stronger associations with EPCs for plasma N-Ag level than for upper airway VL, which may be more susceptible to variation due to sampling technique.

Another important driver of EPC risk was baseline COVID-19 severity, measured by the pulmonary ordinal scale, suggesting tissue hypoxia from respiratory failure as another common pathway for extrapulmonary organ damage. In addition, higher levels of CRP and IL-6 were associated with increased risk of EPCs, indicating that systemic inflammation also plays a role. The association of EPCs with both disease severity, inflammatory markers and baseline plasma N-Ag level persisted in the adjusted analysis, suggesting an additive predictive value from these 3 important risk factors. Patients with high plasma N-Ag level, high oxygen requirement, and high measurements of CRP and/or IL-6 are at particularly high risk of EPCs.

The risk of EPCs was not reduced in patients randomized to active treatment, and the association between plasma N-Ag level and EPC risk was not attenuated in this group neither. This is consistent with the lack of effect on primary outcomes in the trials. However, remdesivir was provided to all participants except a small minority in whom it was contraindicated, which may have affected the analysis.

Being fully vaccinated against COVID-19 was associated with lower risk of EPCs, and treatment with corticosteroids was not associated with reduced risk. However, these results are likely to be substantially affected by confounding by indication and should be interpreted with caution.

Our study has important strengths and limitations. The prospective international enrollment of a large cohort with high retention and follow-up improves generalizability of results to a broader population. An important limitation is that the study population was enrolled before the emergence of the Omicron variant and included a predominantly unvaccinated population with lower prevalence of immunity due to survival of previous infection, emphasizing a need to further characterize EPCs within more contemporary populations.

Despite this, our results do have both current and future relevance. Recent data have shown that the SARS-CoV-2 Omicron variant also has the capacity to persist in various extrapulmonary tissues, and that this is associated with increased risk of post–COVID-19 condition (long COVID) [29]. The risk of future large-scale epidemics continues to increase [30], and other previously emergent novel zoonotic coronaviruses, such as SARS and Middle East respiratory syndrome, have also been demonstrated to be systemic diseases with multiorgan involvement [31–34]. Furthermore, evolving SARS-CoV-2 variants to which there is less natural and vaccine-induced immunity may change the frequency of EPCs in COVID-19.

Our results suggest that EPCs are important causes of disease and death in patients hospitalized with COVID-19 and that their development may be mediated by a systemic SARS-CoV-2 infection that has disseminated beyond pulmonary organ disease. Our data reveal important baseline factors, including plasma N-Ag level, high oxygen requirement, and increased markers of systemic inflammation, that may be used to identify individuals at greater risk of EPCs and associated mortality risk, who would benefit from increased monitoring and perhaps antiviral and/or immunomodulatory treatment. Our data indicate that unchecked viral replication plays a role in the pathogenesis of EPCs in patients hospitalized with COVID-19 and support further studies of antiviral treatments in this population.

## Supplementary Data

Supplementary materials are available at *Clinical Infectious Diseases* online. Consisting of data provided by the authors to benefit the reader, the posted materials are not copyedited and are the sole responsibility of the authors, so questions or comments should be addressed to the corresponding author.

## Supplementary Material

ciae469_Supplementary_Data

## Notes

***Author contributions*.** T. O. J. conceived of the study. All authors participated in its design and in the interpretation of results. K. H. and D. M. V. led the statistical analysis and have directly accessed and verified the underlying data. T. O. J. drafted the manuscript, and all other authors contributed to revisions. All authors read and approved the final manuscript. Authors were not precluded from accessing data in the study, and all authors accept responsibility to submit for publication.

For a STROBE checklist and a full list of the STRIVE ACTIV-3/TICO Study Group, see Supplementary Tables 5 and 6.

***Data availability*.** A deidentified data set is publicly available at https://public-data.ccbr.umn.edu/.

***Financial support.*** This work was supported by the National Institutes of Health (agreement 1OT2HL156812-01 and grant U01-AI136780) and by the National Institute of Allergy and Infectious Diseases and the National Cancer Institute, National Institutes of Health (contract 75N91019D00024 and task orders 75N91020F00014 and 75N91020F00039).
