# Supplementary material for: Impact of Baseline SARS-CoV-2 Load in Plasma and Upper Airways on the Incidence of Acute Extrapulmonary Complications of COVID-19: A Multicentric, Prospective, Cohort Study
Source: Clin Infect Dis. 2024 Sep 13;79(6):1394–403. doi: 10.1093/cid/ciae469 (PMC11650867; doi:10.1093/cid/ciae469)
Supplement: ciae469_Supplementary_Data [file ciae469_supplementary_data.docx]

# Impact of baseline SARS-CoV-2 load in plasma and upper airways on the incidence of acute extrapulmonary complications of COVID-19: a multicentric, prospective, cohort study

## SUPPLEMENTAL MATERIAL

| Figure S1 | Distribution of plasma nucleocapsid antigen measurements (as log10) in the total cohort. | Page 3 |
| --- | --- | --- |
| Figure S2 | Flow diagram showing numbers of participants included and excluded in stages of the study. | Page 4 |
| Figure S3 | Histogram showing the distribution of number of events per participants. | Page 5 |
| Figure S4 | Cumulative incidence of extrapulmonary complications (cardiovascular category) by baseline plasma nucleocapsid antigen using a cutoff of 1,000 ng/L. | Page 6 |
| Figure S5 | Cumulative incidence of extrapulmonary complications (gastrointestinal category) by baseline plasma nucleocapsid antigen using a cutoff of 1,000 ng/L. | Page 7 |
| Figure S6 | Cumulative incidence of extrapulmonary complications (hematological category) by baseline plasma nucleocapsid antigen using a cutoff of 1,000 ng/L. | Page 8 |
| Figure S7 | Cumulative incidence of extrapulmonary complications (hepatic category) by baseline plasma nucleocapsid antigen using a cutoff of 1,000 ng/L. | Page 9 |
| Figure S8 | Cumulative incidence of extrapulmonary complications (infectious category) by baseline plasma nucleocapsid antigen using a cutoff of 1,000 ng/L. | Page 10 |
| Figure S9 | Cumulative incidence of extrapulmonary complications (miscellaneous category) by baseline plasma nucleocapsid antigen using a cutoff of 1,000 ng/L. | Page 11 |
| Figure S10 | Cumulative incidence of extrapulmonary complications (neurological category) by baseline plasma nucleocapsid antigen using a cutoff of 1,000 ng/L. | Page 12 |
| Figure S11 | Cumulative incidence of extrapulmonary complications (renal category) by baseline plasma nucleocapsid antigen using a cutoff of 1,000 ng/L. | Page 13 |
| Figure S12 | Cumulative incidence of extrapulmonary complications (venous thromboembolism category) by baseline plasma nucleocapsid antigen using a cutoff of 1,000 ng/L. | Page 14 |
| Figure S13 | Cumulative incidence of extrapulmonary complications (any event) by baseline plasma nucleocapsid antigen using a cutoff of 1,500 ng/L. | Page 15 |
| Figure S14 | Cumulative incidence of extrapulmonary complications (cardiovascular category) by baseline plasma nucleocapsid antigen using a cutoff of 1,500 ng/L. | Page 16 |
| Figure S15 | Cumulative incidence of extrapulmonary complications (gastrointestinal category) by baseline plasma nucleocapsid antigen using a cutoff of 1,500 ng/L. | Page 17 |
| Figure S16 | Cumulative incidence of extrapulmonary complications (hematological category) by baseline plasma nucleocapsid antigen using a cutoff of 1,500 ng/L. | Page 18 |
| Figure S17 | Cumulative incidence of extrapulmonary complications (hepatic category) by baseline plasma nucleocapsid antigen using a cutoff of 1,500 ng/L. | Page 19 |
| Figure S18 | Cumulative incidence of extrapulmonary complications (infectious category) by baseline plasma nucleocapsid antigen using a cutoff of 1,500 ng/L. | Page 20 |
| Figure S19 | Cumulative incidence of extrapulmonary complications (miscellaneous category) by baseline plasma nucleocapsid antigen using a cutoff of 1,500 ng/L. | Page 21 |
| Figure S20 | Cumulative incidence of extrapulmonary complications (neurological category) by baseline plasma nucleocapsid antigen using a cutoff of 1,500 ng/L. | Page 22 |
| Figure S21 | Cumulative incidence of extrapulmonary complications (renal category) by baseline plasma nucleocapsid antigen using a cutoff of 1,500 ng/L. | Page 23 |
| Figure S22 | Cumulative incidence of extrapulmonary complications (venous thromboembolism category) by baseline plasma nucleocapsid antigen using a cutoff of 1,500 ng/L. | Page 24 |
| Figure S23 | Cumulative incidence of extrapulmonary complications (any event) by baseline plasma nucleocapsid antigen using rounded quartile cutoffs with breakpoints at 200, 1,500, and 4,500 ng/L. | Page 25 |
| Figure S24 | Cumulative incidence of extrapulmonary complications (cardiovascular category) by baseline plasma nucleocapsid antigen using a cutoff of 1,500 ng/L. | Page 26 |
| Figure S25 | Cumulative incidence of extrapulmonary complications (gastrointestinal category) by baseline plasma nucleocapsid antigen using rounded quartile cutoffs with breakpoints at 200, 1,500, and 4,500 ng/L. | Page 27 |
| Figure S26 | Cumulative incidence of extrapulmonary complications (hematological category) by baseline plasma nucleocapsid antigen using rounded quartile cutoffs with breakpoints at 200, 1,500, and 4,500 ng/L. | Page 28 |
| Figure S27 | Cumulative incidence of extrapulmonary complications (hepatic category) by baseline plasma nucleocapsid antigen using rounded quartile cutoffs with breakpoints at 200, 1,500, and 4,500 ng/L. | Page 29 |
| Figure S28 | Cumulative incidence of extrapulmonary complications (infectious category) by baseline plasma nucleocapsid antigen using rounded quartile cutoffs with breakpoints at 200, 1,500, and 4,500 ng/L. | Page 30 |
| Figure S29 | Cumulative incidence of extrapulmonary complications (miscellaneous category) by baseline plasma nucleocapsid antigen using rounded quartile cutoffs with breakpoints at 200, 1,500, and 4,500 ng/L. | Page 31 |
| Figure S30 | Cumulative incidence of extrapulmonary complications (neurological category) by baseline plasma nucleocapsid antigen using rounded quartile cutoffs with breakpoints at 200, 1,500, and 4,500 ng/L. | Page 32 |
| Figure S31 | Cumulative incidence of extrapulmonary complications (renal category) by baseline plasma nucleocapsid antigen using rounded quartile cutoffs with breakpoints at 200, 1,500, and 4,500 ng/L. | Page 33 |
| Figure S32 | Cumulative incidence of extrapulmonary complications (venous thromboembolism category) by baseline plasma nucleocapsid antigen using rounded quartile cutoffs with breakpoints at 200, 1,500, and 4,500 ng/L. | Page 34 |
| Figure S33 | Relationship between increasing plasma N-Ag and the HR of having at least one EPC of any type. | Page 35 |
| Table S1 | Definitions of baseline comorbidities. | Page 36 |
| Table S2 | The 7-category pulmonary ordinal outcome scale. | Page 37 |
| Table S3 | Multivariable analysis of associations between baseline plasma nucleocapsid antigen, other baseline factors, and extrapulmonary complications in total and by event category. | Page 38 |
| Table S4 | Multivariable analysis of associations between baseline upper airway viral load, other baseline factors, and extrapulmonary complications in total and by event category. | Page 39 |
| Table S5 | STROBE checklist | Page 40-41 |
| Table S6 | List of all study group members. | Page 42-73 |

**Figure S1. Distribution of plasma nucleocapsid antigen measurements (as log_10_) in the total cohort.**

**
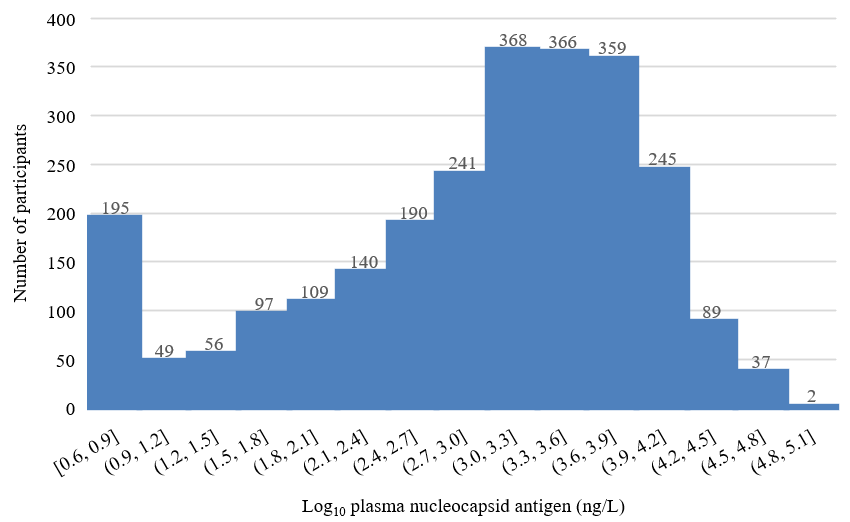
**

**Figure S2. Flow diagram showing numbers of participants included and excluded in stages of the study.**

**
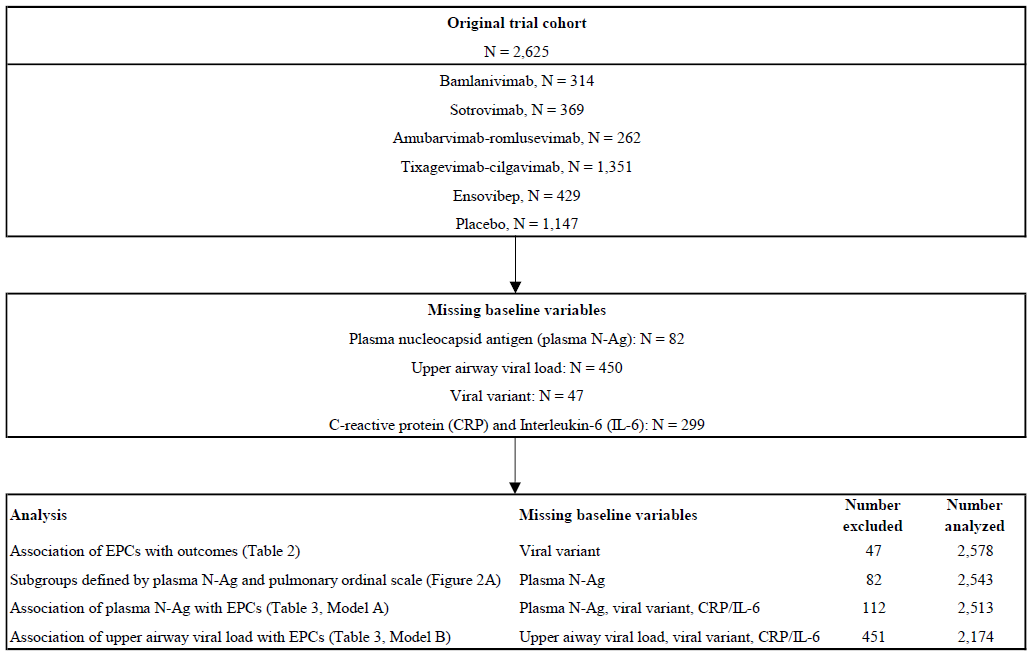
**

**Figure S3. Histogram showing the distribution of number of events per participants.**

**Figure S4. Cumulative incidence of extrapulmonary complications (cardiovascular category) by baseline plasma nucleocapsid antigen using a cutoff of 1,000 ng/L.**

**
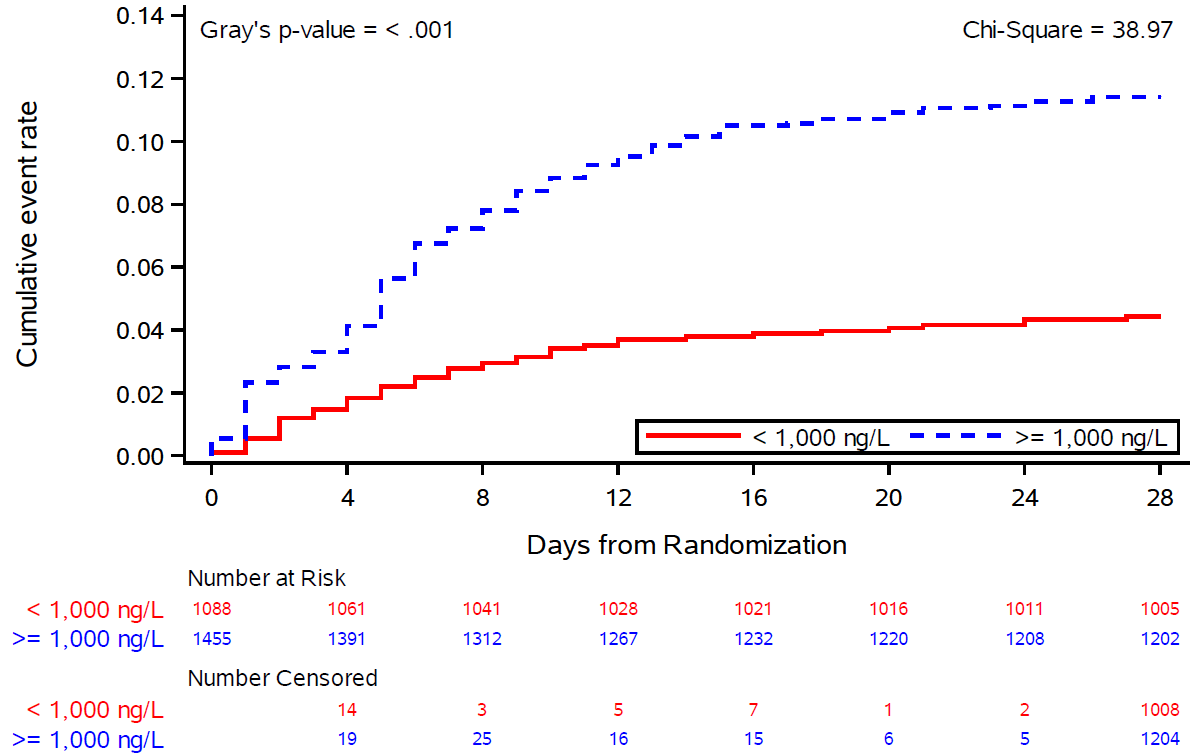
**

**Figure S5. Cumulative incidence of extrapulmonary complications (gastrointestinal category) by baseline plasma nucleocapsid antigen using a cutoff of 1,000 ng/L.**

**
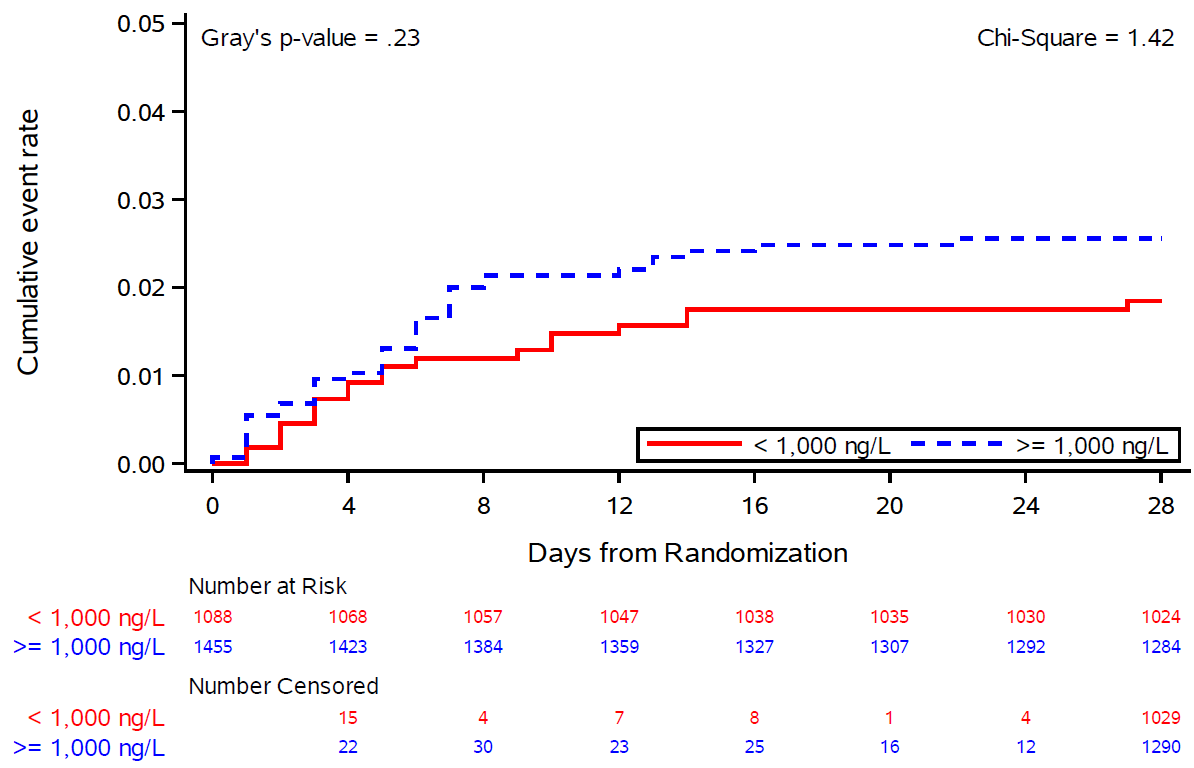
**

**Figure S6. Cumulative incidence of extrapulmonary complications (hematological category) by baseline plasma nucleocapsid antigen using a cutoff of 1,000 ng/L.**

**
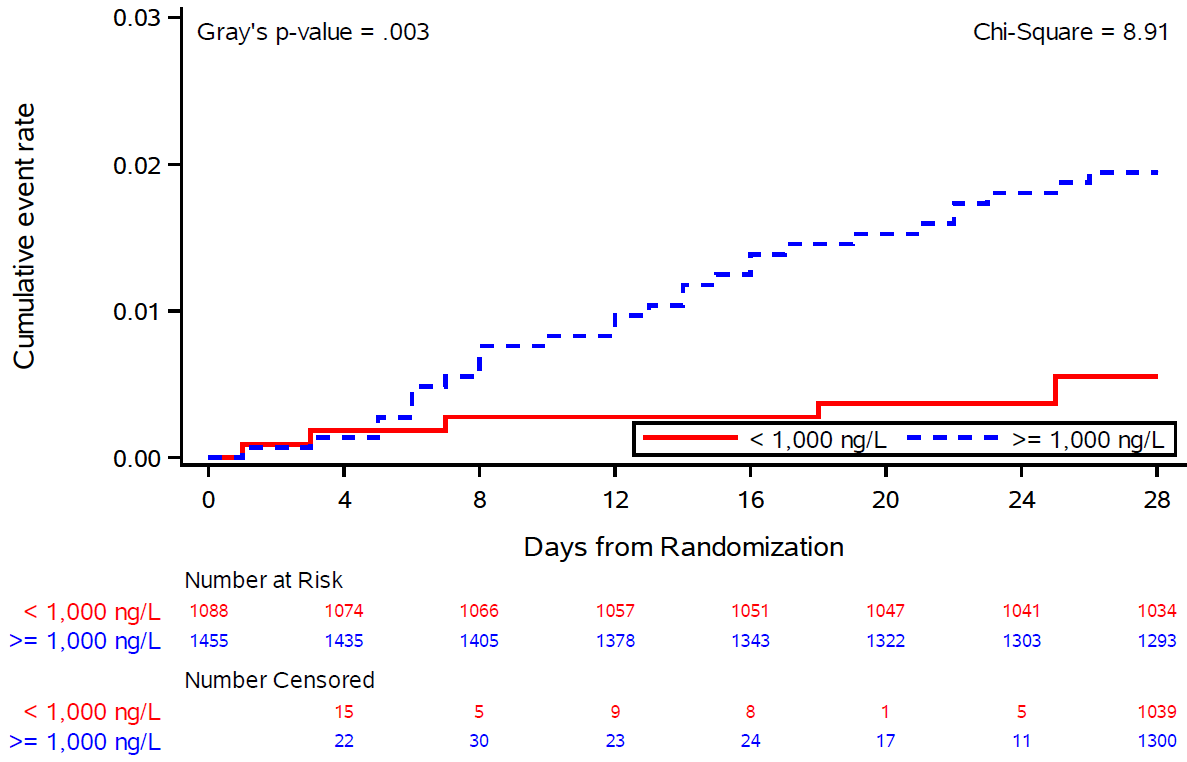
**

**Figure S7. Cumulative incidence of extrapulmonary complications (hepatic category) by baseline plasma nucleocapsid antigen using a cutoff of 1,000 ng/L.**

**
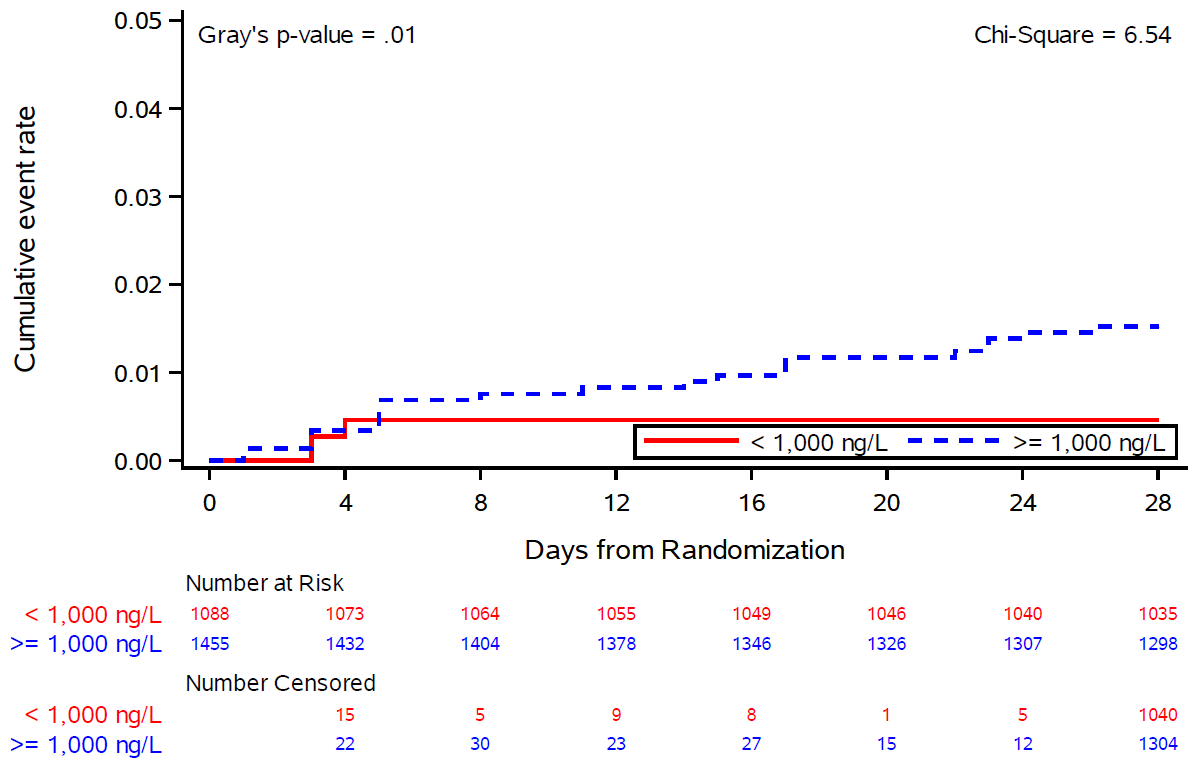
**

**Figure S8. Cumulative incidence of extrapulmonary complications (infectious category) by baseline plasma nucleocapsid antigen using a cutoff of 1,000 ng/L.**

**
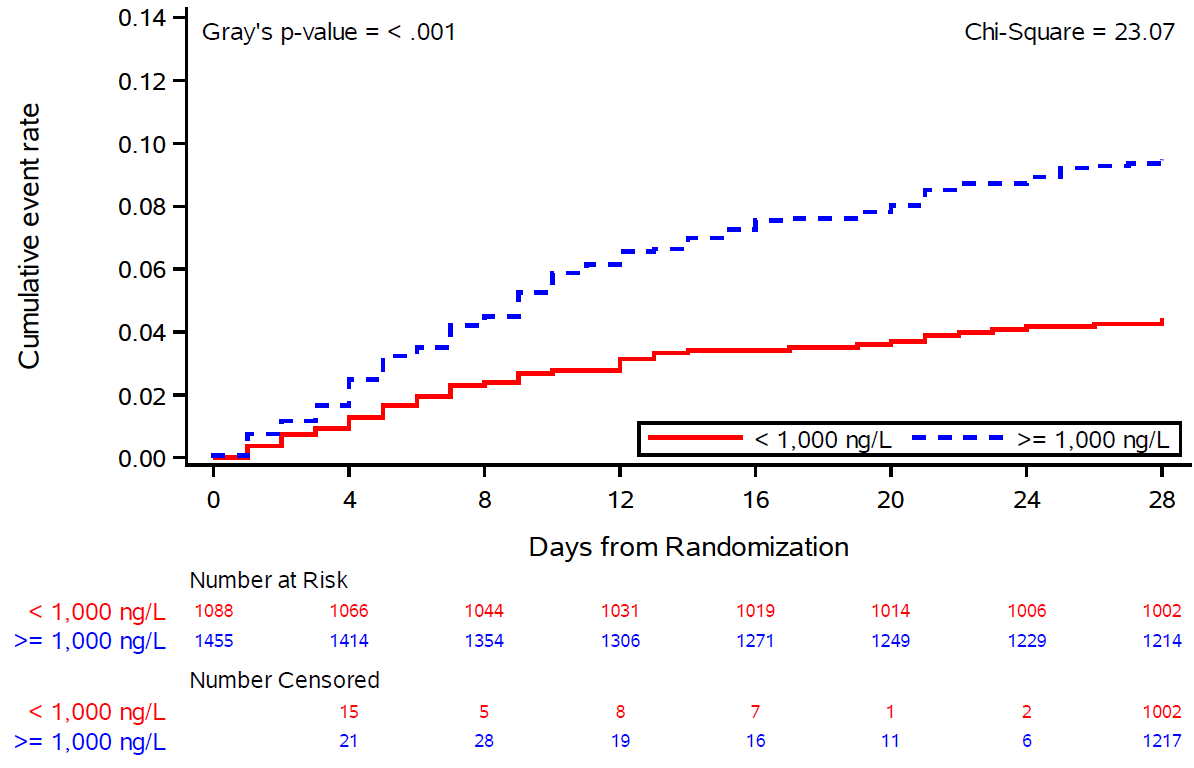
**

**Figure S9. Cumulative incidence of extrapulmonary complications (miscellaneous category) by baseline plasma nucleocapsid antigen using a cutoff of 1,000 ng/L.**

**
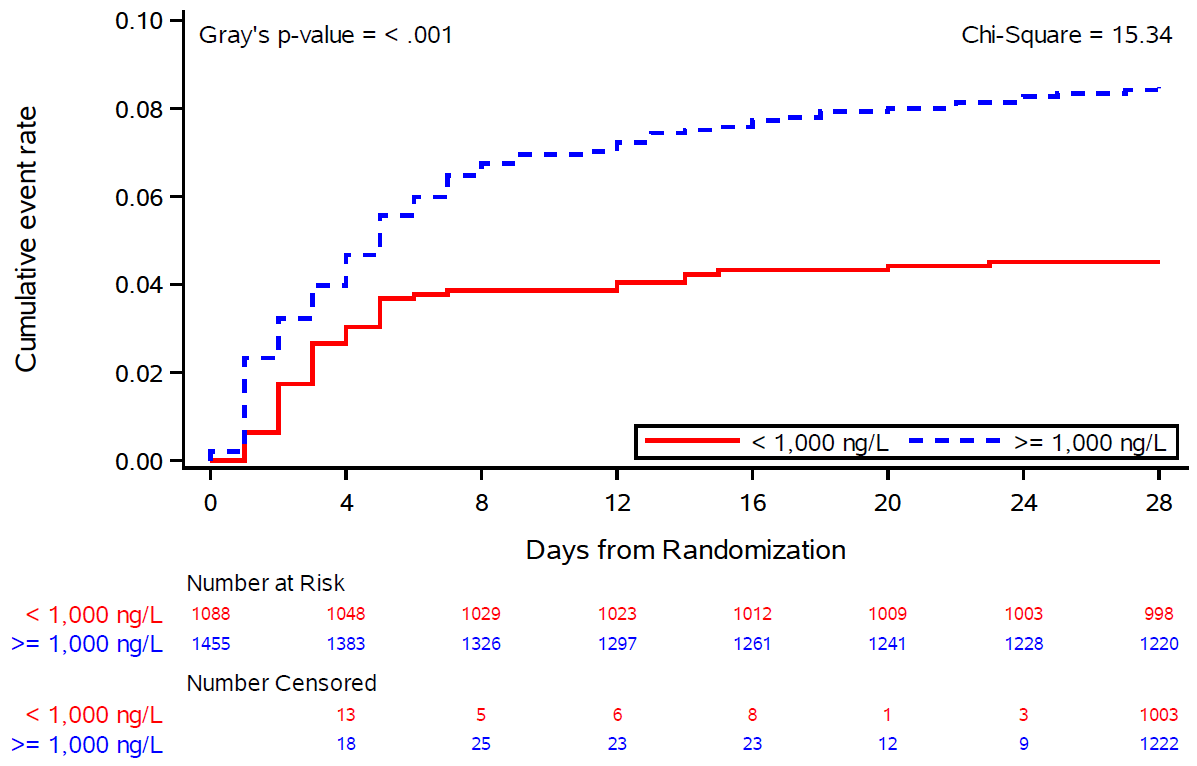
**

**Figure S10. Cumulative incidence of extrapulmonary complications (neurological category) by baseline plasma nucleocapsid antigen using a cutoff of 1,000 ng/L.**

**
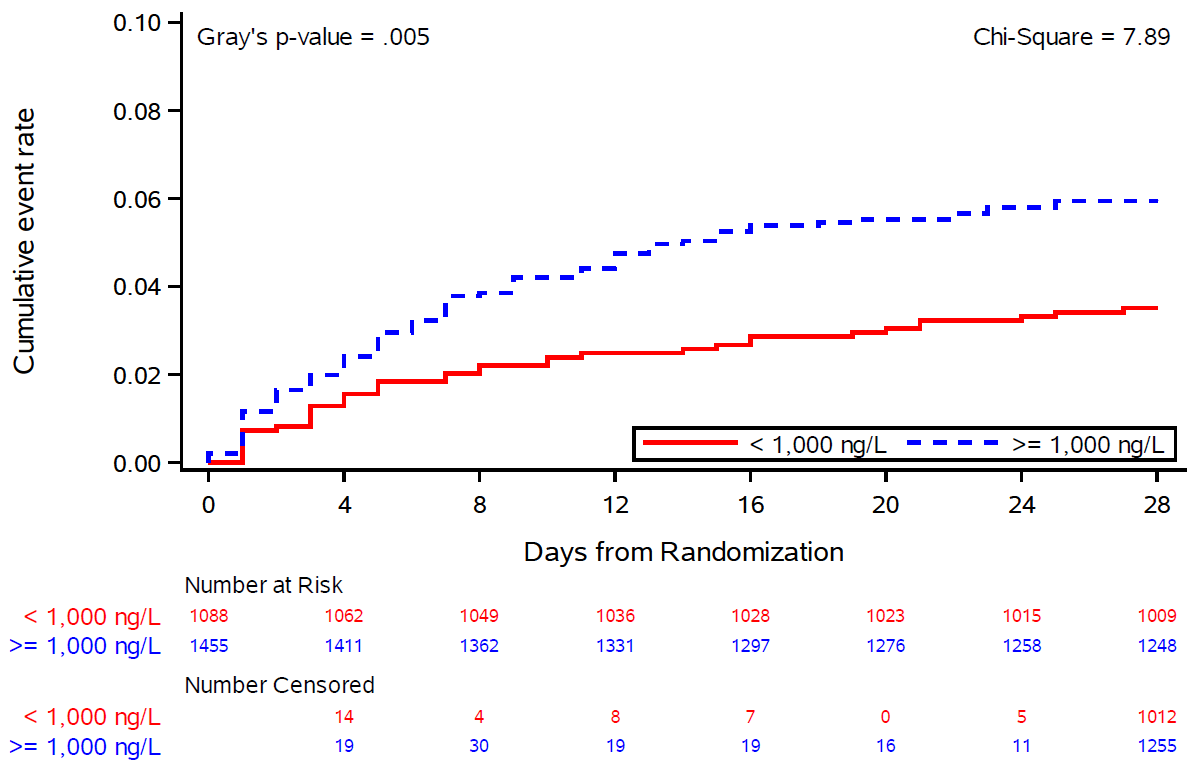
**

**Figure S11. Cumulative incidence of extrapulmonary complications (renal category) by baseline plasma nucleocapsid antigen using a cutoff of 1,000 ng/L.**

**
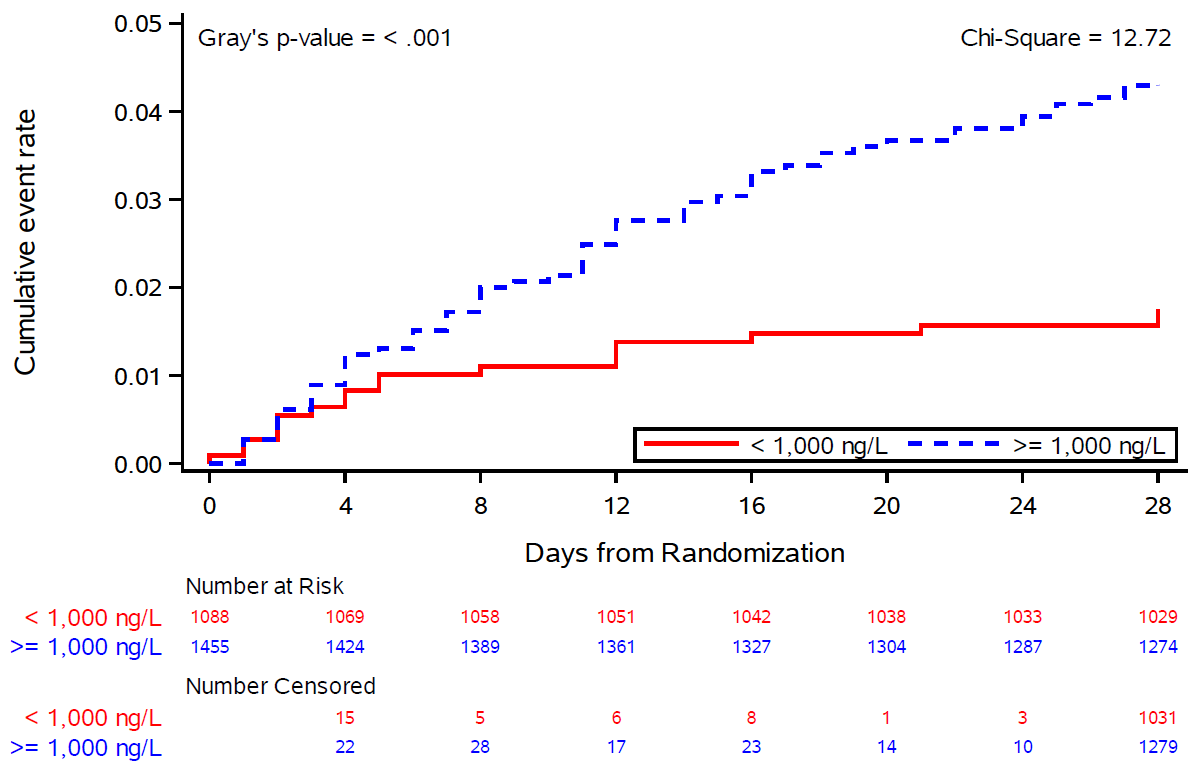
**

**Figure S12. Cumulative incidence of extrapulmonary complications (venous thromboembolism category) by baseline plasma nucleocapsid antigen using a cutoff of 1,000 ng/L.**

**
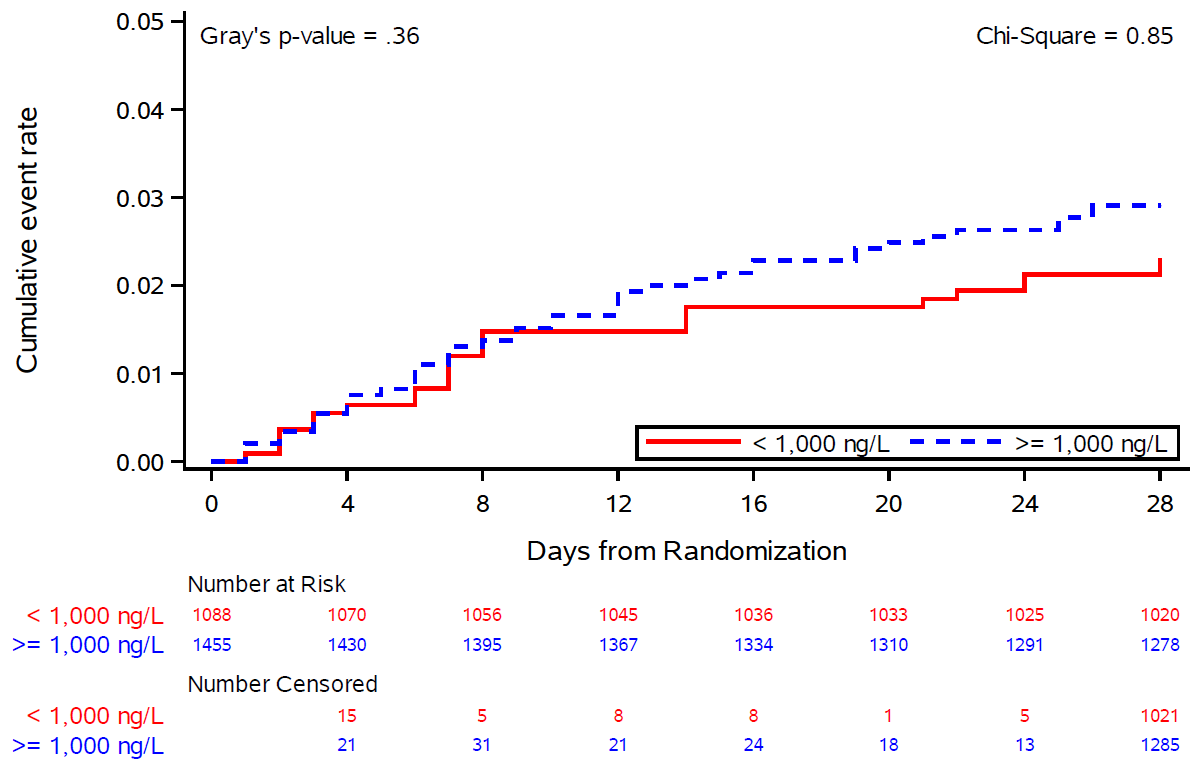
**

**Figure S13. Cumulative incidence of extrapulmonary complications (any event) by baseline plasma nucleocapsid antigen using a cutoff of 1,500 ng/L.**

**
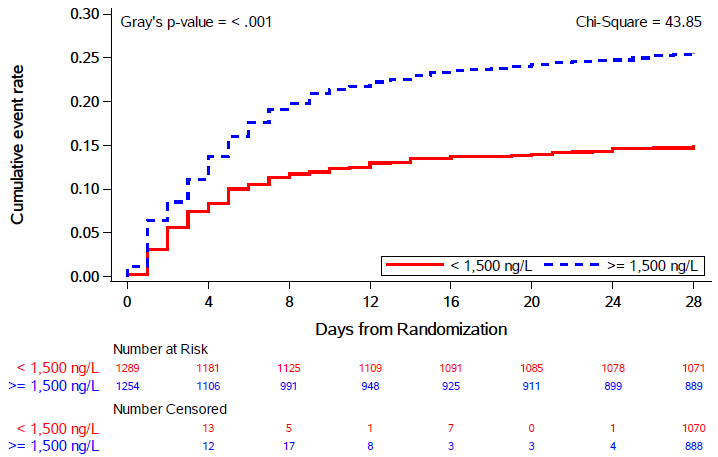
**

**Figure S14. Cumulative incidence of extrapulmonary complications (cardiovascular category) by baseline plasma nucleocapsid antigen using a cutoff of 1,500 ng/L.**

**
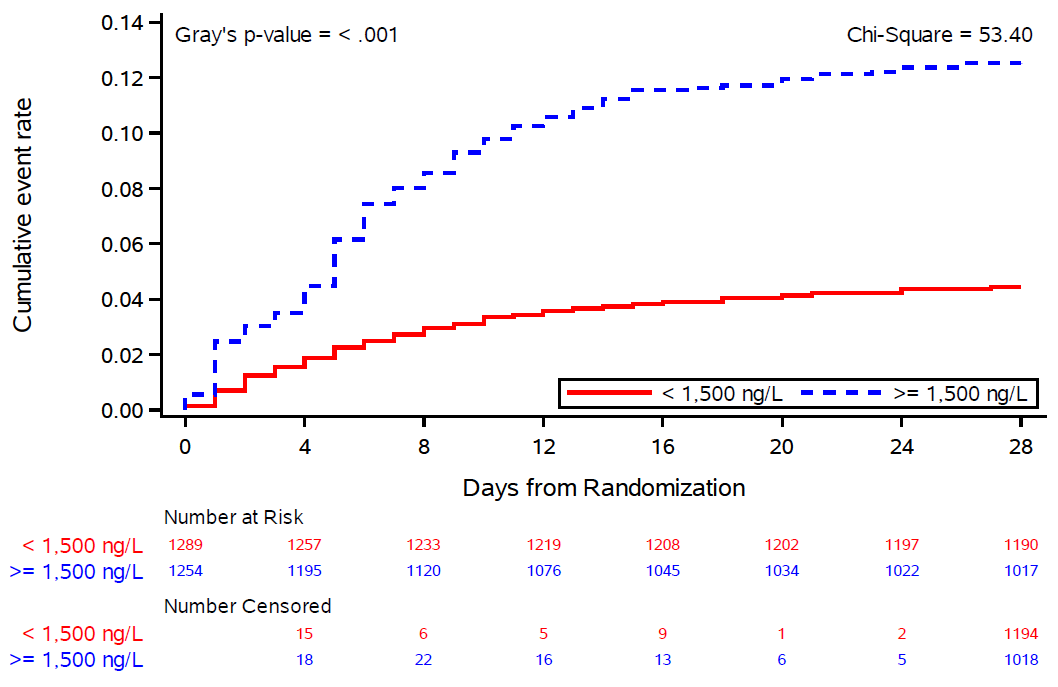
**

**Figure S15. Cumulative incidence of extrapulmonary complications (gastrointestinal category) by baseline plasma nucleocapsid antigen using a cutoff of 1,500 ng/L.**

**
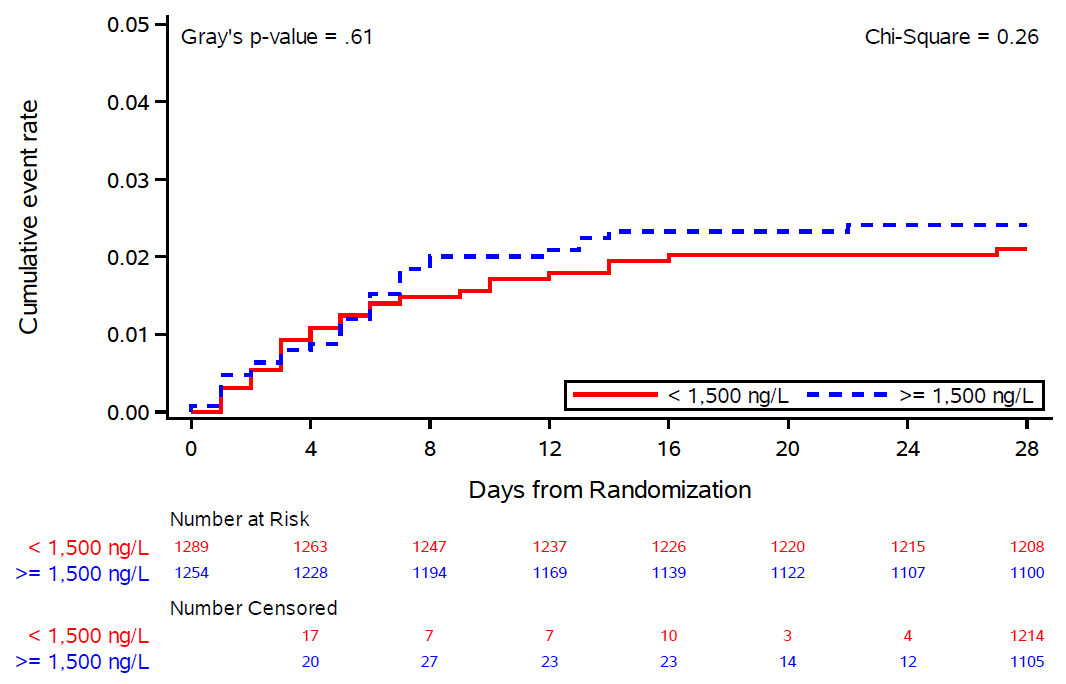
**

**Figure S16. Cumulative incidence of extrapulmonary complications (hematological category) by baseline plasma nucleocapsid antigen using a cutoff of 1,500 ng/L.**

**
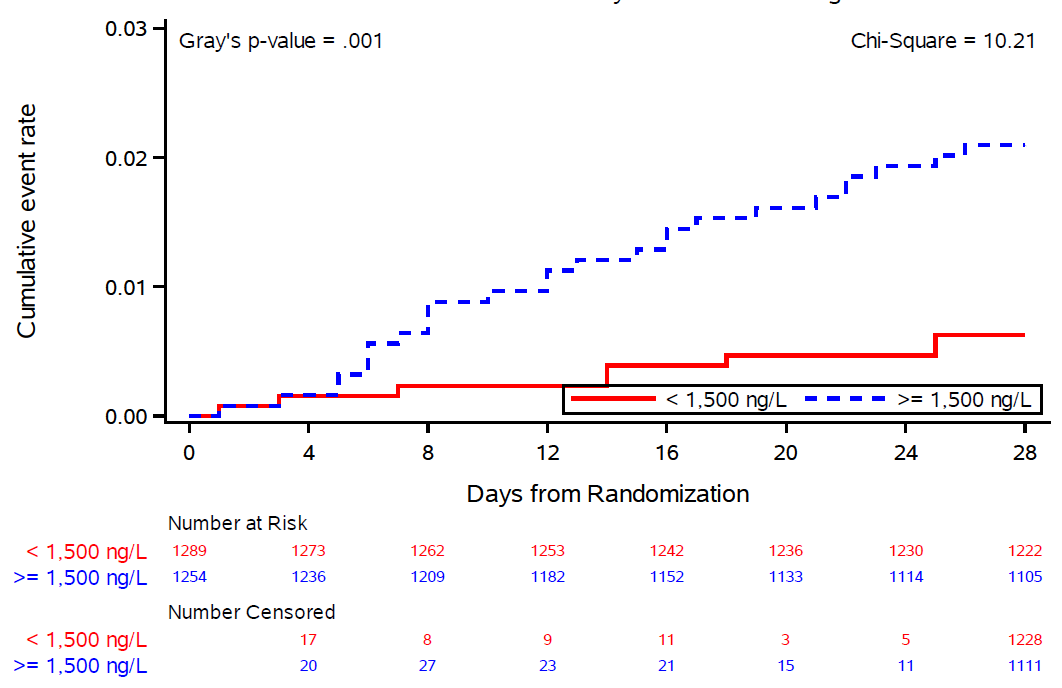
**

**Figure S17. Cumulative incidence of extrapulmonary complications (hepatic category) by baseline plasma nucleocapsid antigen using a cutoff of 1,500 ng/L.**

**
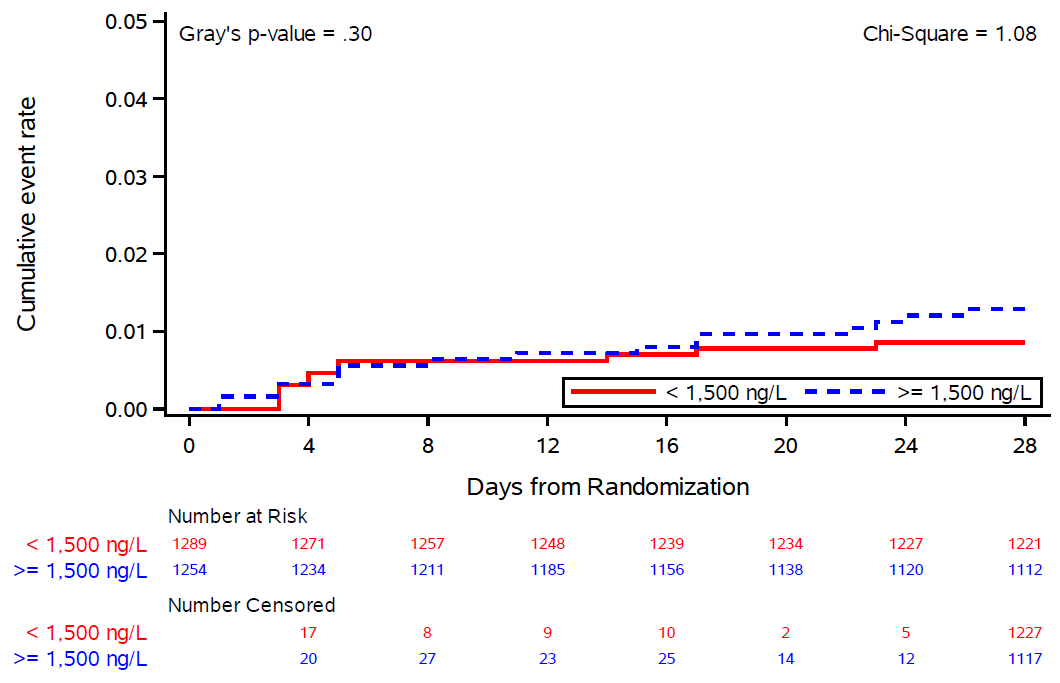
**

**Figure S18. Cumulative incidence of extrapulmonary complications (infectious category) by baseline plasma nucleocapsid antigen using a cutoff of 1,500 ng/L.**

**
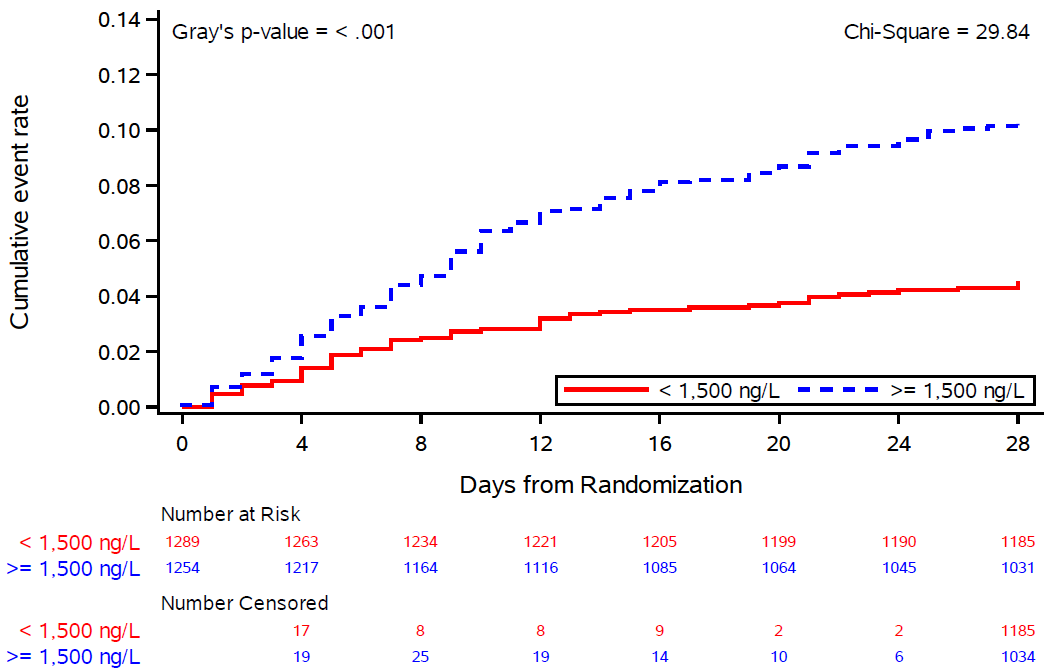
**

**Figure S19. Cumulative incidence of extrapulmonary complications (miscellaneous category) by baseline plasma nucleocapsid antigen using a cutoff of 1,500 ng/L.**

**
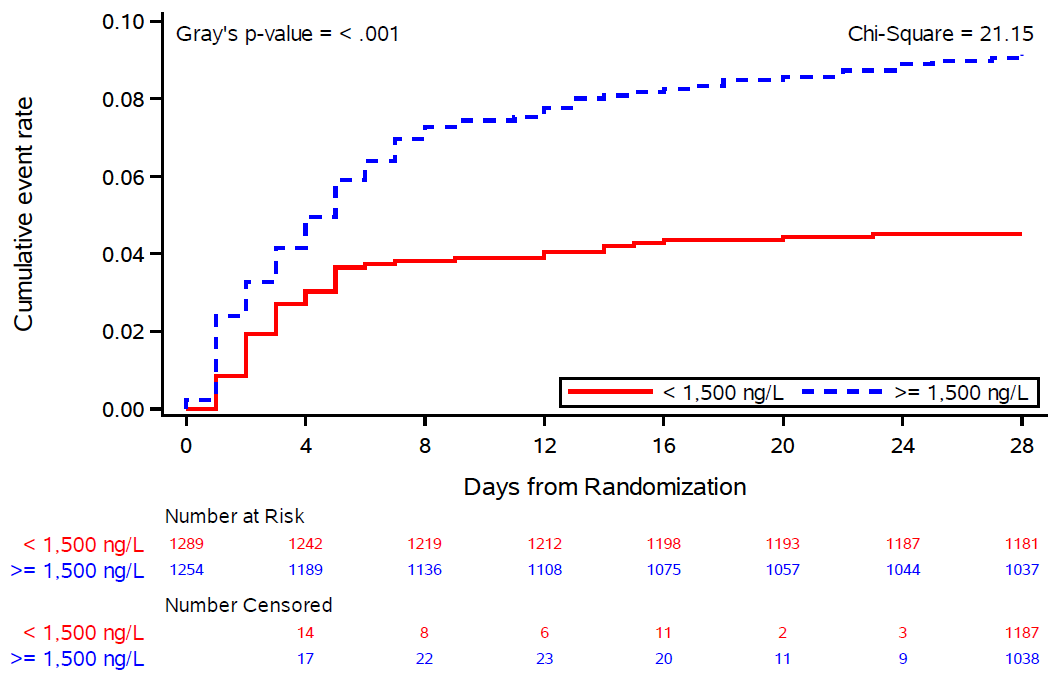
**

**Figure S20. Cumulative incidence of extrapulmonary complications (neurological category) by baseline plasma nucleocapsid antigen using a cutoff of 1,500 ng/L.**

**
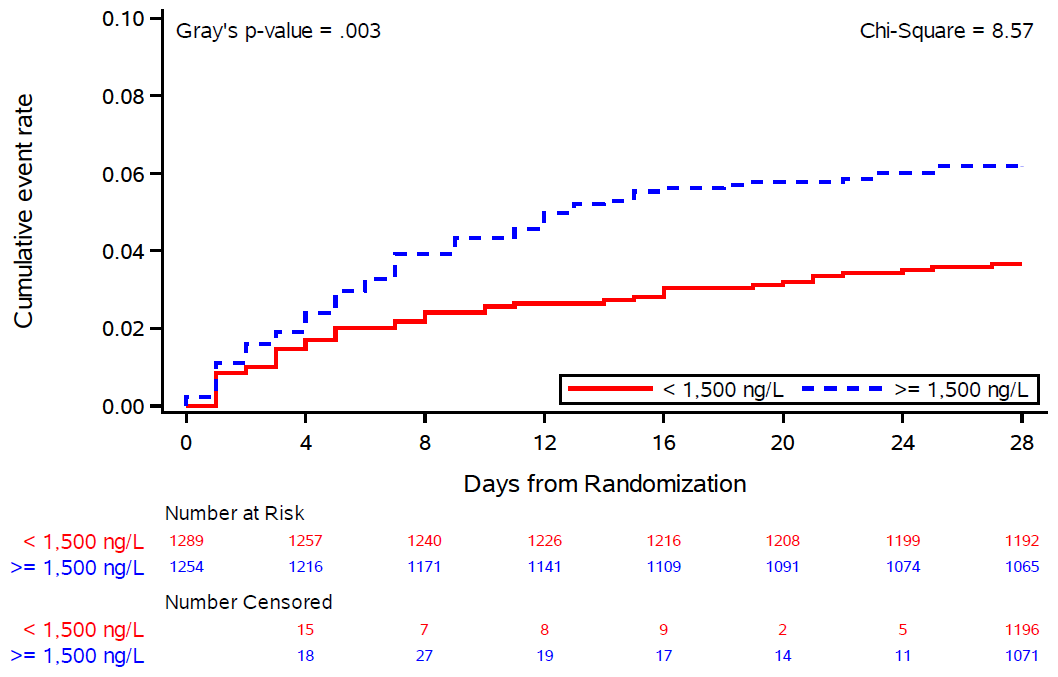
**

**Figure S21. Cumulative incidence of extrapulmonary complications (renal category) by baseline plasma nucleocapsid antigen using a cutoff of 1,500 ng/L.**

**
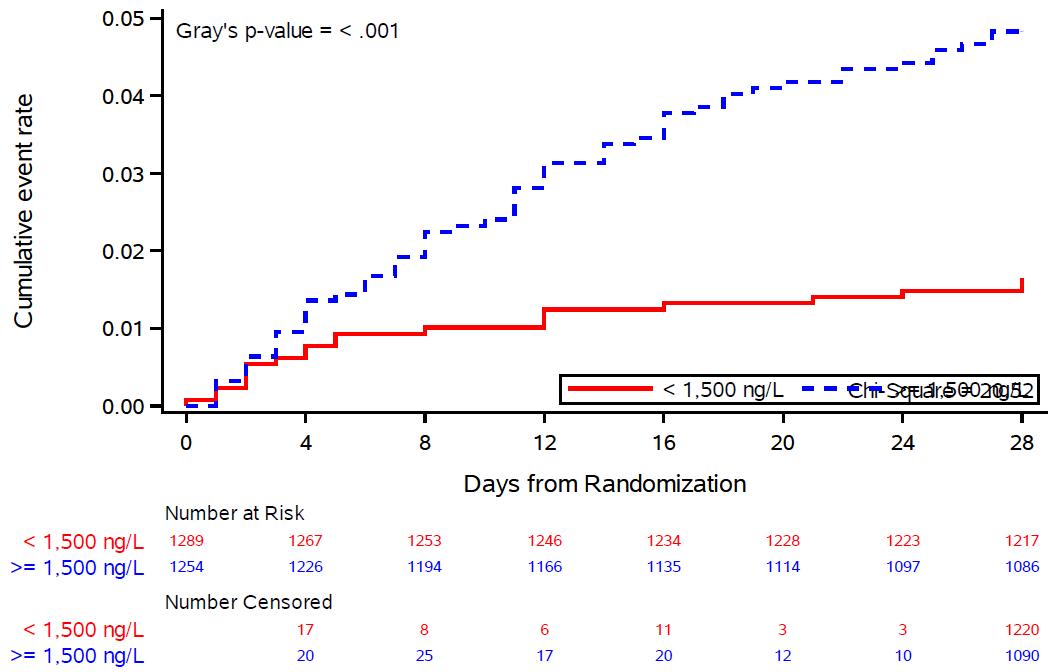
**

**Figure S22. Cumulative incidence of extrapulmonary complications (venous thromboembolism category) by baseline plasma nucleocapsid antigen using a cutoff of 1,500 ng/L.**

**
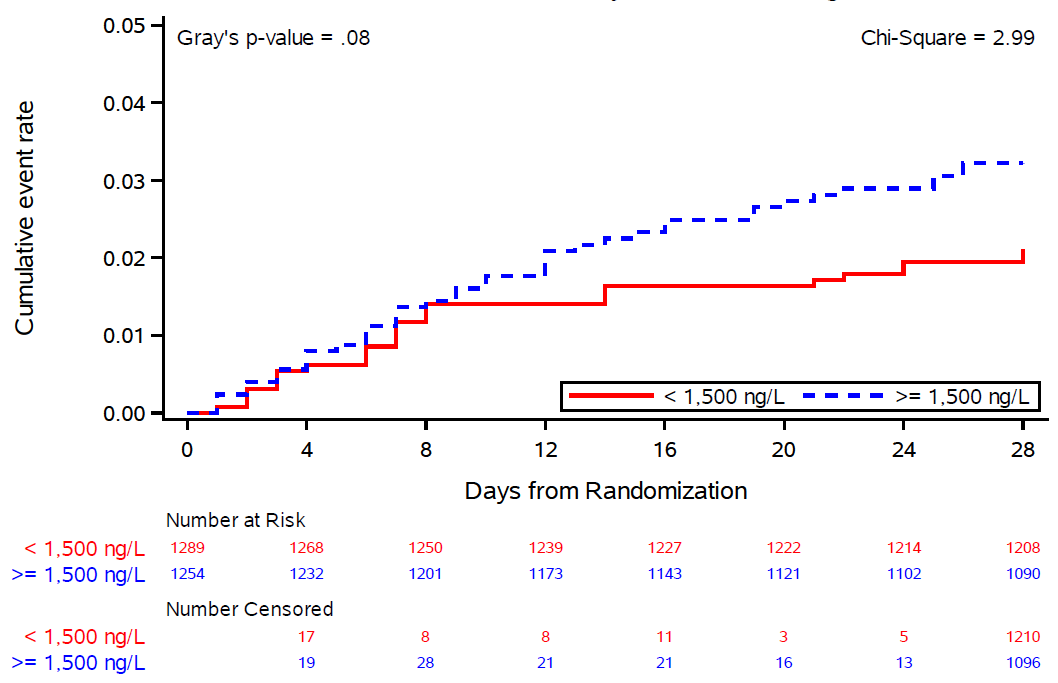
**

**Figure S23. Cumulative incidence of extrapulmonary complications (any event) by baseline plasma nucleocapsid antigen using rounded quartile cutoffs with breakpoints at 200, 1,500, and 4,500 ng/L.**

**
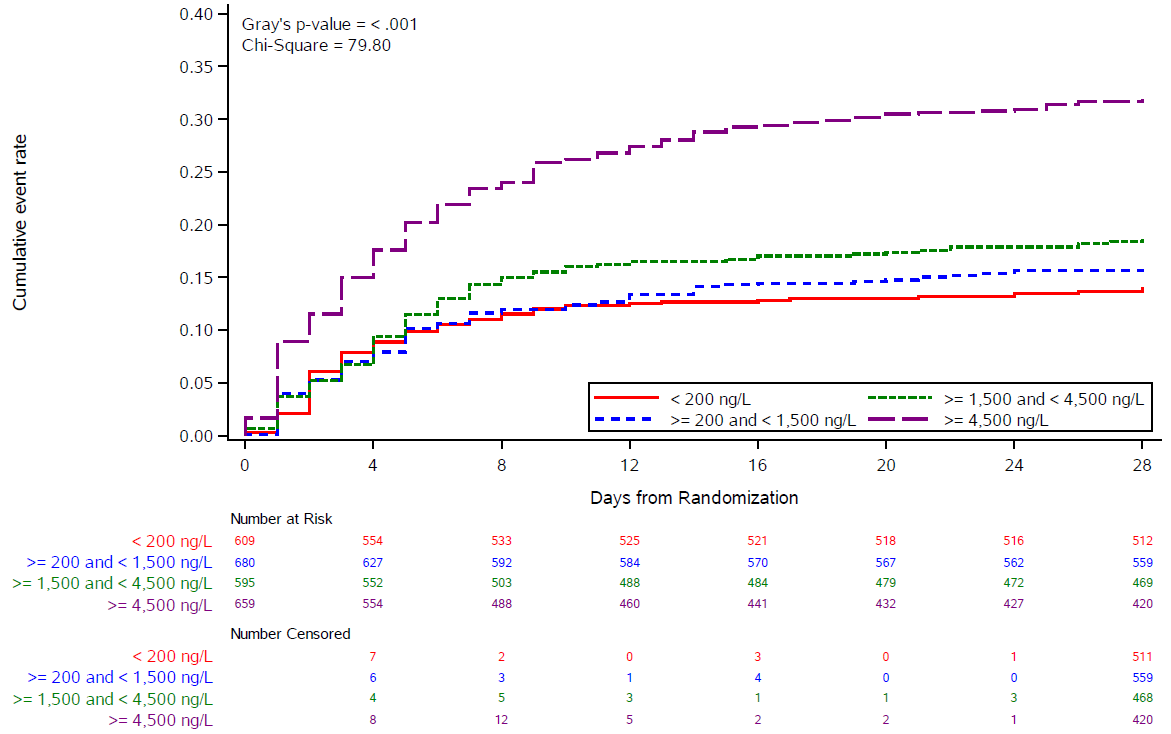
**

**Figure S24. Cumulative incidence of extrapulmonary complications (cardiovascular category) by baseline plasma nucleocapsid antigen using rounded quartile cutoffs with breakpoints at 200, 1,500, and 4,500 ng/L.**

**
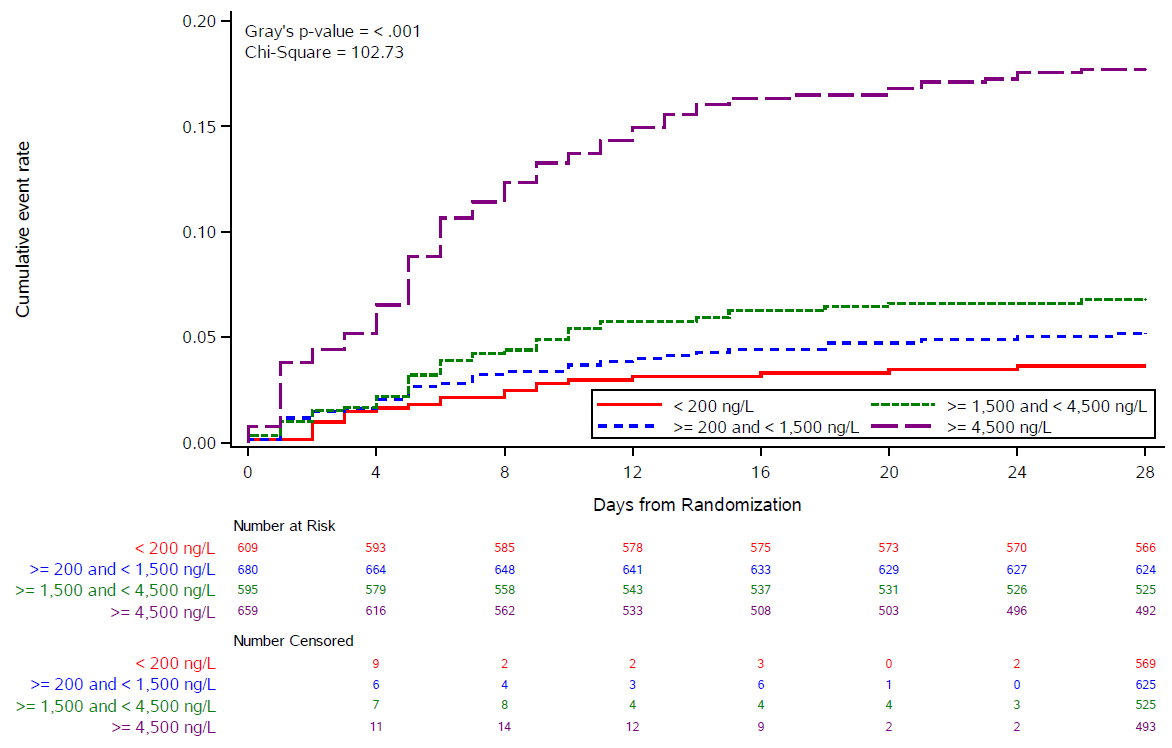
**

**Figure S25. Cumulative incidence of extrapulmonary complications (gastrointestinal category) by baseline plasma nucleocapsid antigen using rounded quartile cutoffs with breakpoints at 200, 1,500, and 4,500 ng/L.**

**
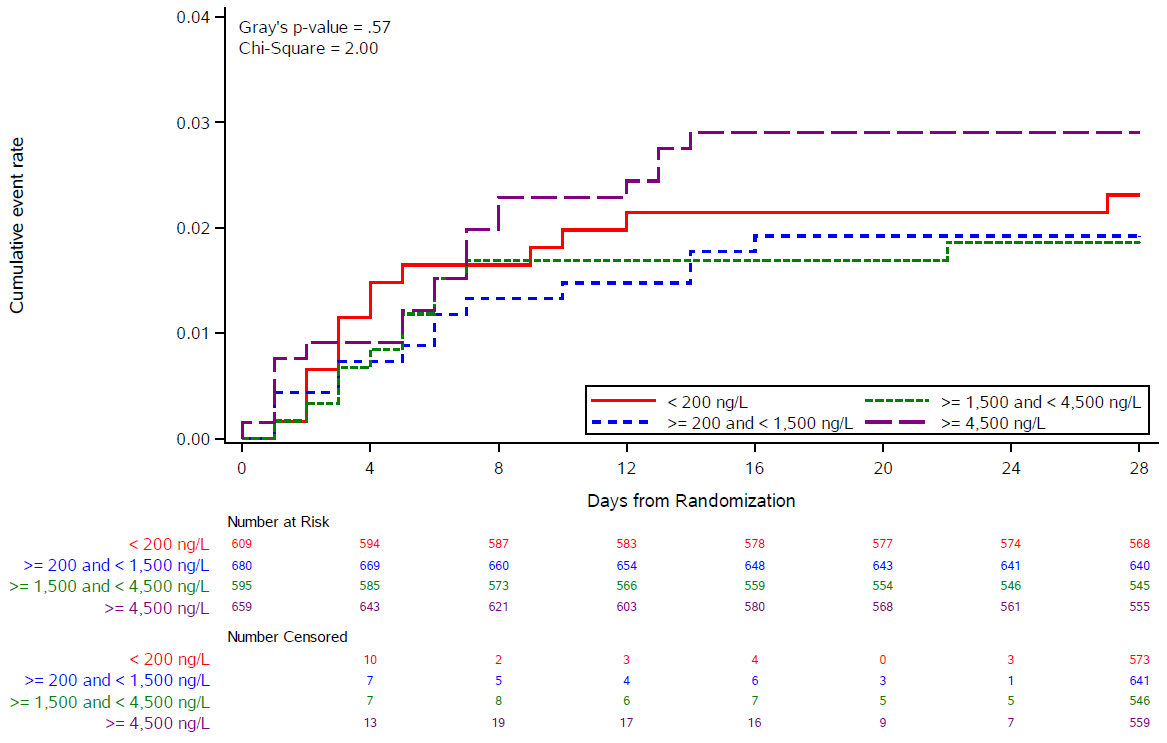
**

**Figure S26. Cumulative incidence of extrapulmonary complications (hematological category) by baseline plasma nucleocapsid antigen using rounded quartile cutoffs with breakpoints at 200, 1,500, and 4,500 ng/L..**

**
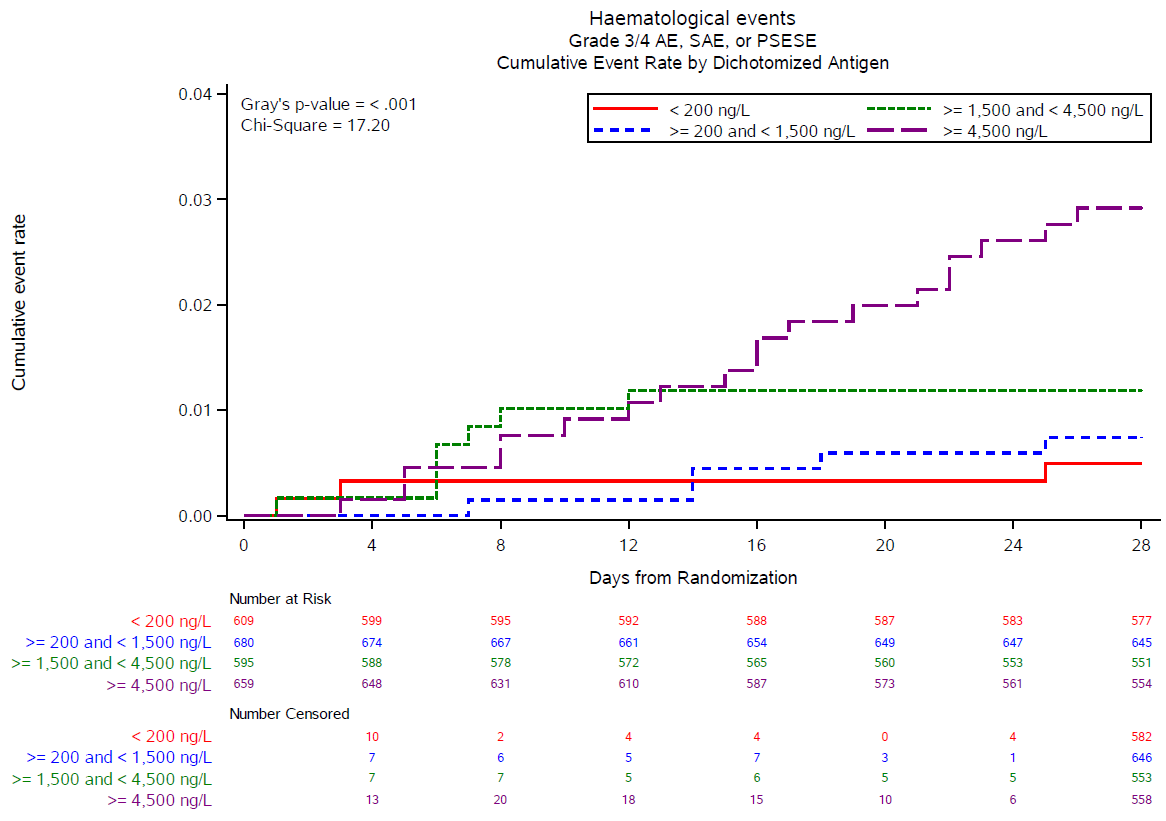
**

**Figure S27. Cumulative incidence of extrapulmonary complications (hepatic category) by baseline plasma nucleocapsid antigen using rounded quartile cutoffs with breakpoints at 200, 1,500, and 4,500 ng/L.**

**
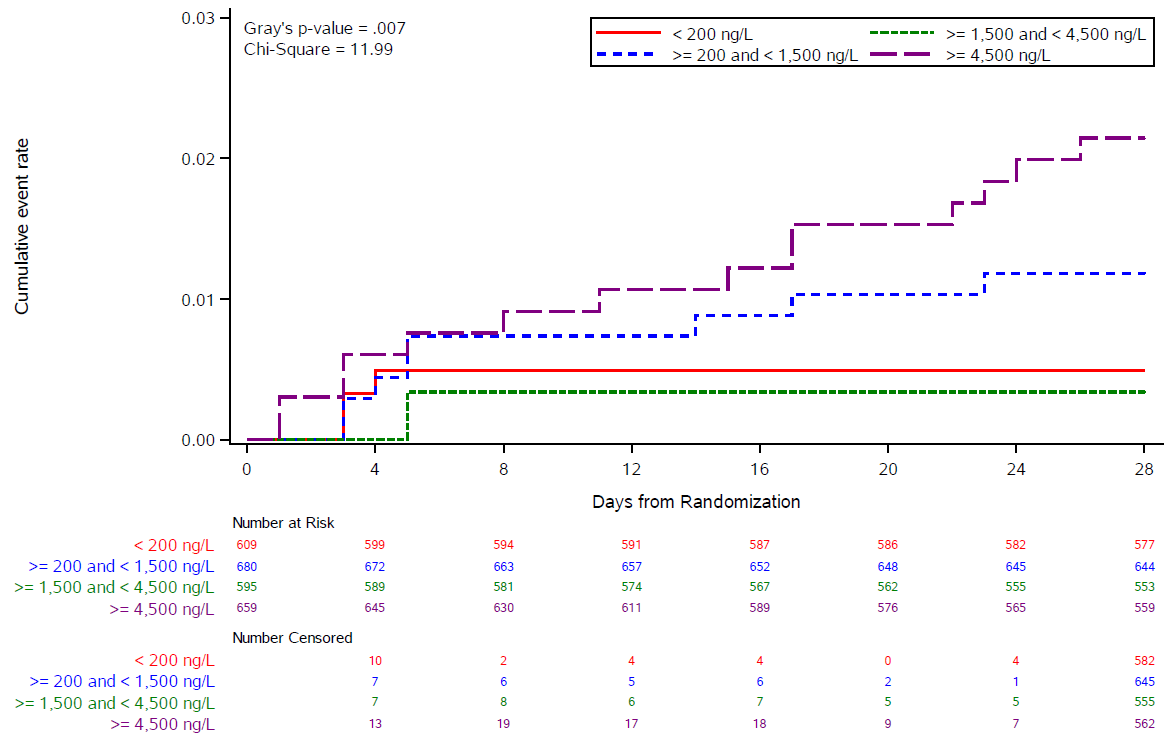
**

**Figure S28. Cumulative incidence of extrapulmonary complications (infectious category) by baseline plasma nucleocapsid antigen using rounded quartile cutoffs with breakpoints at 200, 1,500, and 4,500 ng/L.**

**
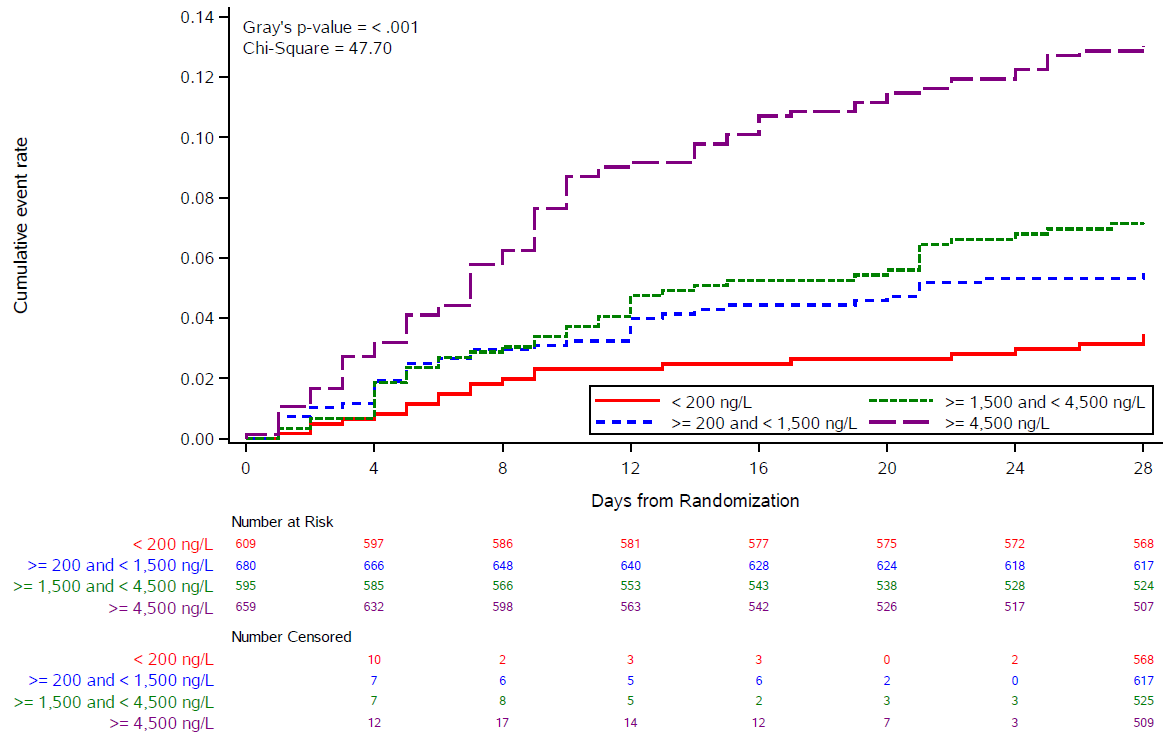
**

**Figure S29. Cumulative incidence of extrapulmonary complications (miscellaneous category) by baseline plasma nucleocapsid antigen using rounded quartile cutoffs with breakpoints at 200, 1,500, and 4,500 ng/L.**

**
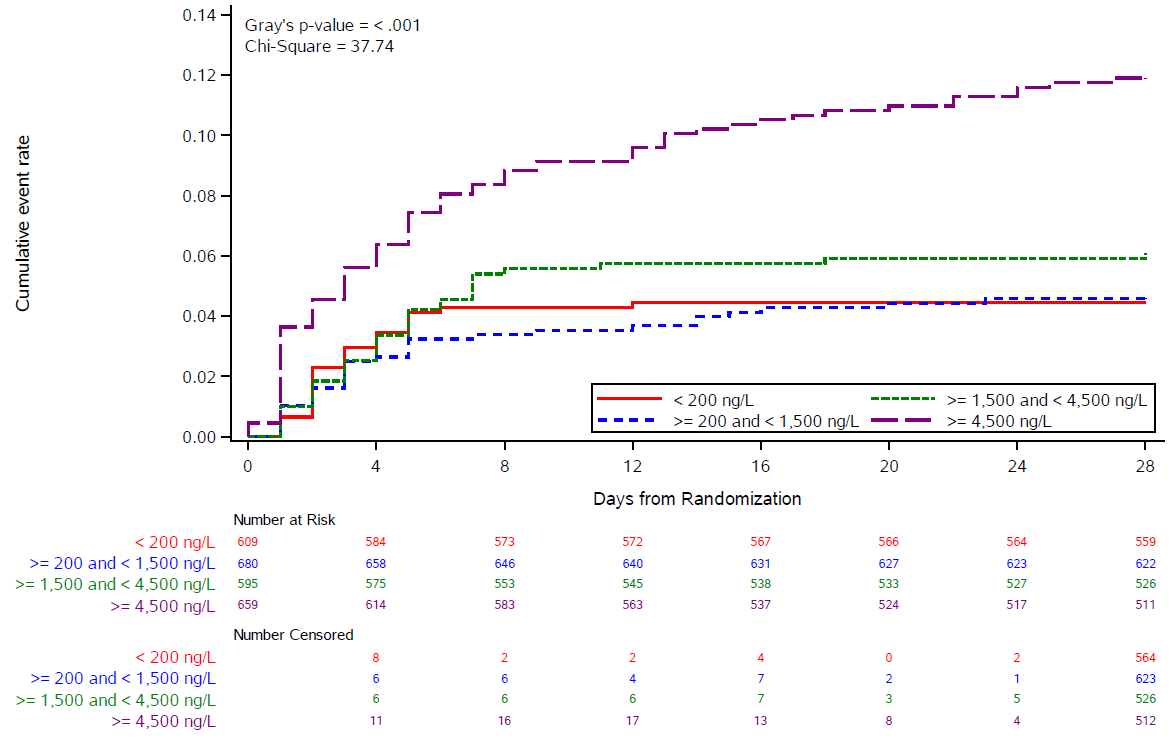
**

**Figure S30. Cumulative incidence of extrapulmonary complications (neurological category) by baseline plasma nucleocapsid antigen using rounded quartile cutoffs with breakpoints at 200, 1,500, and 4,500 ng/L.**

**
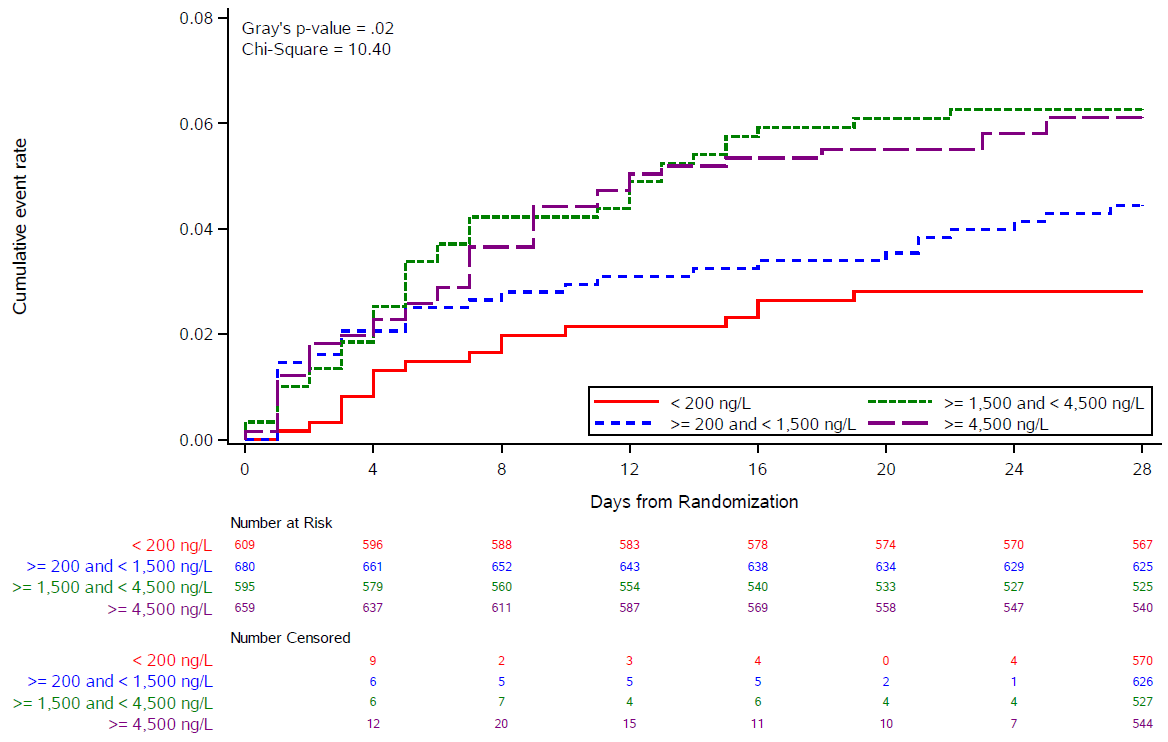
**

**Figure S31. Cumulative incidence of extrapulmonary complications (renal category) by baseline plasma nucleocapsid antigen using rounded quartile cutoffs with breakpoints at 200, 1,500, and 4,500 ng/L.**

**
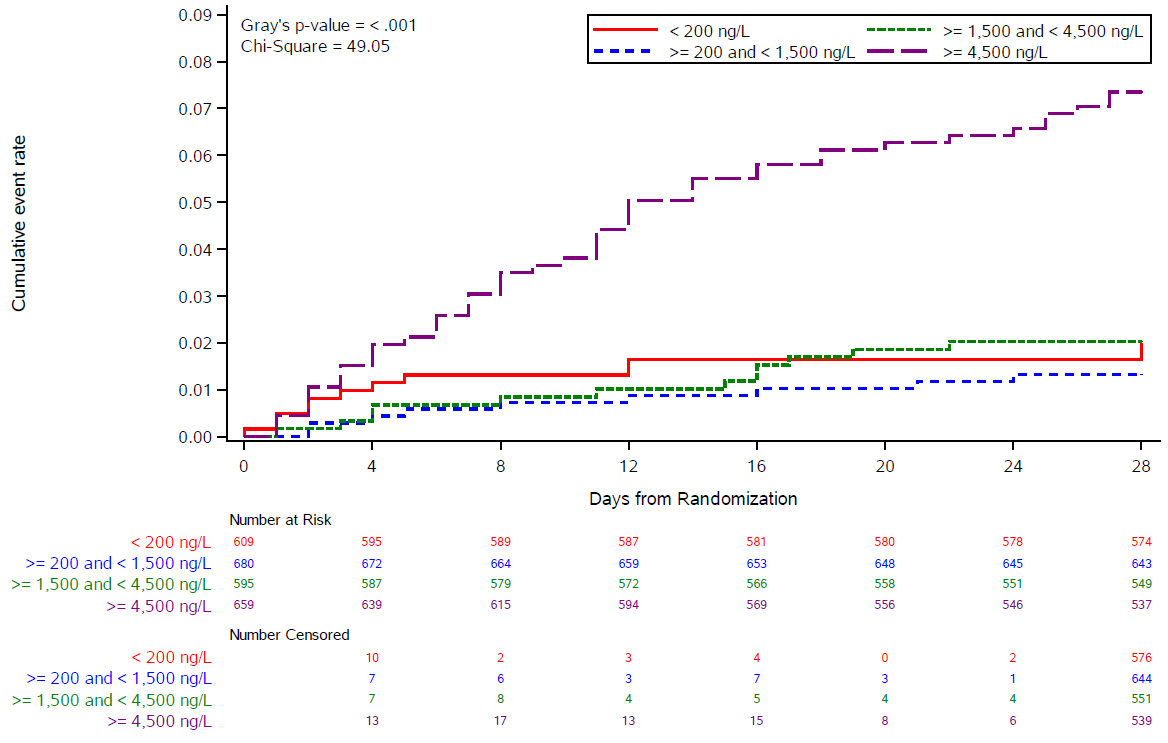
**

**Figure S32. Cumulative incidence of extrapulmonary complications (venous thromboembolism category) by baseline plasma nucleocapsid antigen using rounded quartile cutoffs with breakpoints at 200, 1,500, and 4,500 ng/L.**

**
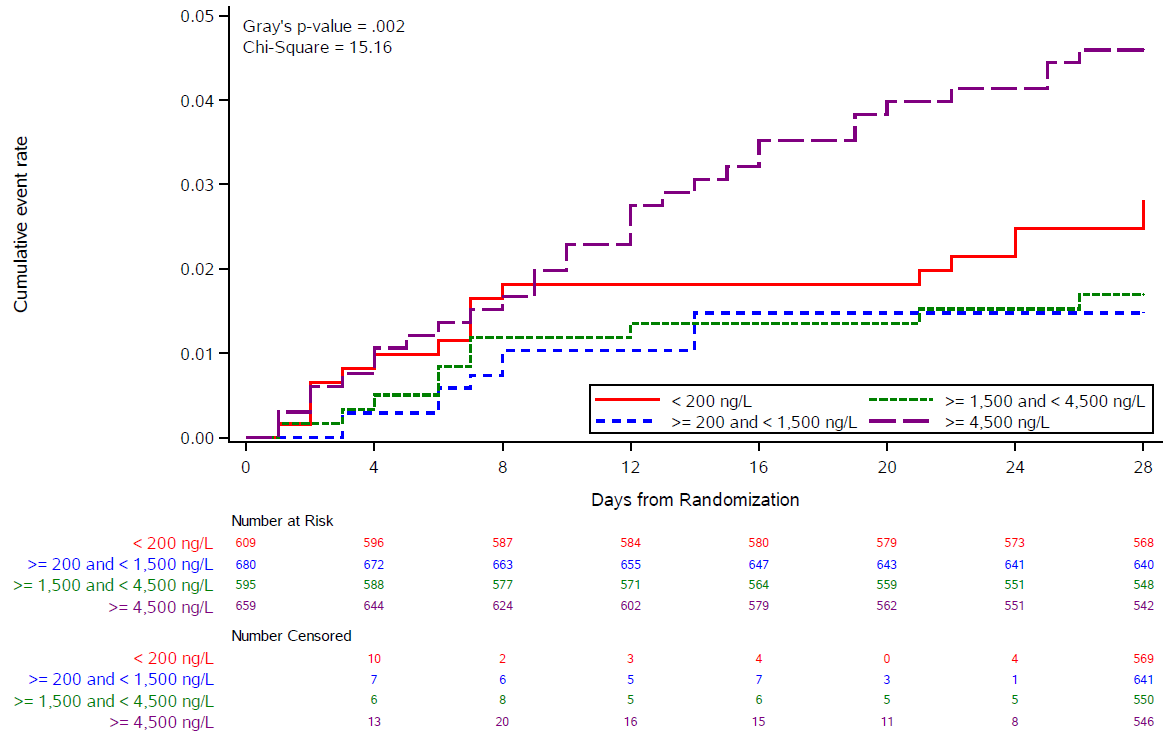
**

**Figure S33. Relationship between increasing plasma N-Ag and the HR of having at least one EPC of any type.**

**
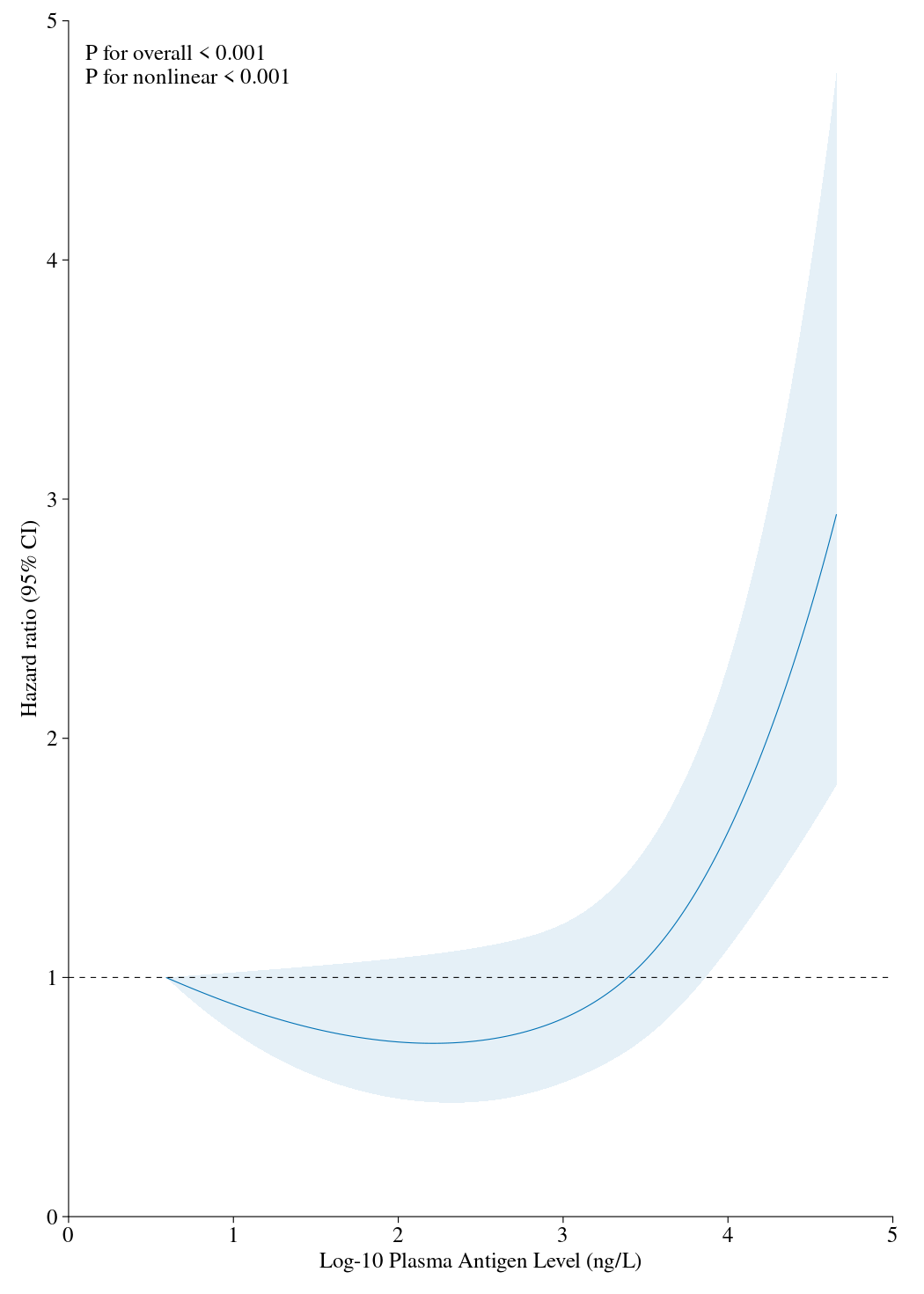
**

**Table S1. Definitions of baseline comorbidities.**

| Comorbidities |  |
| --- | --- |
| Cardiovascular disease | - Cerebrovascular event (thrombotic or hemorrhagic) - Heart failure - Hypertension requiring medications - Myocardial infarction (MI) or other acute coronary syndrome |
| Chronic kidney disease | - Renal impairment - Renal replacement therapy prior to COVID-19 |
| Chronic lung disease | - Asthma - Chronic obstructive lung disease - Chronic continuous oxygen supplementation |
| Diabetes | - Diabetes mellitus requiring medication |
| Hepatic impairment | - Hepatic impairment |
| HIV | - HIV |
| Immunocompromise | - Immunosuppressive disorder other than HIV - Malignancy (active or receiving treatment) - Treatment with antirejection medications (antirejection medicine after solid or stem cell transplant) - Treatment with biological medications (Biological medicine to treat autoimmune disease or cancer) - Treatment with immune modulators (Interleukin-1 inhibitors, Interleukin-6 inhibitors, Interferons, Janus kinase inhibitors, TNF inhibitors, others) |
| Obesity | - Body mass index of 30 kg/m2 or above |

**Table S2. The 7-category pulmonary ordinal outcome scale.**

| 1. Can independently undertake usual activities with minimal or no symptoms  2. Symptomatic and currently unable to independently undertake usual activities but no need of supplemental oxygen (or not above premorbid requirements)  3. Supplemental oxygen (<4 liters/min, or <4 liters/min above premorbid requirements)  4. Supplemental oxygen (≥4 liters/min, or ≥4 liters/min above premorbid requirements, but not high-flow oxygen)  5. Non-invasive ventilation or high-flow oxygen (high flow nasal cannula)  6. Invasive ventilation, extracorporeal membrane oxygenation (ECMO), mechanical circulatory support, or new receipt of renal replacement therapy  7. Death |
| --- |

**Table S3. Multivariable analysis of associations between baseline plasma nucleocapsid antigen, other baseline factors, and extrapulmonary complications in total and by event category.**

|  | **Any** | **Cardiovascular** | **Gastrointestinal** | **Hematological** | **Hepatic** | **Infectious** | **Miscellaneous** | **Neurological** | **Renal** | **Venous thrombo-embolism** |
| --- | --- | --- | --- | --- | --- | --- | --- | --- | --- | --- |
| **Baseline factor** | HR (95% CI) | HR (95% CI) | HR (95% CI) | HR (95% CI) | HR (95% CI) | HR (95% CI) | HR (95% CI) | HR (95% CI) | HR (95% CI) | HR (95% CI) |
| **Plasma N-Ag (log_10_)** | 1.21 (1.09, 1.34) | 1.63 (1.35, 1.96) | 1.04 (0.78, 1.39) | 1.79 (1.10, 2.92) | 1.47 (0.89, 2.44) | 1.42 (1.17, 1.71) | 1.29 (1.07, 1.56) | 1.21 (0.98, 1.50) | 1.41 (1.05, 1.90) | 0.96 (0.74, 1.25) |
| **Age^a^** | 1.17 (1.09, 1.26) | 1.28 (1.14, 1.43) | 1.05 (0.86, 1.28) | 1.52 (1.13, 2.03) | 0.84 (0.62, 1.14) | 1.29 (1.14, 1.45) | 1.14 (1.01, 1.28) | 1.36 (1.18, 1.57) | 1.33 (1.10, 1.61) | 1.10 (0.91, 1.35) |
| **Male sex** | 0.81 (0.68, 0.97) | 0.91 (0.69, 1.21) | 0.57 (0.33, 0.99) | 0.90 (0.44, 1.84) | 1.78 (0.70, 4.55) | 1.10 (0.81, 1.50) | 0.83 (0.61, 1.13) | 0.68 (0.47, 0.97) | 0.82 (0.51, 1.30) | 1.13 (0.68, 1.88) |
| **Comorbidities** |  |  |  |  |  |  |  |  |  |  |
| CVD | 1.10 (0.90, 1.35) | 0.97 (0.71, 1.32) | 1.23 (0.68, 2.23) | 0.58 (0.26, 1.26) | 0.71 (0.27, 1.83) | 1.08 (0.77, 1.50) | 1.11 (0.79, 1.56) | 1.21 (0.80, 1.82) | 1.06 (0.62, 1.81) | 1.08 (0.62, 1.88) |
| CKD | 1.42 (1.10, 1.85) | 1.55 (1.03, 2.31) | 0.72 (0.27, 1.89) | 1.28 (0.48, 3.43) | 1.10 (0.31, 3.84) | 1.10 (0.72, 1.70) | 0.83 (0.50, 1.38) | 1.55 (0.95, 2.53) | 2.77 (1.60, 4.79) | 0.95 (0.41, 2.21) |
| Chronic lung disease | 1.37 (1.10, 1.72) | 1.30 (0.90, 1.87) | 1.00 (0.48, 2.05) | 1.98 (0.87, 4.51) | 1.75 (0.64, 4.82) | 1.59 (1.10, 2.28) | 1.42 (0.98, 2.08) | 1.36 (0.88, 2.10) | 1.48 (0.86, 2.58) | 1.64 (0.90, 2.99) |
| Diabetes | 1.01 (0.83, 1.24) | 1.30 (0.95, 1.77) | 1.26 (0.70, 2.29) | 1.81 (0.85, 3.88) | 1.39 (0.53, 3.69) | 1.08 (0.77, 1.51) | 1.06 (0.75, 1.50) | 0.93 (0.62, 1.39) | 1.31 (0.80, 2.15) | 1.45 (0.84, 2.50) |
| Immunocompromise | 1.40 (1.12, 1.75) | 1.38 (0.99, 1.93) | 1.16 (0.57, 2.34) | 3.59 (1.67, 7.70) | 2.39 (0.97, 5.89) | 1.82 (1.29, 2.59) | 1.32 (0.90, 1.95) | 0.96 (0.59, 1.58) | 1.60 (0.95, 2.70) | 1.22 (0.65, 2.28) |
| Obesity | 1.07 (0.89, 1.29) | 1.06 (0.79, 1.42) | 1.21 (0.69, 2.14) | 0.46 (0.21, 1.00) | 0.15 (0.05, 0.46) | 0.83 (0.61, 1.14) | 0.96 (0.70, 1.32) | 1.41 (0.96, 2.07) | 1.18 (0.73, 1.90) | 1.09 (0.65, 1.83) |
| **Vaccination status** |  |  |  |  |  |  |  |  |  |  |
| Fully vaccinated | REF | REF | REF | REF | REF | REF | REF | REF | REF | REF |
| Partially vaccinated | 1.51 (1.01, 2.27) | 2.28 (1.10, 4.72) | 1.88 (0.60, 5.86) | 2.91 (0.61, 13.9) | 0.78 (0.13, 4.82) | 2.08 (1.12, 3.90) | 0.47 (0.20, 1.09) | 1.48 (0.69, 3.16) | 1.75 (0.66, 4.67) | 1.73 (0.48, 6.25) |
| Not vaccinated | 1.46 (1.07, 1.99) | 2.82 (1.64, 4.86) | 1.28 (0.50, 3.28) | 3.45 (1.04, 11.4) | 0.88 (0.24, 3.17) | 1.47 (0.90, 2.42) | 0.97 (0.60, 1.56) | 1.09 (0.59, 2.00) | 1.61 (0.79, 3.28) | 1.96 (0.73, 5.26) |
| **Viral variant Delta** | 1.11 (0.91, 1.36) | 1.21 (0.89, 1.65) | 1.48 (0.82, 2.66) | 1.59 (0.71, 3.60) | 0.85 (0.36, 2.02) | 1.11 (0.79, 1.55) | 1.22 (0.87, 1.72) | 0.96 (0.63, 1.46) | 1.11 (0.67, 1.86) | 1.02 (0.60, 1.76) |
| **Symptom duration** | 1.00 (0.97, 1.04) | 1.03 (0.97, 1.09) | 1.05 (0.94, 1.16) | 1.00 (0.86, 1.15) | 1.07 (0.91, 1.27) | 1.01 (0.95, 1.07) | 0.99 (0.93, 1.05) | 1.01 (0.95, 1.09) | 1.03 (0.95, 1.13) | 1.15 (1.03, 1.28) |
| **Pulmonary scale** |  |  |  |  |  |  |  |  |  |  |
| No oxygen | REF | REF | REF | REF | REF | REF | REF | REF | REF | REF |
| Oxygen < 4L/min | 0.80 (0.61, 1.04) | 1.34 (0.74, 2.40) | 0.48 (0.23, 0.99) | 1.31 (0.39, 4.40) | 1.86 (0.46, 7.55) | 1.36 (0.81, 2.31) | 0.73 (0.46, 1.17) | 0.75 (0.44, 1.26) | 0.97 (0.42, 2.23) | 1.78 (0.69, 4.59) |
| Oxygen ≥ 4 L/min | 1.20 (0.92, 1.57) | 3.65 (2.13, 6.27) | 0.44 (0.20, 0.99) | 1.29 (0.37, 4.42) | 2.98 (0.76, 11.7) | 2.14 (1.28, 3.58) | 1.19 (0.76, 1.87) | 1.11 (0.66, 1.88) | 2.10 (0.98, 4.51) | 2.31 (0.90, 5.96) |
| HFNC/NIV | 1.85 (1.35, 2.53) | 7.39 (4.16, 13.2) | 1.14 (0.48, 2.74) | 5.88 (1.74, 19.9) | 1.53 (0.26, 9.07) | 4.12 (2.34, 7.26) | 1.35 (0.78, 2.34) | 1.19 (0.59, 2.36) | 4.43 (1.94, 10.1) | 4.15 (1.49, 11.5) |
| **Corticosteroid use** | 1.24 (0.99, 1.55) | 1.10 (0.76, 1.59) | 0.76 (0.41, 1.41) | 0.77 (0.32, 1.85) | 1.22 (0.45, 3.29) | 1.17 (0.80, 1.73) | 1.23 (0.84, 1.81) | 1.22 (0.78, 1.91) | 1.04 (0.57, 1.90) | 1.79 (0.88, 3.67) |
| **Active trial agent** | 0.88 (0.74, 1.05) | 0.89 (0.67, 1.17) | 0.62 (0.37, 1.04) | 0.98 (0.48, 2.00) | 0.86 (0.39, 1.89) | 0.91 (0.68, 1.23) | 0.83 (0.62, 1.13) | 0.81 (0.57, 1.16) | 1.43 (0.89, 2.31) | 0.89 (0.55, 1.44) |
| **Inflammatory markers** |  |  |  |  |  |  |  |  |  |  |
| CRP (log_2_) | 1.07 (1.00, 1.15) | 1.03 (0.92, 1.14) | 1.11 (0.91, 1.35) | 1.19 (0.89, 1.59) | 1.10 (0.79, 1.53) | 1.11 (0.99, 1.25) | 1.03 (0.92, 1.15) | 0.99 (0.86, 1.14) | 1.17 (0.97, 1.41) | 1.16 (0.96, 1.40) |
| Interleukin-6 (log_2_) | 1.11 (1.06, 1.17) | 1.19 (1.11, 1.28) | 1.07 (0.92, 1.25) | 1.09 (0.90, 1.31) | 1.14 (0.93, 1.40) | 1.14 (1.06, 1.24) | 1.12 (1.03, 1.22) | 1.08 (0.97, 1.20) | 1.19 (1.06, 1.34) | 1.11 (0.97, 1.26) |

Abbreviations: CI, confidence interval; CVD, cardiovascular disease; CKD, chronic kidney disease; CRP, C-reactive protein; HFNO, high-flow nasal oxygen; HR, hazard ratio; N, number; N-Ag, nucleocapsid antigen; NIV, non-invasive ventilation.
^a^Age categorized in 10-year bins as age decades.

**Table S4. Multivariable analysis of associations between baseline upper airway viral load, other baseline factors, and extrapulmonary complications in total and by event category.**

|  | **Any** | **Cardiovascular** | **Gastrointestinal** | **Hematological** | **Hepatic** | **Infectious** | **Miscellaneous** | **Neurological** | **Renal** | **Venous thrombo-embolism** |
| --- | --- | --- | --- | --- | --- | --- | --- | --- | --- | --- |
| **Baseline factor** | HR (95% CI) | HR (95% CI) | HR (95% CI) | HR (95% CI) | HR (95% CI) | HR (95% CI) | HR (95% CI) | HR (95% CI) | HR (95% CI) | HR (95% CI) |
| **Upper airway VL (log_10_)** | 1.12 (1.04, 1.19) | 1.17 (1.05, 1.29) | 1.08 (0.89, 1.31) | 1.20 (0.92, 1.57) | 1.07 (0.79, 1.46) | 1.19 (1.07, 1.33) | 1.05 (0.94, 1.18) | 1.14 (1.00, 1.31) | 0.96 (0.81, 1.14) | 1.10 (0.91, 1.33) |
| **Age^a^** | 1.15 (1.07, 1.24) | 1.22 (1.09, 1.37) | 1.07 (0.87, 1.32) | 1.34 (1.00, 1.80) | 0.83 (0.61, 1.12) | 1.25 (1.11, 1.42) | 1.15 (1.02, 1.30) | 1.29 (1.11, 1.49) | 1.23 (1.02, 1.29) | 1.12 (0.91, 1.38) |
| **Male sex** | 0.84 (0.69, 1.01) | 0.94 (0.70, 1.25) | 0.72 (0.42, 1.23) | 0.97 (0.46, 2.04) | 1.70 (0.66, 4.42) | 1.18 (0.86, 1.63) | 0.92 (0.67, 1.26) | 0.65 (0.44, 0.94) | 0.89 (0.56, 1.42) | 1.43 (0.82, 2.50) |
| **Comorbidities** |  |  |  |  |  |  |  |  |  |  |
| CVD | 1.08 (0.88, 1.33) | 0.94 (0.68, 1.30) | 1.33 (0.73, 2.44) | 0.56 (0.25, 1.25) | 0.70 (0.27, 1.81) | 1.10 (0.78, 1.55) | 1.08 (0.76, 1.52) | 1.14 (0.75, 1.73) | 1.21 (0.71, 2.07) | 0.98 0.54, 1.75) |
| CKD | 1.54 (1.18, 2.01) | 2.02 (1.34, 3.05) | 0.80 (0.33, 1.98) | 1.29 (0.45, 3.71) | 1.36 (0.38, 4.82) | 0.97 (0.61, 1.54) | 1.04 (0.64, 1.69) | 1.94 (1.20, 3.15) | 3.25 (1.86, 5.69) | 0.92 (0.37, 2.26) |
| Chronic lung disease | 1.43 (1.13, 1.80) | 1.22 (0.83, 1.79) | 0.79 (0.35, 1.77) | 1.95 (0.81, 4.69) | 2.05 (0.74, 5.70) | 1.50 (1.02, 2.21) | 1.38 (0.94, 2.04) | 1.31 (0.84, 2.05) | 1.62 (0.93, 2.82) | 1.71 (0.89, 3.28) |
| Diabetes | 0.98 (0.79, 1.21) | 1.14 (0.83, 1.57) | 1.16 (0.64, 2.10) | 1.67 (0.78, 3.61) | 1.46 (0.56, 3.83) | 1.15 (0.82, 1.62) | 1.08 (0.76, 1.53) | 0.92 (0.61, 1.39) | 1.21 (0.74, 1.98) | 1.17 (0.65, 2.12) |
| Immunocompromise | 1.35 (1.07, 1.70) | 1.20 (0.85, 1.70) | 1.03 (0.51, 2.05) | 3.93 (1.83, 8.44) | 2.39 (0.95, 6.01) | 1.60 (1.11, 2.29) | 1.34 (0.91, 1.96) | 0.83 (0.50, 1.38) | 1.50 (0.89, 2.54) | 1.25 (0.65, 2.41) |
| Obesity | 1.03 (0.85, 1.26) | 1.01 (0.75, 1.36) | 1.13 (0.64, 2.00) | 0.43 (0.19, 0.96) | 0.11 (0.03, 0.38) | 0.75 (0.55, 1.04) | 0.96 (0.69, 1.33) | 1.14 (0.77, 1.68) | 0.94 (0.58, 1.52) | 0.98 (0.57, 1.69) |
| **Vaccination status** |  |  |  |  |  |  |  |  |  |  |
| Fully vaccinated | REF | REF | REF | REF | REF | REF | REF | REF | REF | REF |
| Partially vaccinated | 1.41 (0.92, 2.16) | 1.90 (0.89, 4.04) | 2.18 (0.75, 6.37) | 0.68 (0.08, 6.09) | 1.00 (0.16, 6.18) | 1.87 (0.96, 3.62) | 0.51 (0.22, 1.20) | 1.10 (0.50, 2.40) | 2.39 (0.93, 6.16) | 1.83 (0.47, 7.15) |
| Not vaccinated | 1.56 (1.14, 2.13) | 2.75 (1.65, 4.58) | 1.26 (0.52, 3.07) | 2.80 (0.91, 8.61) | 1.03 (0.28, 3.77) | 1.53 (0.94, 2.51) | 1.08 (0.67, 1.72) | 1.05 (0.59, 1.85) | 1.79 (0.88, 3.63) | 2.20 (0.80, 6.03) |
| **Viral variant Delta** | 1.05 (0.85, 1.30) | 1.21 (0.87, 1.67) | 1.47 (0.81, 2.68) | 1.14 (0.48, 2.71) | 0.79 (0.32, 1.94) | 0.99 (0.70, 1.41) | 1.22 (0.85, 1.74) | 0.97 (0.64, 1.48) | 1.19 (0.71, 1.99) | 1.07 (0.60, 1.91) |
| **Symptom duration** | 1.02 (0.98, 1.06) | 1.03 (0.97, 1.09) | 1.09 (0.98, 1.22) | 1.00 (0.87, 1.16) | 1.10 (0.93, 1.30) | 1.02 (0.96, 1.09) | 1.00 (0.94, 1.06) | 1.03 (0.95, 1.10) | 1.04 (0.95, 1.14) | 1.14 (1.02, 1.27) |
| **Pulmonary scale** |  |  |  |  |  |  |  |  |  |  |
| No oxygen | REF | REF | REF | REF | REF | REF | REF | REF | REF | REF |
| Oxygen < 4L/min | 0.85 (0.64, 1.13) | 1.51 (0.79, 2.91) | 0.52 (0.25, 1.11) | 1.19 (0.34, 4.15) | 1.84 (0.43, 7.91) | 1.44 (0.82, 2.53) | 0.82 (0.51, 1.33) | 0.88 (0.51, 1.53) | 0.97 (0.40, 2.34) | 2.07 (0.74, 5.75) |
| Oxygen ≥ 4 L/min | 1.32 (0.99, 1.74) | 4.90 (2.69, 8.93) | 0.44 (0.19, 1.02) | 1.62 (0.47, 5.56) | 4.14 (1.05, 16.3) | 2.55 (1.47, 4.40) | 1.28 (0.80, 2.04) | 1.23 (0.71, 2.15) | 2.86 (1.30, 6.30) | 2.61 (0.93, 7.34) |
| HFNC/NIV | 2.08 (1.49, 2.89) | 9.64 (5.11, 18.2) | 1.43 (0.60, 3.42) | 6.58 (1.92, 22.5) | 2.71 (0.49, 15.0) | 4.84 (2.66, 8.81) | 1.57 (0.89, 2.74) | 1.59 (0.80, 3.13) | 5.70 (2.40, 13.5) | 4.84 (1.59, 14.7) |
| **Corticosteroid use** | 1.31 (1.03, 1.66) | 1.30 (0.88, 1.92) | 0.83 (0.44, 1.56) | 0.67 (0.28, 1.60) | 0.93 (0.36, 1.82) | 1.29 (0.86, 1.94) | 1.32 (0.89, 1.97) | 1.24 (0.78, 1.96) | 1.04 (0.57, 1.89) | 1.49 (0.72, 3.07) |
| **Active trial agent** | 0.85 (0.71, 1.02) | 0.85 (0.64, 1.13) | 0.59 (0.35, 1.00) | 0.76 (0.37, 1.55) | 0.82 (0.37, 1.82) | 0.89 (0.65, 1.20) | 0.76 (0.56, 1.03) | 0.78 (0.54, 1.12) | 1.47 (0.92, 2.36) | 0.85 (0.51, 1.41) |
| **Inflammatory markers** |  |  |  |  |  |  |  |  |  |  |
| CRP (log_2_) | 1.11 (1.03, 1.19) | 1.09 (0.98, 1.21) | 1.13 (0.92, 1.38) | 1.35 (1.00, 1.84) | 1.08 (0.79, 1.48) | 1.14 (1.02, 1.28) | 1.04 (0.93, 1.15) | 1.08 (0.92, 1.26) | 1.21 (1.00, 1.46) | 1.12 (0.92, 1.36) |
| Interleukin-6 (log_2_) | 1.15 (1.09, 1.21) | 1.22 (1.13, 1.32) | 1.11 (0.94, 1.30) | 1.08 (0.89, 1.31) | 1.20 (0.98, 1.46) | 1.19 (1.10, 1.29) | 1.14 (1.04, 1.24) | 1.11 (0.98, 1.24) | 1.19 (1.06, 1.34) | 1.11 (0.97, 1.28) |

Abbreviations: CI, confidence interval; CVD, cardiovascular disease; CKD, chronic kidney disease; CRP, C-reactive protein; HFNO, high-flow nasal oxygen; HR, hazard ratio; N, number; NIV, non-invasive ventilation; VL, viral load.
^a^Age categorized in 10-year bins as age decades.

**Table S5. STROBE checklist.**

|  | **Item** | **Recommendation** | **Page** |
| --- | --- | --- | --- |
| **Title and abstract** | 1 | (a) Indicate the study’s design with a commonly used term in the title or the abstract | 1, 3 |
|  |  | (b) Provide in the abstract an informative and balanced summary of what was done and what was found | 3 |
| **Introduction** | | | |
| Background/rationale | 2 | Explain the scientific background and rationale for the investigation being reported | 4 |
| Objectives | 3 | State specific objectives, including any prespecified hypotheses | 4 |
| **Methods** | | | |
| Study design | 4 | Present key elements of study design early in the paper | 5 |
| Setting | 5 | Describe the setting, locations, and relevant dates, including periods of recruitment, exposure, follow-up, and data collection | 5 |
| Participants | 6 | (a) Give the eligibility criteria, and the sources and methods of selection of participants. Describe methods of follow-up | 5 |
|  |  | (b) For matched studies, give matching criteria and number of exposed and unexposed | N/A |
| Variables | 7 | Clearly define all outcomes, exposures, predictors, potential confounders, and effect modifiers. Give diagnostic criteria, if applicable | 5, 6 |
| Data sources/ measurement | 8 | For each variable of interest, give sources of data and details of methods of assessment (measurement). Describe comparability of assessment methods if there is more than one group | 5, 6 |
| Bias | 9 | Describe any efforts to address potential sources of bias | 5 |
| Study size | 10 | Explain how the study size was arrived at | 5 |
| Quantitative variables | 11 | Explain how quantitative variables were handled in the analyses. If applicable, describe which groupings were chosen and why | 5 |
| Statistical methods | 12 | (a) Describe all statistical methods, including those used to control for confounding | 6 |
|  |  | (b) Describe any methods used to examine subgroups and interactions | 6 |
|  |  | (c) Explain how missing data were addressed | 6 |
|  |  | (d) If applicable, explain how loss to follow-up was addressed | 6 |
|  |  | (e) Describe any sensitivity analyses | 6 |
| **Results** | | | |
| Participants | 13 | (a) Report numbers of individuals at each stage of study—e.g., numbers potentially eligible, examined for eligibility, confirmed eligible, included in the study, completing follow-up, and analyzed | 7 |
|  |  | (b) Give reasons for non-participation at each stage | 7 |
|  |  | (c) Consider use of a flow diagram | 7 |
| Descriptive data | 14 | (a) Give characteristics of study participants (eg demographic, clinical, social) and information on exposures and potential confounders | 7 |
|  |  | (b) Indicate number of participants with missing data for each variable of interest | 7 |
|  |  | (c) Summarize follow-up time (e.g., average and total amount) | 7 |
| Outcome data | 15 | Report numbers of outcome events or summary measures over time | 7 |
| Main results | 16 | (a) Give unadjusted estimates and, if applicable, confounder-adjusted estimates and their precision (eg, 95% confidence interval). Make clear which confounders were adjusted for and why they were included | 7, 8 |
|  |  | (b) Report category boundaries when continuous variables were categorized | 7, 8 |
|  |  | (c) If relevant, consider translating estimates of relative risk into absolute risk for a meaningful time period | N/A |
| Other analyses | 17 | Report other analyses done—e.g., analyses of subgroups and interactions, and sensitivity analyses | 7, 8 |
| **Discussion** | | | |
| Key results | 18 | Summarize key results with reference to study objectives | 9 |
| Limitations | 19 | Discuss limitations of the study, taking into account sources of potential bias or imprecision. Discuss both direction and magnitude of any potential bias | 9 |
| Interpretation | 20 | Give a cautious overall interpretation of results considering objectives, limitations, multiplicity of analyses, results from similar studies, and other relevant evidence | 9, 10 |
| Generalizability | 21 | Discuss the generalizability (external validity) of the study results | 9, 10 |
| **Other information** | | | |
| Funding | 22 | Give the source of funding and the role of the funders for the present study and, if applicable, for the original study on which the present article is based | 11 |

**Table S6. List of all study group members.**

| **Last name** | **First name** | **Degrees** | **Affiliation** |
| --- | --- | --- | --- |
| Sahner | David | M.D. | U.S. National Institute of Allergy and Infectious Diseases incl. Department of Clinical Research |
| Tierney | John | B.Sc.N. | U.S. National Institute of Allergy and Infectious Diseases incl. Department of Clinical Research |
| Vogel | Susan E. | R.N., B.Sc.N. | U.S. National Institute of Allergy and Infectious Diseases incl. Department of Clinical Research |
| Herpin | Betsey R. | M.Sc.N., C.C.R.C., R.N. | U.S. National Institute of Allergy and Infectious Diseases incl. Department of Clinical Research |
| Smolskis | Mary C. | B.Sc.N., M.A | U.S. National Institute of Allergy and Infectious Diseases incl. Department of Clinical Research |
| McKay | Laura A. | M.SC. | U.S. National Institute of Allergy and Infectious Diseases incl. Department of Clinical Research |
| Cahill | Kelly | R.N., M.Sc., C.C.R.C., R.A.C. | U.S. National Institute of Allergy and Infectious Diseases incl. Department of Clinical Research |
| Crew | Page | PharmD., M.P.H., B.C.P.S. | U.S. National Institute of Allergy and Infectious Diseases incl. Department of Clinical Research |
| Sardana | Ratna | B.A. | U.S. National Institute of Allergy and Infectious Diseases incl. Department of Clinical Research |
| Raim | Sharon Segal | M.P.H. | U.S. National Institute of Allergy and Infectious Diseases incl. Department of Clinical Research |
| Hensely | Lisa | Ph.D. | U.S. National Institute of Allergy and Infectious Diseases incl. Department of Clinical Research |
| Lorenzo | Johsua | M.P.H. | U.S. National Institute of Allergy and Infectious Diseases incl. Department of Clinical Research |
| Mock | Rebecca | Ph.D., R.A.C. | U.S. National Institute of Allergy and Infectious Diseases incl. Department of Clinical Research |
| Zuckerman | Judith | B.S.N. | U.S. National Institute of Allergy and Infectious Diseases incl. Department of Clinical Research |
| Atri | Negin | M.P.H. | U.S. National Institute of Allergy and Infectious Diseases incl. Department of Clinical Research |
| Miller | Mark | PharmD., B.C.P.S., R.A.C. | U.S. National Institute of Allergy and Infectious Diseases incl. Department of Clinical Research |
| Vallee | David | PharmD., M.P.H. | U.S. National Institute of Allergy and Infectious Diseases incl. Department of Clinical Research |
| Chung | Lucy | PharmD., C.C.R.P. | U.S. National Institute of Allergy and Infectious Diseases incl. Department of Clinical Research |
| Kang | Nayon | PharmD., M.S. | U.S. National Institute of Allergy and Infectious Diseases incl. Department of Clinical Research |
| Barrett | Kevin | R.N., B.Sc.N. | U.S. National Institute of Allergy and Infectious Diseases incl. Department of Clinical Research |
| Adam | Stacey J. | Ph.D. | Foundation for the National Institutes of Health, The Accelerating COVID-19 Therapeutic Interventions and Vaccines (ACTIV) and Operation Warp Speed |
| Read | Sarah | M.D. | Foundation for the National Institutes of Health, The Accelerating COVID-19 Therapeutic Interventions and Vaccines (ACTIV) and Operation Warp Speed |
| Draghia-Akli | Ruxandra | M.D., Ph.D. | Foundation for the National Institutes of Health, The Accelerating COVID-19 Therapeutic Interventions and Vaccines (ACTIV) and Operation Warp Speed |
| Currier | Judy | M.D. | Foundation for the National Institutes of Health, The Accelerating COVID-19 Therapeutic Interventions and Vaccines (ACTIV) and Operation Warp Speed |
| Hughes | Eric | M.D., Ph.D. | Foundation for the National Institutes of Health, The Accelerating COVID-19 Therapeutic Interventions and Vaccines (ACTIV) and Operation Warp Speed |
| Harrigan | Rachel H. | M.D. | Foundation for the National Institutes of Health, The Accelerating COVID-19 Therapeutic Interventions and Vaccines (ACTIV) and Operation Warp Speed |
| Amos | Laura |  | INSIGHT SDMC, Division of Biostatistics, School of Public Health and School of Statistics, University of Minnesota, Minneapolis, MN, USA |
| Carlsen | Amy | R.N. | INSIGHT SDMC, Division of Biostatistics, School of Public Health and School of Statistics, University of Minnesota, Minneapolis, MN, USA |
| Carter | Anita |  | INSIGHT SDMC, Division of Biostatistics, School of Public Health and School of Statistics, University of Minnesota, Minneapolis, MN, USA |
| Collins | Gary | M.S. | INSIGHT SDMC, Division of Biostatistics, School of Public Health and School of Statistics, University of Minnesota, Minneapolis, MN, USA |
| Davis | Bionca | M.P.H. | INSIGHT SDMC, Division of Biostatistics, School of Public Health and School of Statistics, University of Minnesota, Minneapolis, MN, USA |
| Denning | Eileen | M.P.H. | INSIGHT SDMC, Division of Biostatistics, School of Public Health and School of Statistics, University of Minnesota, Minneapolis, MN, USA |
| DuChene | Alain |  | INSIGHT SDMC, Division of Biostatistics, School of Public Health and School of Statistics, University of Minnesota, Minneapolis, MN, USA |
| Eckroth | Kate | M.P.H. | INSIGHT SDMC, Division of Biostatistics, School of Public Health and School of Statistics, University of Minnesota, Minneapolis, MN, USA |
| Engen | Nicole | M.S. | INSIGHT SDMC, Division of Biostatistics, School of Public Health and School of Statistics, University of Minnesota, Minneapolis, MN, USA |
| Frase | Alex |  | INSIGHT SDMC, Division of Biostatistics, School of Public Health and School of Statistics, University of Minnesota, Minneapolis, MN, USA |
| Gandits | Greg | M.S. | INSIGHT SDMC, Division of Biostatistics, School of Public Health and School of Statistics, University of Minnesota, Minneapolis, MN, USA |
| Grund | Birgit | Ph.D. | INSIGHT SDMC, Division of Biostatistics, School of Public Health and School of Statistics, University of Minnesota, Minneapolis, MN, USA |
| Harrison | Merrie |  | INSIGHT SDMC, Division of Biostatistics, School of Public Health and School of Statistics, University of Minnesota, Minneapolis, MN, USA |
| Hurlbut | Nancy |  | INSIGHT SDMC, Division of Biostatistics, School of Public Health and School of Statistics, University of Minnesota, Minneapolis, MN, USA |
| Kaiser | Payton |  | INSIGHT SDMC, Division of Biostatistics, School of Public Health and School of Statistics, University of Minnesota, Minneapolis, MN, USA |
| Koopmeiners | Joseph | Ph.D. | INSIGHT SDMC, Division of Biostatistics, School of Public Health and School of Statistics, University of Minnesota, Minneapolis, MN, USA |
| Larson | Gregg | M.A. | INSIGHT SDMC, Division of Biostatistics, School of Public Health and School of Statistics, University of Minnesota, Minneapolis, MN, USA |
| Meger | Sue |  | INSIGHT SDMC, Division of Biostatistics, School of Public Health and School of Statistics, University of Minnesota, Minneapolis, MN, USA |
| Mistry | Shweta Sharma | M.S. | INSIGHT SDMC, Division of Biostatistics, School of Public Health and School of Statistics, University of Minnesota, Minneapolis, MN, USA |
| Murray | Thomas | Ph.D. | INSIGHT SDMC, Division of Biostatistics, School of Public Health and School of Statistics, University of Minnesota, Minneapolis, MN, USA |
| Nelson | Ray | R.N. | INSIGHT SDMC, Division of Biostatistics, School of Public Health and School of Statistics, University of Minnesota, Minneapolis, MN, USA |
| Quan | Kien | M.S. | INSIGHT SDMC, Division of Biostatistics, School of Public Health and School of Statistics, University of Minnesota, Minneapolis, MN, USA |
| Quan | Siu Fun |  | INSIGHT SDMC, Division of Biostatistics, School of Public Health and School of Statistics, University of Minnesota, Minneapolis, MN, USA |
| Reilly | Cavan | Ph.D. | INSIGHT SDMC, Division of Biostatistics, School of Public Health and School of Statistics, University of Minnesota, Minneapolis, MN, USA |
| Siegel | Lianne | Ph.D. | INSIGHT SDMC, Division of Biostatistics, School of Public Health and School of Statistics, University of Minnesota, Minneapolis, MN, USA |
| Thompson | Greg |  | INSIGHT SDMC, Division of Biostatistics, School of Public Health and School of Statistics, University of Minnesota, Minneapolis, MN, USA |
| Vock | David | Ph.D. | INSIGHT SDMC, Division of Biostatistics, School of Public Health and School of Statistics, University of Minnesota, Minneapolis, MN, USA |
| Walski | Jamie | M.H.A. | INSIGHT SDMC, Division of Biostatistics, School of Public Health and School of Statistics, University of Minnesota, Minneapolis, MN, USA |
| Gelijns | Annetine C. | Ph.D. | Cardiothoracic Surgical Trials Network (CTSN) International Coordinating Center (ICC). Icahn School of Medicine at Mount Sinai, New York, USA |
| Moskowitz | Alan J. | M.D. | Cardiothoracic Surgical Trials Network (CTSN) International Coordinating Center (ICC). Icahn School of Medicine at Mount Sinai, New York, USA |
| Bagiella | Emilia | Ph.D. | Cardiothoracic Surgical Trials Network (CTSN) International Coordinating Center (ICC). Icahn School of Medicine at Mount Sinai, New York, USA |
| Moquete | Ellen | R.N., B.S.N. | Cardiothoracic Surgical Trials Network (CTSN) International Coordinating Center (ICC). Icahn School of Medicine at Mount Sinai, New York, USA |
| O'Sullivan | Karen | M.P.H. | Cardiothoracic Surgical Trials Network (CTSN) International Coordinating Center (ICC). Icahn School of Medicine at Mount Sinai, New York, USA |
| Marks | Mary E. | R.N., B.S.N. | Cardiothoracic Surgical Trials Network (CTSN) International Coordinating Center (ICC). Icahn School of Medicine at Mount Sinai, New York, USA |
| Accardi | Evan | B.A. | Cardiothoracic Surgical Trials Network (CTSN) International Coordinating Center (ICC). Icahn School of Medicine at Mount Sinai, New York, USA |
| Kinzel | Emily | M.P.H. | Cardiothoracic Surgical Trials Network (CTSN) International Coordinating Center (ICC). Icahn School of Medicine at Mount Sinai, New York, USA |
| Burris | Sarah | M.H.A. | Cardiothoracic Surgical Trials Network (CTSN) International Coordinating Center (ICC). Icahn School of Medicine at Mount Sinai, New York, USA |
| Bedoya | Gabriela | B.S: | Cardiothoracic Surgical Trials Network (CTSN) International Coordinating Center (ICC). Icahn School of Medicine at Mount Sinai, New York, USA |
| Gupta | Lola | M.P.H. | Cardiothoracic Surgical Trials Network (CTSN) International Coordinating Center (ICC). Icahn School of Medicine at Mount Sinai, New York, USA |
| Overbey | Jessica R. | Dr.P.H. | Cardiothoracic Surgical Trials Network (CTSN) International Coordinating Center (ICC). Icahn School of Medicine at Mount Sinai, New York, USA |
| Santos | Milerva | M.P.A. | Cardiothoracic Surgical Trials Network (CTSN) International Coordinating Center (ICC). Icahn School of Medicine at Mount Sinai, New York, USA |
| Gillinov | Marc A. | M.D. | CTSN Steering Committee Chair, Cleveland Clinic Foundation |
| Miller | Marissa A. | D.V.M, M.P.H. | U.S. National Heart Lung and Blood Institute |
| Taddei-Peters | Wendy C. | Ph.D. | U.S. National Heart Lung and Blood Institute |
| Fenton | Kathleen | M.D., M.S. | U.S. National Heart Lung and Blood Institute |
| Sandkovsky | Uriel | M.D., M.S. | Baylor, Scott and White Health |
| Gottlieb | Robert L. | M.D., Ph.D | Baylor, Scott and White Health |
| Mack | Michael | M.D. | Baylor, Scott and White Health |
| Berhe | Mezgebe | M.D., M.P.H. | Baylor, Scott and White Health |
| Haley | Clinton | M.D., M.P.H. | Baylor, Scott and White Health |
| Dishner | Emma | M.D., M.P.H. | Baylor, Scott and White Health |
| Bettacchi | Christopher | M.D. | Baylor, Scott and White Health |
| Golden | Kevin | M.D. | Baylor, Scott and White Health |
| Duhaime | Erin | P.A.-C. | Baylor, Scott and White Health |
| Ryan | Madison | B.S. | Baylor, Scott and White Health |
| Burris | Sarah | M.H.A. | Baylor, Scott and White Health |
| Tallmadge | Catherine | B.A., C.M.A. | Baylor, Scott and White Health |
| Estrada | Lorie | C.C.R.C. | Baylor, Scott and White Health |
| Jones | Felecia | CRC | Baylor, Scott and White Health |
| Villa | Samatha |  | Baylor, Scott and White Health |
| Wang | Samatha | BSN, RN | Baylor, Scott and White Health |
| Robert | Raven | MPH | Baylor, Scott and White Health |
| Coleman | Tanquinisha |  | Baylor, Scott and White Health |
| Clariday | Laura |  | Baylor, Scott and White Health |
| Baker | Rebecca | BSN. RN | Baylor, Scott and White Health |
| Hurutado-Rodriguez | Mariana | BSN, CCRC, CPXP, CMI | Baylor, Scott and White Health |
| Iram | Nazia | CCRC | Baylor, Scott and White Health |
| Fresnedo | Michelle |  | Baylor, Scott and White Health |
| Davis | Allyson |  | Baylor, Scott and White Health |
| Leonard | Kiara |  | Baylor, Scott and White Health |
| Ramierez | Noelia |  | Baylor, Scott and White Health |
| Thammavong | Jon | B.S. | Baylor, Scott and White Health |
| Duque | Krizia |  | Baylor, Scott and White Health |
| Turner | Emma |  | Baylor, Scott and White Health |
| Fisher | Tammy | M.B.A, M.S.N, R.N, C.C.R.C. | Baylor, Scott and White Health |
| Robinson | Dianna | LVN, CCRC | Baylor, Scott and White Health |
| Ransom | Desirae | MS, CCRC | Baylor, Scott and White Health |
| Maldonado | Nicholas | B.A., A.C.R.P.,-CP | Baylor, Scott and White Health |
| Lusk | Erica | CCRP | Baylor, Scott and White Health |
| Killian | Aaron | PharmD. | Baylor, Scott and White Health |
| Palacious | Adriana | PharmD. | Baylor, Scott and White Health |
| Solis | Edilia | BS, CPhT | Baylor, Scott and White Health |
| Jerrow | Janet |  | Baylor, Scott and White Health |
| Watts | Matthew |  | Baylor, Scott and White Health |
| Whitacre | Heather |  | Baylor, Scott and White Health |
| Cothran | Elizabeth |  | Baylor, Scott and White Health |
| Smith | Peter K. | M.D. | Duke University Health System |
| Barkauskas | Christina E. | M.D. | Duke University Health System |
| Vekstein | Andrew M. | M.D. | Duke University Health System |
| Ko | Emily R. | M.D., PhD. | Duke University Health System |
| Dreyer | Grace R. | PA-C | Duke University Health System |
| Stafford | Neil | M.D. | Duke University Health System |
| Brooks | Megan | M.D. | Duke University Health System |
| Der | Tatyana | M.D. | Duke University Health System |
| Witte | Marie | M.D. | Duke University Health System |
| Gamarallage | Ruwan | M.D. | Duke University Health System |
| Franzone | John | M.D. | Duke University Health System |
| Ivey | Noel | M.D. | Duke University Health System |
| Lumsden | Rebecca H. | M.D. | Duke University Health System |
| Mosaly | Nilima | M.D. | Duke University Health System |
| Mourad | Ahmaad | M.D. | Duke University Health System |
| Holland | Thomas L. | M.D. | Duke University Health System |
| Motta | Mary | B.Sc.N., R.N. | Duke University Health System |
| Lane | Kathleen | B.Sc.N., R.N. | Duke University Health System |
| McGowan | Lauren M. | B.Sc., E.M.T.B. | Duke University Health System |
| Stout | Jennifer | B.Sc. | Duke University Health System |
| Aloor | Heather | M.P.H., M.S. | Duke University Health System |
| Bragg | Kennesha M. | M.S. | Duke University Health System |
| Toledo | Barvina | M.A. | Duke University Health System |
| McLendon-Arvik | Beth | PharmD. | Duke University Health System |
| Bussadori | Barbara | R.P.h. | Duke University Health System |
| Hollister | Beth A. | B.Sc.N., R.N | Duke University Health System |
| Griffin | Michelle | M.P.H., E.M.T.P. | Duke University Health System |
| Giangiacomo | Dana M. |  | Duke University Health System |
| Rodriguez | Vicente | M.D. | Lutheran Medical Group |
| Bokhart | Gordon | PharmD. | Lutheran Medical Group |
| Eichman | Sharon M. |  | Lutheran Medical Group |
| Parrino | Patrick E. | M.D., F.A.C.S. | Ochsner Clinic |
| Spindel | Stephen | M.D. | Ochsner Clinic |
| Bansal | Aditya | M.D. | Ochsner Clinic |
| Baumgarten | Katherine | M.D., F.A.C.P., F.I.D.S.A. | Ochsner Clinic |
| Hand | Johnathan | M.D. | Ochsner Clinic |
| Vonderhaar | Derek | M.D. | Ochsner Clinic |
| Nossaman | Bobby | M.D. | Ochsner Clinic |
| Laudun | Sylvia | D.N.P., M.B.A., R.N., C.P.H.Q. | Ochsner Clinic |
| Ames | DeAnna | M.S. | Ochsner Clinic |
| Broussard | Shane |  | Ochsner Clinic |
| Hernandez | Nilmo |  | Ochsner Clinic |
| Isaac | Geralyn | PharmD. | Ochsner Clinic |
| Dinh | Huan | PharmD. | Ochsner Clinic |
| Zheng | Yiling | PharmD. | Ochsner Clinic |
| Tran | Sonny | PharmD. | Ochsner Clinic |
| McDaniel | Hunter |  | Ochsner Clinic |
| Crovetto | Nicolle | M.S. | Ochsner Clinic |
| Perin | Emerson | M.D., PhD. | Texas Heart Institute |
| Costello | Briana | M.D. | Texas Heart Institute |
| Manian | Prasad | M.D. | Texas Heart Institute |
| Sohail | M. Rizwan | M.D. | Texas Heart Institute |
| Postalian | Alexander | M.D. | Texas Heart Institute |
| Hinsu | Punit | PharmD. | Texas Heart Institute |
| Watson | Carolyn |  | Texas Heart Institute |
| Chen | James | RN | Texas Heart Institute |
| Fink | Melyssa |  | Texas Heart Institute |
| Sturgis | Lydia |  | Texas Heart Institute |
| Walker | Kim |  | Texas Heart Institute |
| Mahon | Kim |  | Texas Heart Institute |
| Parenti | Jennifer | RN | Texas Heart Institute |
| Kappenman | Casey | MS | Texas Heart Institute |
| Knight | Aryn |  | Texas Heart Institute |
| Sturek | Jeffrey M. | M.D., Ph.D. | University of Virginia Health Systems |
| Barros | Andrew | M.D., M.S. | University of Virginia Health Systems |
| Enfield | Kyle B. | M.D., F.C.C.M., S.H.E.A. | University of Virginia Health Systems |
| Kadl | Alexandra | M.D. | University of Virginia Health Systems |
| Green | China J. | B.S., C.C.R.C. | University of Virginia Health Systems |
| Simon | Rachel M. | R.N., B.S.N., C.C.R.C. | University of Virginia Health Systems |
| Fox | Ashley | B.S. | University of Virginia Health Systems |
| Thornton | Kara | PharmD., M.Ed., C.C.R.P. | University of Virginia Health Systems |
| Adams | Amy | PharmD., C.C.R.P. | University of Virginia Health Systems |
| Badhwar | Vinay | M.D. | West Virginia University |
| Sharma | Sunil | M.D. | West Virginia University |
| Peppers | Briana | D.O. | West Virginia University |
| McCarthy | Paul | M.D. | West Virginia University |
| Krupica | Troy | M.D. | West Virginia University |
| Sarwari | Arif | M.D., M.S., M.B.A. | West Virginia University |
| Reece | Rebecca | M.D. | West Virginia University |
| Fornaresico | Lisa | Ph.D. | West Virginia University |
| Glaze | Chad | M.S. | West Virginia University |
| Evans | Raquel | B.S.N., R.N. | West Virginia University |
| Di | Fang | R.N., M.S.N. | West Virginia University |
| Carlson | Shawn | M.D., M.S. | West Virginia University |
| Aucremanne | Tanja | B.S.N., R.N. | West Virginia University |
| Tennant | Connie | B.S.N., R.N. | West Virginia University |
| Giblin Sutton | Lisa | Pharm.D. | West Virginia University |
| Buterbaugh | Sabrina | Pharm.D. | West Virginia University |
| Williams | Roger | C.Ph.T. | West Virginia University |
| Bunner | Robin | B.S. | West Virginia University |
| Traverse | Jay H. | M.D. | Minneapolis Heart Institute Foundation |
| Rhame | Frank | M.D. | Minneapolis Heart Institute Foundation |
| Huelster | Joshua | M.D. | Minneapolis Heart Institute Foundation |
| Kethireddy | Rajesh | M.D. | Minneapolis Heart Institute Foundation |
| Davies | Irena | CCRC | Minneapolis Heart Institute Foundation |
| Salamanca | Julianne | MS | Minneapolis Heart Institute Foundation |
| Majeski | Christine | RN, CCRC | Minneapolis Heart Institute Foundation |
| Skelton | Paige | PharmD. | Minneapolis Heart Institute Foundation |
| Zarambo | Maria | PharmD., B.C.O.P | Minneapolis Heart Institute Foundation |
| Sarafolean | Andrea | RN, CCRC | Minneapolis Heart Institute Foundation |
| Bowdish | Michael E. | M.D., M.S. | University of Southern California |
| Borok | Zea | M.B., Ch.B. | University of Southern California |
| Wald-Dickler | Noah | M.D. | University of Southern California |
| Hutcheon | Douglass | M.D. | University of Southern California |
| Towfighi | Amytis | M.D. | University of Southern California |
| Lee | Mary | M.D. | University of Southern California |
| Lewis | Meghan R. | M.D. | University of Southern California |
| Spellberg | Brad | M.D. | University of Southern California |
| Sher | Linda | M.D. | University of Southern California |
| Sharma | Aniket | M.D. | University of Southern California |
| Olds | Anna P. | M.D. | University of Southern California |
| Justino | Chris | P.A.-C. | University of Southern California |
| Loxano | Edward | M.D. | University of Southern California |
| Romero | Chris | C.R.C. | University of Southern California |
| Leong | Janet | C.R.C. | University of Southern California |
| Rodina | Valentina | M.D. | University of Southern California |
| Quesada | Christine | C.R.C. | University of Southern California |
| Hamilton | Luke |  | University of Southern California |
| Escobar | Jose |  | University of Southern California |
| Leshnower | Brad | M.D., F.A.C.S. | Emory University |
| Bender | William | M.D., M.P.H. | Emory University |
| Sharifpour | Milad | M.D., M.S. | Emory University |
| Miller | Jeffrey | M.D. | Emory University |
| Farrington | Woodrow | M.D. | Emory University |
| Baio | Kim T. | R.N., M.S.N. | Emory University |
| McBride | Mary | R.N., B.S.N., M.A.S. | Emory University |
| Fielding | Michele | R.N., B.S.N., C.C.R.C. | Emory University |
| Mathewson | Sonya | R.N., B.S.N., C.C.R.C. | Emory University |
| Porte | Kristina | B.A:, C.C.R.C. | Emory University |
| Maton | Missy | R.N., B.S.N. | Emory University |
| Ponder | Chari | R.N., B.S.N. | Emory University |
| Haley | Elisabeth | R.N., B.S.N., C.C.R.C. | Emory University |
| Spainhour | Christine | R.N., C.C.R.C. | Emory University |
| Rogers | Susan | R.Ph. | Emory University |
| Tyler | Derrick | C.C.R.P. | Emory University |
| Madathil | Ronson J. | M.D. | University of Maryland |
| Rabin | Joseph | M.D. | University of Maryland |
| Levine | Andrea | M.D. | University of Maryland |
| Saharia | Kapil | M.D. | University of Maryland |
| Tabatabai | Ali | M.D. | University of Maryland |
| Lau | Christine | M.D., M.B.A. | University of Maryland |
| Gammie | James S. | M.D. | University of Maryland |
| Peguero | Maya-Loren |  | University of Maryland |
| McKernan | Kimberly |  | University of Maryland |
| Audette | Mathew |  | University of Maryland |
| Fleischmann | Emily |  | University of Maryland |
| Akbari | Kreshta | M.S. | University of Maryland |
| Lee | Myounghee | Ph.D., Pharm. D. | University of Maryland |
| Chi | Andrew | Pharm.D. | University of Maryland |
| Salehi | Hanna | Pharm.D. | University of Maryland |
| Pariser | Alan | Pharm.D. | University of Maryland |
| Nyguyen | Phuong Tran | Pharm.D. | University of Maryland |
| Moore | Jessica |  | University of Maryland |
| Gee | Adrienne |  | University of Maryland |
| Vincent | Shelika |  | University of Maryland |
| Zuckerman | Richard A. | M.D., M.P.H. | Dartmouth-Hitchcock Medical Center |
| Iribarne | Alexander | M.D., M.S. | Dartmouth-Hitchcock Medical Center |
| Metzler | Sara | B.S.N., R.N. | Dartmouth-Hitchcock Medical Center |
| Shipman | Samantha | B.S.N., R.N. | Dartmouth-Hitchcock Medical Center |
| Johnson | Haley |  | Dartmouth-Hitchcock Medical Center |
| Newton | Crystallee | B.A., C.C.R.C. | Dartmouth-Hitchcock Medical Center |
| Parr | Doug | Pharm.D. | Dartmouth-Hitchcock Medical Center |
| Miller | Leslie | M.D. | BayCare Health System |
| Schelle | Beth | R.N. | BayCare Health System |
| McLean | Sherry | R.N. | BayCare Health System |
| Rothbaum | Howard R. | M.D. | BayCare Health System |
| Alvarez | Michael S. | D.O. | BayCare Health System |
| Kalan | Shivam P. | M.D. | BayCare Health System |
| Germann | Heather H. | M.D. | BayCare Health System |
| Hendershot | Jennifer | Pharm.D., B.C.C.C.P. | BayCare Health System |
| Moroney | Karen | R.N. | BayCare Health System |
| Herring | Karen | R.N. | BayCare Health System |
| Cook | Sharri | R.R.T. | BayCare Health System |
| Paul | Pam |  | BayCare Health System |
| Walker-Ignasiak | Rebecca |  | BayCare Health System |
| North | Crystal | M.D. | Prevention and Early Treatment of Acute Lung Injury (PETAL) ICC, Massachusetts General Hospital, Boston, Massachusetts, USA |
| Oldmixon | Cathryn | R.N. | Prevention and Early Treatment of Acute Lung Injury (PETAL) ICC, Massachusetts General Hospital, Boston, Massachusetts, USA |
| Ringwood | Nancy | B.S.N. | Prevention and Early Treatment of Acute Lung Injury (PETAL) ICC, Massachusetts General Hospital, Boston, Massachusetts, USA |
| Muzikansky | Ariela | R.N., B.A./B.S. | Prevention and Early Treatment of Acute Lung Injury (PETAL) ICC, Massachusetts General Hospital, Boston, Massachusetts, USA |
| Morse | Richard | B.A./B.S. | Prevention and Early Treatment of Acute Lung Injury (PETAL) ICC, Massachusetts General Hospital, Boston, Massachusetts, USA |
| Fitzgerald | Laura | B.A./B.S. | Prevention and Early Treatment of Acute Lung Injury (PETAL) ICC, Massachusetts General Hospital, Boston, Massachusetts, USA |
| Morin | Haley D. | B.S.N. | Prevention and Early Treatment of Acute Lung Injury (PETAL) ICC, Massachusetts General Hospital, Boston, Massachusetts, USA |
| Brower | Roy G. | M.D. | PETAL Steering Committee Chair, Johns Hopkins University |
| Reineck | Lora A. | M.D., M.S. | U.S. National Heart Lung and Blood Institute |
| Bienstock, | Karen | PA-C, M.S. | U.S. National Heart Lung and Blood Institute |
| Steingrub | Jay H. | M.D. | ALIGNE Site Coordinating Center (SCC) Lead Investigators, Baystate Medical Center |
| Hou | Peter K. | M.D. | ALIGNE Site Coordinating Center (SCC) Lead Investigators, Brigham and Women's Hospital |
| Steingrub | Jay S. | M.D. | Baystate Medical Center |
| Tidswell | Mark A. | M.D. | Baystate Medical Center |
| Kozikowski | Lori-Ann | R.N., B.S.N., C.C.R.N. | Baystate Medical Center |
| Kardos | Cynthia | R.N., B.S.N., C.C.R.N. | Baystate Medical Center |
| DeSouza | Leslie |  | Baystate Medical Center |
| Romain | Sarah | R.N., B.S.N. | Baystate Medical Center |
| Thornton-Thompson | Sherell |  | Baystate Medical Center |
| Talmor | Daniel | M.D. | Boston SCC Lead Investigators, Beth Israel Deaconess Medical Center |
| Shapiro | Nathan | M.D. | Boston SCC Lead Investigators, Beth Israel Deaconess Medical Center |
| Andromidas, | Konstantinos |  | Beth Israel Deaconess Medical Center |
| Banner-Goodspeed, | Valerie | M.P.H. | Beth Israel Deaconess Medical Center |
| Bolstad | Michael |  | Beth Israel Deaconess Medical Center |
| Boyle, | Katherine L. | M.D. | Beth Israel Deaconess Medical Center |
| Cabrera | Payton |  | Beth Israel Deaconess Medical Center |
| deVilla, | Arnaldo | R.N., M.P.H. | Beth Israel Deaconess Medical Center |
| Ellis, | Joshua C. | M.D. | Beth Israel Deaconess Medical Center |
| Grafals, | Ana |  | Beth Israel Deaconess Medical Center |
| Hayes | Sharon | R.N. | Beth Israel Deaconess Medical Center |
| Higgins | Conor |  | Beth Israel Deaconess Medical Center |
| Kurt | Lisa |  | Beth Israel Deaconess Medical Center |
| Kurtzman, | Nicholas | M.D. | Beth Israel Deaconess Medical Center |
| Redman, | Kimberly | R.N., B.S.N. | Beth Israel Deaconess Medical Center |
| Rosseto | Elinita |  | Beth Israel Deaconess Medical Center |
| Scaffidi | Douglas |  | Beth Israel Deaconess Medical Center |
| Shapiro, | Nathan | M.D., M.P.H. | Beth Israel Deaconess Medical Center |
| Filbin, | Michael R. | M.D., M.Sc. | Massachusetts General Hospital |
| Hibbert, | Kathryn A. | M.D. | Massachusetts General Hospital |
| Parry, | Blair | C.C.R.C., B.A. | Massachusetts General Hospital |
| Margolin, | Justin | B.S. | Massachusetts General Hospital |
| Hillis, | Brooklynn | B.S.N, R.N. | Massachusetts General Hospital |
| Hamer | Rhonda |  | Massachusetts General Hospital |
| Brait | Kelsey | B.B.A., B.Sc. | Massachusetts General Hospital |
| Beakes | Caroline | B.S. | Massachusetts General Hospital |
| McKaig | Brenna | B.S. | Massachusetts General Hospital |
| Kugener | Eleonore | B.A. | Massachusetts General Hospital |
| Jones | Alan E. | M.D. | University of Mississippi |
| Galbraith | James | M.D. | University of Mississippi |
| Nandi | Utsav | M.D. | University of Mississippi |
| Peacock | Rebekah | R.N. | University of Mississippi |
| Hendey | Gregory | M.D. | California SCC Lead Investigators, David Geffen School of Medicine at UCLA |
| Kangelaris | Kirsten | M.D., M.A.S. | University of California San Francisco |
| Ashktorab | Kimia | B.A. | University of California San Francisco |
| Gropper | Rachel | B.S. | University of California San Francisco |
| Agrawal | Anika | B.S. | University of California San Francisco |
| Yee | Kimberley J. | B.Sc. | University of California San Francisco |
| Jauregui | Alejandra E. | B.A. | University of California San Francisco |
| Zhuo | Hanjing | M.P.H. | University of California San Francisco |
| Almasri | Eyad | M.D. | University of California Fresno |
| Fayed | Mohamed | M.D. | University of California Fresno |
| Hubel | Kinsley A. | M.D. | University of California Fresno |
| Hughes | Alyssa R. | B.S. | University of California Fresno |
| Garcia | Rebekah L. | C.C.R.P. | University of California Fresno |
| Lim | George W. | M.D. | Ronald Reagan UCLA Medical Center |
| Chang | Steven Y. | M.D. | Ronald Reagan UCLA Medical Center |
| Hendey | Gregory | M.D. | Ronald Reagan UCLA Medical Center |
| Lin | Michael Y. | M.D. | Ronald Reagan UCLA Medical Center |
| Vargas | Julia | B.S. | Ronald Reagan UCLA Medical Center |
| Sihota | Hena | B.S. | Ronald Reagan UCLA Medical Center |
| Beutler | Rebecca | M.S. | Ronald Reagan UCLA Medical Center |
| Agarwal | Trisha |  | Ronald Reagan UCLA Medical Center |
| Wilson, | Jennifer G. | M.D., M.S. | Stanford University |
| Vojnik, | Rosemary | B.S. | Stanford University |
| Perez, | Cynthia | B.S. | Stanford University |
| McDowell, | Jordan H. | M.S. | Stanford University |
| Roque | Jonasel | B.S. | Stanford University |
| Wang | Henry | M.D., M.S. | University of Texas Health Science Center |
| Huebinger | Ryan M. | M.D. | University of Texas Health Science Center |
| Patel | Bela | M.D. | University of Texas Health Science Center |
| Vidales | Elizabeth | M.P.H., B.M.S. | University of Texas Health Science Center |
| Albertson | Timothy | M.D. | University of California Davis Health |
| Hardy | Erin | B.A./B.S. | University of California Davis Health |
| Harper | Richart | M.D. | University of California Davis Health |
| Moss | Marc A. | M.D. | Colorado SCC Lead Investigators, University of Colorado Hospital |
| Baduashvili | Amiran | M.D. | University of Colorado Hospital |
| Chauhan | Lakshmi | M.D. | University of Colorado Hospital |
| Douin | David J. | M.D. | University of Colorado Hospital |
| Martinez | Flora | R.N. | University of Colorado Hospital |
| Finck | Lani L. | M.P.H. | University of Colorado Hospital |
| Bastman | Jill | R.N. | University of Colorado Hospital |
| Howell | Michelle | R.N. | University of Colorado Hospital |
| Higgins | Carrie | R.N. | University of Colorado Hospital |
| McKeehan | Jeffrey | M.Sc.N. | University of Colorado Hospital |
| Finigan | Jay | M.D. | National Jewish Health/ St. Joseph Hospital |
| Stubenrauch | Peter | M.D. | National Jewish Health/ St. Joseph Hospital |
| Janssen | William J. | M.D. | National Jewish Health/ St. Joseph Hospital |
| Griesmer | Christine | R.N., M.P.H. | National Jewish Health/ St. Joseph Hospital |
| VerBurg | Olivia | B.A. | National Jewish Health/ St. Joseph Hospital |
| Hyzy | Robert C. | M.D. | Michigan SCC Lead Investigators, University of Michigan |
| Park | Pauline K. | M.D. | Michigan SCC Lead Investigators, University of Michigan |
| Nelson, | Kristine | R.N. | University of Michigan |
| McSparron, | Jake I. | M.D. | University of Michigan |
| Co, | Ivan N. | M.D. | University of Michigan |
| Wang, | Bonnie R. | M.D. | University of Michigan |
| Jimenez, | Jose | M.D. | University of Michigan |
| Olbrich | Norman |  | University of Michigan |
| McDonough | Kelli |  | University of Michigan |
| Jia | Shijing | M.D. | University of Michigan |
| Hanna | Sinan |  | University of Michigan |
| Gong | Michelle N. | M.D., M.S. | Montefiore-Sinai SCC Lead Investigators: Montefiore Medical Center |
| Richardson | Lynne D. | M.D. | Mount Sinai Hospital |
| Nair | Rahul | M.D. | Montefiore Medical Center Moses |
| Lopez | Brenda | M.D. | Montefiore Medical Center Moses |
| Amosu | Omowunmi | M.S. | Montefiore Medical Center Moses |
| Offor | Obiageli | M.D. | Montefiore Medical Center Moses |
| Tzehaie | Hiwet | B.S. | Montefiore Medical Center Moses |
| Nkemdirim | William | M.D. | Montefiore Medical Center Moses |
| Boujid | Sabah | B.S. | Montefiore Medical Center Moses |
| Mosier | Jarrod M. | M.D. | Banner University Medical Center Tucson |
| Hypes | Cameron | M.D. | Banner University Medical Center Tucson |
| Campbell | Elizabeth Salvagio | Ph.D. | Banner University Medical Center Tucson |
| Bixby | Billie | M.D. | Banner University Medical Center Tucson |
| Gilson | Boris | B.A./B.S. | Banner University Medical Center Tucson |
| Lopez | Anitza | B.S. | Banner University Medical Center Tucson |
| Bime | Christian | M.D. | Banner University Medical Center Tucson |
| Parthasarathy | Sairam | M.D. | Banner University Medical Center Tucson |
| Cano | Ariana M. | B.A., B.S. | Banner University Medical Center Tucson |
| Hite | R. Duncan | M.D. | Ohio SCC Lead Investigators, University of Cincinnati |
| Terndrup | Thomas E. | M.D. | Ohio State University |
| Wiedemann | Herbert P. | M.D., M.B.A. | Cleveland Clinic Foundation |
| Hudock | Kristin | M.D. | University of Cincinnati |
| Tanzeem | Hammad | M.D. | University of Cincinnati |
| More | Harshada | M.D. | University of Cincinnati |
| Martinkovic | Jamie | C.N.P. | University of Cincinnati |
| Sellers | Susan | R.N., B.S.N., C.C.R.P. | University of Cincinnati |
| Houston | Judy | PharmD. | University of Cincinnati |
| Burns | Mary | PharmD. | University of Cincinnati |
| Kiran | Simra | M.D. | University of Cincinnati |
| Roads | Tammy | C.C.R.P. | University of Cincinnati |
| Kennedy | Sarah | C.N.P. | University of Cincinnati |
| Duggal | Abhijit | M.D. | Cleveland Clinic Foundation, Cleveland Clinic Fairview Hospital |
| Thiruchelvam | Nirosshan | M.D. | Cleveland Clinic Foundation, Cleveland Clinic Fairview Hospital |
| Ashok | Kiran | B.S. | Cleveland Clinic Foundation, Cleveland Clinic Fairview Hospital |
| King | Alexander H. | M.S. | Cleveland Clinic Foundation, Cleveland Clinic Fairview Hospital |
| Mehkri | Omar | M.D. | Cleveland Clinic Foundation, Cleveland Clinic Fairview Hospital |
| Dugar | Siddharth | M.D. | Cleveland Clinic Foundation, Cleveland Clinic Fairview Hospital |
| Sahoo | Debasis | M.D. | Cleveland Clinic Foundation, Cleveland Clinic Fairview Hospital |
| Yealy | Donald M. | M.D. | University of Pittsburgh Medical Center |
| Angus | Derek C. | M.D. | University of Pittsburgh Medical Center |
| Weissman | Alexandra J. | M.D. | University of Pittsburgh Medical Center |
| Vita | Tina M. | R.N. | University of Pittsburgh Medical Center |
| Berryman | Emily | B.S., B.A. | University of Pittsburgh Medical Center |
| Hough | Catherine L. | M.D. | Pacific Northwest SCC Lead Investigators, Oregon Health and Science University |
| Khan | Akram | M.D. | Oregon Health and Science University |
| Krol | Olivia F. |  | Oregon Health and Science University |
| Mills | Emmanuel | M.D. | Oregon Health and Science University |
| Kinjal | Mistry |  | Oregon Health and Science University |
| Briceno | Genesis |  | Oregon Health and Science University |
| Reddy | Raju | M.D. | Oregon Health and Science University |
| Hubel | Kinsley | M.D. | Oregon Health and Science University |
| Jouzestani | Milad K. |  | Oregon Health and Science University |
| McDougal | Madeline | B.A., B.S. | Oregon Health and Science University |
| Deshmukh | Rupali |  | Oregon Health and Science University |
| Johnston | Nicholas J. | M.D. | Harborview Medical Center, University of Washington Medical Center |
| Robinson | Bryce H. | M.D. | Harborview Medical Center, University of Washington Medical Center |
| Gundel | Staphanie J. | R.D. | Harborview Medical Center, University of Washington Medical Center |
| Katsandres | Sarah C. | B.S. | Harborview Medical Center, University of Washington Medical Center |
| Chen | Peter | M.D. | Cedars-Sinai Medical Center |
| Torbati | Sam S. | M.D. | Cedars-Sinai Medical Center |
| Parimon | Tanyalak | M.D. | Cedars-Sinai Medical Center |
| Caudill | Antonina | M.P.H., C.P.H. | Cedars-Sinai Medical Center |
| Mattison | Brittany |  | Cedars-Sinai Medical Center |
| Jackman | Susan E. | B.S.N., M.S. | Cedars-Sinai Medical Center |
| Chen | Po-En | B.S.N. | Cedars-Sinai Medical Center |
| Bayoumi | Emad | M.D. | Cedars-Sinai Medical Center |
| Ojukwu | Cristabelle | B.S. | Cedars-Sinai Medical Center |
| Fine | Devin | B.S. | Cedars-Sinai Medical Center |
| Weissberg | Gwendolyn | B.S. | Cedars-Sinai Medical Center |
| Isip | Katherine | B.S. | Cedars-Sinai Medical Center |
| Choi-Kuaea | Yunhee | M.S.W. | Cedars-Sinai Medical Center |
| Mehdikhani | Shaunt | M.S. | Cedars-Sinai Medical Center |
| Dar | Tahir B. | Ph.D. | Cedars-Sinai Medical Center |
| Augustin | Nsole Biteghe Fleury | Ph.D. | Cedars-Sinai Medical Center |
| Tran | Dana | B.S. | Cedars-Sinai Medical Center |
| Dukov | Jennifer Emilow | B.S. | Cedars-Sinai Medical Center |
| Matusov | Yuri | M.D. | Cedars-Sinai Medical Center |
| Choe | June | M.D. | Cedars-Sinai Medical Center |
| Hindoyan | Niree A. | B.S. | Cedars-Sinai Medical Center |
| Wynter | Timothy | B.S. | Cedars-Sinai Medical Center |
| Pascual | Ethan | M.A. | Cedars-Sinai Medical Center |
| Clapham | Gregg J. | M.A: | Cedars-Sinai Medical Center |
| Herrera | Lisa |  | Cedars-Sinai Medical Center |
| Caudill | Antonia | M.P.H., C.P.H. | Cedars-Sinai Medical Center |
| O’Mahony | D. Shane | M.D. | Swedish Hospital First Hill |
| Nyatsatsang | Sonam T. | M.D. | Swedish Hospital First Hill |
| Wilson | David M. | M.D. | Swedish Hospital First Hill |
| Wallick | Julie A. | B.A./B.S. | Swedish Hospital First Hill |
| Duven | Alexandria M. | R.N. | Swedish Hospital First Hill |
| Fletcher | Dakota D. | B.S. | Swedish Hospital First Hill |
| Miller | Chadwick | M.D. | Wake Forest Baptist Health |
| Files | D. Clark | M.D. | Wake Forest Baptist Health |
| Gibbs | Kevin W. | M.D. | Wake Forest Baptist Health |
| Flores, | Lori S. | D.N.P. | Wake Forest Baptist Health |
| LaRose | Mary E. | R.N., B.S.N. | Wake Forest Baptist Health |
| Landreth | Leigha D. | R.N., B.S.N. | Wake Forest Baptist Health |
| Palacios | D. Rafael | B.S.C.R. | Wake Forest Baptist Health |
| Parks | Lisa | R.N. | Wake Forest Baptist Health |
| Hicks | Madeline | B.A. | Wake Forest Baptist Health |
| Goodwin | Andrew J. | M.D. | Medical Center of South Carolina |
| Kilb | Edward F. | M.D. | Medical Center of South Carolina |
| Lematty | Caitlan T. | B.S. | Medical Center of South Carolina |
| Patti | Kerilyn |  | Medical Center of South Carolina |
| Grady | Abigail | B.S. | Medical Center of South Carolina |
| Rasberry | April | B.S. | Medical Center of South Carolina |
| Morris | Peter E. | M.D. | University of Kentucky |
| Sturgill | Jamie L. | Ph.D. | University of Kentucky |
| Cassity | Evan P. | M.S. | University of Kentucky |
| Dhar | Sanjay | M.D. | University of Kentucky |
| Montgomery-Yates | Ashley A. | M.D. | University of Kentucky |
| Pasha | Sarah N. | M.D. | University of Kentucky |
| Mayer | Kirby P. | Ph.D. | University of Kentucky |
| Bissel | Brittany | Pharm.D., Ph.D. | University of Kentucky |
| Trott | Terren | M.D. | University of Kentucky |
| Rehman | Shahnaz | M.D. | University of Kentucky |
| de Wit | Marjolein | M.D. | Virginia Commonwealth University |
| Mason | Jessica | M.P.H. | Virginia Commonwealth University |
| Bledsoe | Joseph | M.D. | Intermountain Medical Center |
| Knowlton | Kirk U. | M.D. | Intermountain Medical Center |
| Brown | Samuel | M.D. | Intermountain Medical Center |
| Lanspa | Michael | M.D. | Intermountain Medical Center |
| Leither | Lindsey | M.D. | Intermountain Medical Center |
| Pelton | Ithan | M.D. | Intermountain Medical Center |
| Armbruster | Brent P. | B.S. | Intermountain Medical Center |
| Montgomery | Quinn | B.S., A.E.M.T. | Intermountain Medical Center |
| Kumar | Naresh | M.P.H., C.C.R.P. | Intermountain Medical Center |
| Fergus | Melissa | B.S. | Intermountain Medical Center |
| Imel | Karah | A.S., C.C.R.P. | Intermountain Medical Center |
| Palmer | Ghazal | PharmD. | Intermountain Medical Center |
| Webb | Brandon | M.D. | Intermountain Medical Center |
| Klippel | Carolyn | B.S. | Intermountain Medical Center |
| Jensen | Hannah | B.S. | Intermountain Medical Center |
| Duckworth | Sarah |  | Intermountain Medical Center |
| Gray | Andrew | B.S. | Intermountain Medical Center |
| Burke | Tyler | B.S. | Intermountain Medical Center |
| Knox | Dan | M.D. | Intermountain Medical Center |
| Lumpkin | Jenna | B.S. | Intermountain Medical Center |
| Aston | Valerie T. | M.B.A., R.R.T., C.C.R.P. | Intermountain Medical Center |
| Applegate | Darrin | B.S. | Intermountain Medical Center |
| Serezlic | Erna | B.S. | Intermountain Medical Center |
| Brown | Katie | B.S., R.N. | Intermountain Medical Center |
| Merril | Mardee | B.S., C.C.R.P. | Intermountain Medical Center |
| Harris | Estelle S. | M.D. | University of Utah |
| Middleton | Elizabeth A. | M.D. | University of Utah |
| Barrios | Macy A.G. | B.S. | University of Utah |
| Greer | Jorden | B.S. | University of Utah |
| Schmidt | Amber D. | B.S | University of Utah |
| Webb | Melissa K. | Pharm.D. | University of Utah |
| Paine | Roert | M.D. | University of Utah |
| Callahan | Sean J. | M.D. | University of Utah |
| Waddoups | Lindsey J. | M.S. | University of Utah |
| Yamane | Misty B. | B.S. | University of Utah |
| Self | Wesley H. | M.D., M.P.H. | Vanderbilt SCC Lead Investigators, Vanderbilt University Medical Center |
| Rice | Todd W. | M.D., M.S.C.I. | Vanderbilt SCC Lead Investigators, Vanderbilt University Medical Center |
| Casey | Jonathan D. | M.D., M.S.C.I. | Vanderbilt University Medical Center |
| Johnson | Jakea | M.P.H. | Vanderbilt University Medical Center |
| Gray | Christopher | R.N. | Vanderbilt University Medical Center |
| Hays | Margaret | R.N. | Vanderbilt University Medical Center |
| Roth | Megan | R.N. | Vanderbilt University Medical Center |
|  |  |  | Divison of Clinical Research, NIAID, NIH, ICC |
| Menon | Vidya | M.D, F.A.C.P. | Lincoln Medical Center |
| Kasubhai | Moiz | M.D. | Lincoln Medical Center |
| Pillai | Anjana | M.D. | Lincoln Medical Center |
| Daniel | Jean | M.D., M.A.C.P. | Lincoln Medical Center |
| Sittler | Daniel | M.D. | Lincoln Medical Center |
| Kanna | Balavenkatesh | M.D., M.P.H., F.A.C.P. | Lincoln Medical Center |
| Jilani | Nargis | M.D. | Lincoln Medical Center |
| Amaro | Francisco | R.N., F.N.P.,-BC. | Lincoln Medical Center |
| Santana | Jessica | B.A. | Lincoln Medical Center |
| Lyakovestsky | Aleksandr | PharmD., B.C.P.S. | Lincoln Medical Center |
| Madhoun | Issa | PharmD. | Lincoln Medical Center |
| Desroches | Louis Marie | R.P.H. | Lincoln Medical Center |
| Amadon | Nicole | PharmD., B.C.G.P. | Lincoln Medical Center |
| Bahr | Alaa | PharmD., B.C.P.S. | Lincoln Medical Center |
| Ezzat | Imaan | PharmD., B.H.S.A., M.A. | Lincoln Medical Center |
| Guerrero | Maryanne |  | Lincoln Medical Center |
| Padilla | Joane |  | Lincoln Medical Center |
| Fullmer | Jessie |  | Lincoln Medical Center |
| Singh | Inderpreet |  | Lincoln Medical Center |
| Shah | Syed Hamad Ali |  | Lincoln Medical Center |
| Narang | Rajeev | M.D. | CHRISTUS Spohn Shoreline Hospital |
| Mock | Polly | R.N., C.C.R.C., C.H.R.C. | CHRISTUS Spohn Shoreline Hospital |
| Shadle | Melissa | R.N., B.S.N., O.C.N., C.C.R.C. | CHRISTUS Spohn Shoreline Hospital |
| Hernandez | Brenda | R.N. | CHRISTUS Spohn Shoreline Hospital |
| Welch | Kevin | PharmD. | CHRISTUS Spohn Shoreline Hospital |
| Payne | Andrea | PharmD. | CHRISTUS Spohn Shoreline Hospital |
| Ertl | Gabriela | PharmD. | CHRISTUS Spohn Shoreline Hospital |
| Canario | Daniel | M.D. | Hendrick Medical Center |
| Barrientos | Isabel | M.S.N., A.R.P.N. | Hendrick Medical Center |
| Goss | Danielle | M.P.H., M.H.A. | Hendrick Medical Center |
| DeVries | Mattie | RPh., PharmD. | Hendrick Medical Center |
| Folowosele | Ibidolapo | RPh. | Hendrick Medical Center |
| Garner | Dorothy | M.D. | Carilion Roanoke Memorial Hospital |
| Gomez | Mariana | M.D. | Carilion Roanoke Memorial Hospital |
| Price | Justin | M.D. | Carilion Roanoke Memorial Hospital |
| Bansal | Ekta | M.D. | Carilion Roanoke Memorial Hospital |
| Wong | Jim | M.D. | Carilion Roanoke Memorial Hospital |
| Faulhaber | Jason | M.D. | Carilion Roanoke Memorial Hospital |
| Fazili | Tasaduq | M.D. | Carilion Roanoke Memorial Hospital |
| Yeary | Brian | M.D. | Carilion Roanoke Memorial Hospital |
| Ndolo | Ruth | R.N. | Carilion Roanoke Memorial Hospital |
| Bryant | Christina | R.N. | Carilion Roanoke Memorial Hospital |
| Smigeil | Bridgette | PharmD. | Carilion Roanoke Memorial Hospital |
| Robinson | Philip | M.D. | Hoag Memorial Hospital Presbyterian |
| Najjar | Rana | M.S.H.C.A., C.R.C. | Hoag Memorial Hospital Presbyterian |
| Jones | Patrice | C.R.C. | Hoag Memorial Hospital Presbyterian |
| Nguyen | Julie | R.R.T., C.R.C. | Hoag Memorial Hospital Presbyterian |
| Chin | Christina | PharmD. | Hoag Memorial Hospital Presbyterian |
| Taha | Hassan | M.D. | Cotton O’Neil Clinical Research Center |
| Najm | Salah | M.D., M.B.A. | Cotton O’Neil Clinical Research Center |
| Smith | Christopher | PharmD. | Cotton O’Neil Clinical Research Center |
| Moore | Jason | PharmD. | Cotton O’Neil Clinical Research Center |
| Nassar | Talal | PharmD. | Cotton O’Neil Clinical Research Center |
| Gallinger | Nick | PharmD. | Cotton O’Neil Clinical Research Center |
| Christian | Amy | R.N., C.C.R.C. | Cotton O’Neil Clinical Research Center |
| Mauer | D’Amber | R.N., B.S.N. | Cotton O’Neil Clinical Research Center |
| Phipps | Ashley | R.N., B.S.N. | Cotton O’Neil Clinical Research Center |
| Waters, | Michael | M.D. | Velocity Chula Vista |
| Zepeda | Karla | N.P. | Velocity Chula Vista |
| Coslet | Jordan | P.A. | Velocity Chula Vista |
| Landazuri | Rosalynn | B.S., C.R.C. | Velocity Chula Vista |
| Pineda | Jacob | C.R.C. | Velocity Chula Vista |
| Uribe | Nicole | RPh. | Velocity Chula Vista |
| Garcia | Jose Ruiz | CPhT. | Velocity Chula Vista |
| Barbabosa | Cecilia | R.N. | Velocity Chula Vista |
| Sandler | Kaitlyn | B.S.N. | Velocity Chula Vista |
| Overcash | J. Scott | M.D. | Velocity San Diego |
| Marquez | Adrienna |  | Velocity San Diego |
| Chu | Hanh | M.S.N., NP-C. | Velocity San Diego |
| Lee | Kia | M.S.N., A.N.P.-B.C. | Velocity San Diego |
| Quillin | Kimberly | B.S.N., R.N. | Velocity San Diego |
| Garcia | Andrea | M.S.N., R.N. | Velocity San Diego |
| Lew | Pauline | PharmD. | Velocity San Diego |
| Rogers | Ralph | M.D. | Rhode Island Hospital; The Miriam Hospital |
| Shehadeh | Fadi | M.Sc. | Rhode Island Hospital; The Miriam Hospital |
| Mylona | Evangelia K. | M.Sc. | Rhode Island Hospital; The Miriam Hospital |
| Kaczynski | Matthew | B.Sc. | Rhode Island Hospital; The Miriam Hospital |
| Tran | Quynh-Lam | B.Sc. | Rhode Island Hospital; The Miriam Hospital |
| Benitez | Gregorio | M.P.H. | Rhode Island Hospital; The Miriam Hospital |
| Mishra | Biswajit | Ph.D. | Rhode Island Hospital; The Miriam Hospital |
| Felix | Lewis Oscar | Ph.D. | Rhode Island Hospital; The Miriam Hospital |
| Vafea | Maria Tsikala | M.D. | Rhode Island Hospital; The Miriam Hospital |
| Atalla | Eleftheria | M.D. | Rhode Island Hospital; The Miriam Hospital |
| Davies | Robin | B.S.N., B.A., R.N. | Rhode Island Hospital; The Miriam Hospital |
| Hedili | Salma | C.P.T. | Rhode Island Hospital; The Miriam Hospital |
| Monkeberg | Maria Andrea | M.S., R.Ph., B.C.O.P. | Rhode Island Hospital |
| Tabler | Sandra | R.Ph., B.C.O.P. | Rhode Island Hospital |
| Harrington | Britt | Pharm.D. | The Miriam Hospital |
| Meegada | Sreenath | M.D., | Christus Good Shepard |
| Koripalli | VenkataSandeep | M.D. | Christus Good Shepard |
| Muddana | Prithvi | M.D. | Christus Good Shepard |
| Jain | Lakshay | M.D. | Christus Good Shepard |
| Undavalli | Chaitanya | M.D. | Christus Good Shepard |
| Kavya | Parasa | M.D. | Christus Good Shepard |
| Ibiwoye | Mofoluwaso | M.D. | Christus Good Shepard |
| Akilo | Hameed | M.D. | Christus Good Shepard |
| Lovette, | Bryce D. | PharmD. | Christus Good Shepard |
| Wylie, | Jamie-Crystal | MHA, FACHE, | Christus Good Shepard |
| Smith, | Diana M. | BS | Christus Good Shepard |
| Poon | Kenneth | M.D., F.A.C.P. | Memorial Health Care System |
| Eckardt, | Paula | M.D., F.A.C.P., F.I.D.S.A., A.A.H.I.V.S. | Memorial Health Care System |
| Heysu | Rubio-Gomez, | M.D., F.A.C.P., F.I.D.S.A. | Memorial Health Care System |
| Sundararaman, | Nithya | M.A., M.S., M.B.A. | Memorial Health Care System |
| Alaby, | Doris | B.S.N., R.N. | Memorial Health Care System |
| Sareli | Candice | M.D. | Memorial Health Care System |
| Davey | Victoria J. | PhD | INSIGHT Washington ICC, Veterans Affairs (VA) Medical Center, Washington, DC |
| Kan | Virginia L | M.D. | INSIGHT Washington ICC, Veterans Affairs (VA) Medical Center, Washington, DC |
| Sánchez | Adriana | M.S. | INSIGHT Washington ICC, Veterans Affairs (VA) Medical Center, Washington, DC |
| Popielski | Laura | M.P.H. | INSIGHT Washington ICC, Veterans Affairs (VA) Medical Center, Washington, DC |
| Kambo | Amy | M.P.H. | INSIGHT Washington ICC, Veterans Affairs (VA) Medical Center, Washington, DC |
| Viens | Kimberley | B.S., C.C.R.P. | INSIGHT Washington ICC, Veterans Affairs (VA) Medical Center, Washington, DC |
| Turner | Melissa | M.S.W. | INSIGHT Washington ICC, Veterans Affairs (VA) Medical Center, Washington, DC |
| Vjecha | Michael J. | M.D. | INSIGHT Washington ICC, Veterans Affairs (VA) Medical Center, Washington, DC |
| Weintrob | Amy | M.D. | INSIGHT Washington ICC, Veterans Affairs (VA) Medical Center, Washington, DC |
| Brar | Indira | M.D. | Henry Ford Health System |
| Markowitz | Norman | M.D. | Henry Ford Health System |
| Pastor | Erika | R.N | Henry Ford Health System |
| Corpuz | Roweena | R.N | Henry Ford Health System |
| Alangaden | George | M.D. | Henry Ford Health System |
| McKinnon | John | M.D. | Henry Ford Health System |
| Ramesh | Mayur | M.D. | Henry Ford Health System |
| Herc | Erica | M.D. | Henry Ford Health System |
| Yared | Nicholas | M.D. | Henry Ford Health System |
| Abreu Lanfranco | Odaliz | M.D. | Henry Ford Health System |
| Rivers | Emanuel | M.D. | Henry Ford Health System |
| Swiderek | Jennifer | M.D. | Henry Ford Health System |
| Hodari Gupta | Ariella | M.D. | Henry Ford Health System |
| Pabla | Pardeep | Pharm D | Henry Ford Health System |
| Eliya | Sonia | Pharm D | Henry Ford Health System |
| Jazrawi | Jehan | RpH | Henry Ford Health System |
| Delor | Jeremy | Pharm D | Henry Ford Health System |
| Desai | Mona | Pharm D | Henry Ford Health System |
| Cook | Aaron |  | Henry Ford Health System |
| Kathrina Jaehne | Anja |  | Henry Ford Health System |
| Kaur Gill | Jasreen |  | Henry Ford Health System |
| Renaud | Sheri |  | Henry Ford Health System |
| Sarveswaran | Siva |  | Henry Ford Health System |
| Gardner | Edward | M.D. | Public Health Institute at Denver Health |
| Scott | James | RN | Public Health Institute at Denver Health |
| Bianchini | Monica | Pharm D | Public Health Institute at Denver Health |
| Melvin | Casey | Pharm D | Public Health Institute at Denver Health |
| Kim | Gina | Pharm D | Public Health Institute at Denver Health |
| Wyles | David | M.D. | Public Health Institute at Denver Health |
| Kamis | Kevin |  | Public Health Institute at Denver Health |
| Miller | Rachel |  | Public Health Institute at Denver Health |
| Douglas | Ivor | MD | Public Health Institute at Denver Health |
| Haukoos | Jason |  | Public Health Institute at Denver Health |
| Hicks | Carrie |  | Public Health Institute at Denver Health |
| Lazarte | Susana | MD | Parkland Health and Hospital Systems |
| Marines-Price | Rubria | Ph.D., D.N.P., A.P.R.N. | Parkland Health and Hospital Systems |
| Osuji | Alice | R.N., B.S.N., M.S.N. | Parkland Health and Hospital Systems; University of Texas Southwestern Medical Center |
| Agbor | Barbine Tchamba Agbor | M.D. | Parkland Health and Hospital Systems |
| Petersen | Tianna | M.Sc., M.S.N. | Parkland Health and Hospital Systems; University of Texas Southwestern Medical Center |
| Kamel | Dena | B.S. | Parkland Health and Hospital Systems; University of Texas Southwestern Medical Center |
| Hansen | Laura | M.A. | Parkland Health and Hospital Systems; University of Texas Southwestern Medical Center |
| Garcia | Angie | M.D. | Parkland Health and Hospital Systems; University of Texas Southwestern Medical Center |
| Cha | Christine | PharmD. | Parkland Health and Hospital Systems |
| Mozaffari | Azadeh | PharmD. | Parkland Health and Hospital Systems |
| Hernandez | Rosa | PharmD., M.B.A. | Parkland Health and Hospital Systems |
| Cutrell | James | MD | University of Texas Southwestern Medical Center |
| Agbor | Barbine Tchamba Agbor | M.D. | University of Texas Southwestern Medical Center |
| Kim | Mina | PharmD. | University of Texas Southwestern Medical Center |
| DellaValle | Natalie | PharmD., B.C.P.S. | University of Texas Southwestern Medical Center |
| Gonzales | Sonia | PharmD., B.C.O.P. | University of Texas Southwestern Medical Center |
| Somboonwit | Charurut | M.D. | University of South Florida, Tampa General Hospital |
| Oxner | Asa | M.D. | University of South Florida, Tampa General Hospital |
| Guerra | Lucy | M.D. | University of South Florida, Tampa General Hospital |
| Hayes | Michael | PharmD. | University of South Florida, Tampa General Hospital |
| Nguyen | Thi | PharmD. | University of South Florida, Tampa General Hospital |
| Tran | Thanh | M.P.H. | University of South Florida, Tampa General Hospital |
| Pinto | Avenette |  | University of South Florida, Tampa General Hospital |
| Hatlen | Timothy | M.D. | Lundquist Institute for Biomedical Innovation |
| Anderson | Betty | B.S. | Lundquist Institute for Biomedical Innovation |
| Zepeda-Gutierrez | Ana | B.S. | Lundquist Institute for Biomedical Innovation |
| Martin | Dannae | B.A. | Lundquist Institute for Biomedical Innovation |
| Temblador | Cindi |  | Lundquist Institute for Biomedical Innovation |
| Cuenca | Avon | B.A. | Lundquist Institute for Biomedical Innovation |
| Tanoviceanu | Roxanne | PharmD. | Lundquist Institute for Biomedical Innovation |
| Prieto | Martha | PharmD. | Lundquist Institute for Biomedical Innovation |
| Guerrero | Mario | M.D. | Lundquist Institute for Biomedical Innovation |
| Martin | Dannae |  | Lundquist Institute for Biomedical Innovation |
| Daar | Eric | M.D. | Lundquist Institute for Biomedical Innovation |
| Correa | Ramiro |  | Lundquist Institute for Biomedical Innovation |
| Hartnell | Gabe |  | Lundquist Institute for Biomedical Innovation |
| Wortmann | Glenn | M.D. | Medstar Health Research Institute |
| Doshi | Saumil | M.D. | Medstar Health Research Institute |
| Moriarty | Theresa | M.S.N.R.N. | Medstar Health Research Institute |
| Gonzales | Melissa | C.R.C., III | Medstar Health Research Institute |
| Garman | Kristin | C.R.N. | Medstar Health Research Institute |
| Baker | Jason V. | M.D. | Hennepin Healthcare Research Institute |
| Frosch | Anne | M.D. | Hennepin Healthcare Research Institute |
| Goldsmith | Rachael | B.Sc. | Hennepin Healthcare Research Institute |
| Driver | Brian | M.D. | Hennepin Healthcare Research Institute |
| Frank | Christine | PharmD | Hennepin Healthcare Research Institute |
| Leviton | Tzivia | PharmD | Hennepin Healthcare Research Institute |
| Prekker | Matthew | M.D. | Hennepin Healthcare Research Institute |
| Jibrell | Hodan | B.Sc. | Hennepin Healthcare Research Institute |
| Lo | Melanie | M.D. | Hennepin Healthcare Research Institute |
| Klaphake | Jonathan | B.Sc. | Hennepin Healthcare Research Institute |
| Mackedanz | Shari | R.N. | Hennepin Healthcare Research Institute |
| Ngo | Linh | M.D. | Hennepin Healthcare Research Institute |
| Garcia-Myers | Kelly | B.Sc. | Hennepin Healthcare Research Institute |
| Kunisaki | Ken M. | M.D., M.S. | Minneapolis VA Medical Center |
| Wendt | Chris | M.D. | Minneapolis VA Medical Center |
| Melzer | Anne | M.D. | Minneapolis VA Medical Center |
| Wetherbee | Erin | M.D. | Minneapolis VA Medical Center |
| Drekonja | Dimitri | M.D. | Minneapolis VA Medical Center |
| Pragman | Alexa | M.D. | Minneapolis VA Medical Center |
| Hamel | Aimee | RN | Minneapolis VA Medical Center |
| Thielen | Abbie | PharmD. | Minneapolis VA Medical Center |
| Kunisaki | Ken M. | M.D., M.S. | Minneapolis VA Medical Center |
| Hassler | Miranda | B.A. | Minneapolis VA Medical Center |
| Walquist | Mary | B.S. | Minneapolis VA Medical Center |
| Augenbraun | Michael | M.D. | SUNY Downstate Medical Center |
| George | Jensen |  | SUNY Downstate Medical Center |
| Demeo | Lynette |  | SUNY Downstate Medical Center |
| Mishko | Motria | PharmD. | SUNY Downstate Medical Center |
| Thomas | Lorraine |  | SUNY Downstate Medical Center |
| Tatem | Luis |  | SUNY Downstate Medical Center |
| Dehovitz | Jack | M.D. | SUNY Downstate Medical Center |
| Abassi | Mahsa | D.O. | University of Minnesota |
| Leuck | Anne-Marie | M.D. | University of Minnesota |
| Rao | Via | M.S. | University of Minnesota |
| Pullen | Matthew | M.D. | University of Minnesota |
| Luke | Darlette | RPh | University of Minnesota |
| LaBar | Derek | PharmD, BCPS | University of Minnesota |
| Christiansen | Theresa | RPh | University of Minnesota |
| Howard | Diondra |  | University of Minnesota |
| Biswas | Kousick | Ph.D. | INSIGHT US Department of Veterans Affairs (VA) ICC |
| Harrington | Cristin | B.A. | INSIGHT US Department of Veterans Affairs (VA) ICC |
| Garcia | Amanda | M.P.H. | INSIGHT US Department of Veterans Affairs (VA) ICC |
| Bremer | Tammy |  | INSIGHT US Department of Veterans Affairs (VA) ICC |
| Burke | Tara |  | INSIGHT US Department of Veterans Affairs (VA) ICC |
| Koker | Brittany | B.S. | INSIGHT US Department of Veterans Affairs (VA) ICC |
| Davis-Karim | Anne | PharmD. | INSIGHT US Department of Veterans Affairs (VA) ICC |
| Pittman | David | B.E. | INSIGHT US Department of Veterans Affairs (VA) ICC |
| Vasudeva | Shikha S. | M.D. | INSIGHT US Department of Veterans Affairs (VA) ICC |
| Johnstone | Jaylynn R. | MPH | INSIGHT US Department of Veterans Affairs (VA) ICC |
| Agnetti | Kate | B.S. | INSIGHT US Department of Veterans Affairs (VA) ICC |
| Davis | Ruby | B.S. | INSIGHT US Department of Veterans Affairs (VA) ICC |
| Trautner | Barbara | M.D., Ph.D. | Michael E. DeBakey VA Medical Center, |
| Hines-Munson | Casey | B.S. | Michael E. DeBakey VA Medical Center, |
| Van | John | B.A. | Michael E. DeBakey VA Medical Center, |
| Dillon | Laura | M.Sc. | Michael E. DeBakey VA Medical Center, |
| Wang | Yiqun | B.S., M.A. | Michael E. DeBakey VA Medical Center, |
| Nagy-Agren | Stephanie | M.D. | Salem VA Medical Center |
| Vasudeva | Shikha | M.D. | Salem VA Medical Center |
| Ochalek | Tracy | B.S.N. | Salem VA Medical Center |
| Caldwell | Erin | D.O. | Salem VA Medical Center |
| Humerickhouse | Edward | M.D. | Salem VA Medical Center |
| Boone | David | D.O. | Salem VA Medical Center |
| McGraw | William | PharmD. | Salem VA Medical Center |
| Looney | David J. | M.D. | VA San Diego Healthcare System |
| Mehta | Sanjay R. | M.D. | VA San Diego Healthcare System |
| Johns | Scott Thompson | PharmD. | VA San Diego Healthcare System |
| St. John | Melissa |  | VA San Diego Healthcare System |
| Raceles | Jacqueline | C.C.R.C. | VA San Diego Healthcare System |
| Sear | Emily | B.S.N, R.N. | VA San Diego Healthcare System |
| Funk | Stephen | PharmD. | VA San Diego Healthcare System |
| Cesarini | Rosa |  | VA San Diego Healthcare System |
| Fang | Michelle | PharmD. | VA San Diego Healthcare System |
| Nicalo | Keith | R.N. | VA San Diego Healthcare System |
| Drake | Wonder | M.D. | VA TVHS Nashville Campus |
| Jones | Beatrice | M.S.N., R.N. | VA TVHS Nashville Campus |
| Holtman | Teresa | DPh. | VA TVHS Nashville Campus |
| Nguyen | Hien H. | M.D. | Sacramento VA Medical Center |
| Maniar | Archana | M.D. | Sacramento VA Medical Center |
| Johnson | Eric A. | M.D. | Sacramento VA Medical Center |
| Nguyen | Lam | B.A. | Sacramento VA Medical Center |
| Tran | Michelle T. | B.S. | Sacramento VA Medical Center |
| Barrett | Thomas W. | M.D., M.C.R. | Portland VA Health Care System |
| Johnston | Tera | B.S. | Portland VA Health Care System |
| Huggins | John T. | M.D. | Charleston VA Medical Center |
| Beiko | Tatsiana Y. | M.D. | Charleston VA Medical Center |
| Hughes | Heather Y. | M.D. | Charleston VA Medical Center |
| McManigle | William C. | M.D. | Charleston VA Medical Center |
| Tanner | Nichole T. | M.D. | Charleston VA Medical Center |
| Washburn | Ronald G. | M.D. | Charleston VA Medical Center |
| Ardelt | Magdalena | A.L.M. | Charleston VA Medical Center |
| Tuohy | Patricia A. | B.S. | Charleston VA Medical Center |
| Mixson | Jennifer L. | RPh. | Charleston VA Medical Center |
| Hinton | Charles G. | PharmD. | Charleston VA Medical Center |
| Thornley | Nicola | M.P.H. | Charleston VA Medical Center |
| Allen | Heather | R.N. | Charleston VA Medical Center |
| Elam | Shannon | R.N. | Charleston VA Medical Center |
| Boatman | Barry | R.N., O.C.N. | Charleston VA Medical Center |
| Baber | Brittany J. |  | Charleston VA Medical Center |
| Ryant | Rudell | M.B.A | Charleston VA Medical Center |
| Roller | Brentin | D.O. | Southern Arizona VA Health Care System |
| Nguyen | Chinh | M.D. | Southern Arizona VA Health Care System |
| Mikail | Amani Morgan | M.S.C.R.M. | Southern Arizona VA Health Care System |
| Hansen | Marivic | Research R.N. | Southern Arizona VA Health Care System |
| Lichtenberger | Paola | M.D. | Miami Bruce Carter VA Health CareSystem |
| Baracco | Gio | M.D. | Miami Bruce Carter VA Health CareSystem |
| Ramos | Carol | M.D. | Miami Bruce Carter VA Health CareSystem |
| Bjork | Lauren | PharmD. | Miami Bruce Carter VA Health CareSystem |
| Sueiro | Melyssa | M.Sc. | Miami Bruce Carter VA Health CareSystem |
| Tien | Phyllis | M.D. | San Francisco VA Health Care System |
| Freasier | Heather | M.Sc., R.D. | San Francisco VA Health Care System |
| Buck | Theresa | M.D. | Bay Pines VA Healthcare System |
| Nekach | Hafida | M.D. | Bay Pines VA Healthcare System |
| Holodniy | Mark | M.D., F.A.C.P., F.I.D.S.A. | Veterans Affairs Palo Alto Health Care System |
| Chary | Aarthi | M.D. | Veterans Affairs Palo Alto Health Care System |
| Lu | Kan | PharmD. | Veterans Affairs Palo Alto Health Care System |
| Peters | Theresa | R.N., M.S., C.C.R.C. | Veterans Affairs Palo Alto Health Care System |
| Lopez | Jessica | C.C.R.C. | Veterans Affairs Palo Alto Health Care System |
| Tan | Susanna Yu | M.D. | VA Long Beach Healthcare System |
| Lee | Robert H. | M.D. | VA Long Beach Healthcare System |
| Asghar | Aliya | M.P.H | VA Long Beach Healthcare System |
| Isip | Tasadduq Karim Karyn | B.A. | VA Long Beach Healthcare System |
| Le | Katherine | PharmD. | VA Long Beach Healthcare System |
| Nguyen | Thao | PharmD. | VA Long Beach Healthcare System |
| Wong | Shinn | PharmD. | VA Long Beach Healthcare System |
| Raben | Dorthe | M.Sc. | INSIGHT Copenhagen ICC, CHIP (Centre of Excellence for Health, Immunity and Infections), Rigshospitalet, University of Copenhagen, Copenhagen, Denmark |
| Murray | Daniel D. | Ph.D. | INSIGHT Copenhagen ICC, CHIP (Centre of Excellence for Health, Immunity and Infections), Rigshospitalet, University of Copenhagen, Copenhagen, Denmark |
| Jensen | Tomas O. | M.D. | INSIGHT Copenhagen ICC, CHIP (Centre of Excellence for Health, Immunity and Infections), Rigshospitalet, University of Copenhagen, Copenhagen, Denmark |
| Peters | Lars | M.D., Ph.D., D.M.Sc. | INSIGHT Copenhagen ICC, CHIP (Centre of Excellence for Health, Immunity and Infections), Rigshospitalet, University of Copenhagen, Copenhagen, Denmark |
| Aagaard | Bitten | B.Sc.N. | INSIGHT Copenhagen ICC, CHIP (Centre of Excellence for Health, Immunity and Infections), Rigshospitalet, University of Copenhagen, Copenhagen, Denmark |
| Nielsen | Charlotte B. |  | INSIGHT Copenhagen ICC, CHIP (Centre of Excellence for Health, Immunity and Infections), Rigshospitalet, University of Copenhagen, Copenhagen, Denmark |
| Krapp | Katharina | Ph.D. | INSIGHT Copenhagen ICC, CHIP (Centre of Excellence for Health, Immunity and Infections), Rigshospitalet, University of Copenhagen, Copenhagen, Denmark |
| Nykjær | Bente Rosdahl |  | INSIGHT Copenhagen ICC, CHIP (Centre of Excellence for Health, Immunity and Infections), Rigshospitalet, University of Copenhagen, Copenhagen, Denmark |
| Olsson | Christina |  | INSIGHT Copenhagen ICC, CHIP (Centre of Excellence for Health, Immunity and Infections), Rigshospitalet, University of Copenhagen, Copenhagen, Denmark |
| Kanne | Katja Lisa | M.Sc., B.Sc.N. | INSIGHT Copenhagen ICC, CHIP (Centre of Excellence for Health, Immunity and Infections), Rigshospitalet, University of Copenhagen, Copenhagen, Denmark |
| Grevsen | Anne Louise | M.Sc., Dent. | INSIGHT Copenhagen ICC, CHIP (Centre of Excellence for Health, Immunity and Infections), Rigshospitalet, University of Copenhagen, Copenhagen, Denmark |
| Joensen | Zillah Maria | B.Sc.N. | INSIGHT Copenhagen ICC, CHIP (Centre of Excellence for Health, Immunity and Infections), Rigshospitalet, University of Copenhagen, Copenhagen, Denmark |
| Bruun | Tina | B.Sc.N. | INSIGHT Copenhagen ICC, CHIP (Centre of Excellence for Health, Immunity and Infections), Rigshospitalet, University of Copenhagen, Copenhagen, Denmark |
| Bojesen | Ane |  | INSIGHT Copenhagen ICC, CHIP (Centre of Excellence for Health, Immunity and Infections), Rigshospitalet, University of Copenhagen, Copenhagen, Denmark |
| Woldbye | Frederik |  | INSIGHT Copenhagen ICC, CHIP (Centre of Excellence for Health, Immunity and Infections), Rigshospitalet, University of Copenhagen, Copenhagen, Denmark |
| Normand, | Nick E. | B.Sc. | INSIGHT Copenhagen ICC, CHIP (Centre of Excellence for Health, Immunity and Infections), Rigshospitalet, University of Copenhagen, Copenhagen, Denmark |
| Esman | Frederik V.L. | B.Sc. | INSIGHT Copenhagen ICC, CHIP (Centre of Excellence for Health, Immunity and Infections), Rigshospitalet, University of Copenhagen, Copenhagen, Denmark |
| Benfield | Thomas | M.D, D.M.Sc. | Denmark Copenhagen University Hospital - Amager and Hvidovre, Center of Research & Disruption of Infectious Diseases, Department of Infectious Diseases |
| Clausen | Clara Lundetoft | M.D. | Denmark Copenhagen University Hospital - Amager and Hvidovre, Center of Research & Disruption of Infectious Diseases, Department of Infectious Diseases |
| Hovmand | Nichlas | M.D. | Denmark Copenhagen University Hospital - Amager and Hvidovre, Center of Research & Disruption of Infectious Diseases, Department of Infectious Diseases |
| Israelsen | Simone Bastrup | M.D. | Denmark Copenhagen University Hospital - Amager and Hvidovre, Center of Research & Disruption of Infectious Diseases, Department of Infectious Diseases |
| Iversen | Katrine | M.D. | Denmark Copenhagen University Hospital - Amager and Hvidovre, Center of Research & Disruption of Infectious Diseases, Department of Infectious Diseases |
| Leding | Caecilie | M.D. | Denmark Copenhagen University Hospital - Amager and Hvidovre, Center of Research & Disruption of Infectious Diseases, Department of Infectious Diseases |
| Pedersen | Karen Brorup | M.D. | Denmark Copenhagen University Hospital - Amager and Hvidovre, Center of Research & Disruption of Infectious Diseases, Department of Infectious Diseases |
| Thorlacius-Ussing | Louise | M.D. | Denmark Copenhagen University Hospital - Amager and Hvidovre, Center of Research & Disruption of Infectious Diseases, Department of Infectious Diseases |
| Tinggaard | Michaela | M.D. | Denmark Copenhagen University Hospital - Amager and Hvidovre, Center of Research & Disruption of Infectious Diseases, Department of Infectious Diseases |
| Tingsgard | Sandra | M.D. | Denmark Copenhagen University Hospital - Amager and Hvidovre, Center of Research & Disruption of Infectious Diseases, Department of Infectious Diseases |
| Krohn-Dehli | Louise | R.N. | Denmark Copenhagen University Hospital - Amager and Hvidovre, Center of Research & Disruption of Infectious Diseases, Department of Infectious Diseases |
| Pedersen | Dorthe | R.N. | Denmark Copenhagen University Hospital - Amager and Hvidovre, Center of Research & Disruption of Infectious Diseases, Department of Infectious Diseases |
| Villadsen | Signe | R.N. | Denmark Copenhagen University Hospital - Amager and Hvidovre, Center of Research & Disruption of Infectious Diseases, Department of Infectious Diseases |
| Jensen | Jens-Ulrik Staehr | M.D., Ph.D. | Herlev-Gentofte Hospital, Respiratory Medicine Section, Department of Internal Medicine |
| Overgaard | Rikke | R.N. | Herlev-Gentofte Hospital, Respiratory Medicine Section, Department of Internal Medicine |
| Rastoder | Ema | M.D. | Herlev-Gentofte Hospital, Respiratory Medicine Section, Department of Internal Medicine |
| Heerfordt | Christian | M.D. | Herlev-Gentofte Hospital, Respiratory Medicine Section, Department of Internal Medicine |
| Hedsund | Caroline | M.D | Herlev-Gentofte Hospital, Respiratory Medicine Section, Department of Internal Medicine |
| Ronn | Christian Phillip | M.D. | Herlev-Gentofte Hospital, Respiratory Medicine Section, Department of Internal Medicine |
| Kamstrup | Peter Thobias | M.D. | Herlev-Gentofte Hospital, Respiratory Medicine Section, Department of Internal Medicine |
| Hogsberg | Dorthe Sandbaek | R.N. | Herlev-Gentofte Hospital, Respiratory Medicine Section, Department of Internal Medicine |
| Bergsoe | Christina | B.Sc. | Herlev-Gentofte Hospital, Respiratory Medicine Section, Department of Internal Medicine |
| Søborg | Christian | M.D., Ph.D. | Herlev-Gentofte Hospital, Respiratory Medicine Section, Department of Internal Medicine |
| Hissabu | Nuria M.S. | B.Sc. | Herlev-Gentofte Hospital, Respiratory Medicine Section, Department of Internal Medicine |
| Arp | Bodil C. | B.Sc. | Herlev-Gentofte Hospital, Respiratory Medicine Section, Department of Internal Medicine |
| Ostergaard | Lars | M.D., Ph.D., D.M.Sc. | Aarhus Universitetshospital, Skejby |
| Staerke | Nina Breinholt | M.D. | Aarhus Universitetshospital, Skejby |
| Yehdego | Yordanos | R.N. | Aarhus Universitetshospital, Skejby |
| Sondergaard | Ane | R.N. | Aarhus Universitetshospital, Skejby |
| Johansen | Isik S. | M.D., D.M.Sc. | Odense University Hospital, Department of Infectious Diseases |
| Arnholdt Pedersen | Andreas | M.D. | Odense University Hospital, Department of Infectious Diseases |
| Knudtzen | Fredrikke C. | M.D. | Odense University Hospital, Department of Infectious Diseases |
| Larsen | Lykke | M.D. | Odense University Hospital, Department of Infectious Diseases |
| Hertz | Mathias A. | M.D. | Odense University Hospital, Department of Infectious Diseases |
| Fabricius | Thilde | M.D. | Odense University Hospital, Department of Infectious Diseases |
| Holden | Inge K. | M.D., Ph.D. | Odense University Hospital, Department of Infectious Diseases |
| Lindvig | Susan O. | M.Sc. | Odense University Hospital, Department of Infectious Diseases |
| Helleberg | Marie | M.D., Ph.D., D.M.Sc. | Dept. of Infectious Diseases, Rigshospitalet, Copenhagen University Hospital |
| Gerstoft | Jan | M.D., D.M.Sc. | Dept. of Infectious Diseases, Rigshospitalet, Copenhagen University Hospital |
| Kirk | Ole | M.D., D.M.Sc. | Dept. of Infectious Diseases, Rigshospitalet, Copenhagen University Hospital |
| Bruun | Tina | R.N., M.Sc., PH. | Dept. of Infectious Diseases, Rigshospitalet, Copenhagen University Hospital |
| Jensen | Tomas Ostergaard | M.D. | North Zealand University Hospital, Department of Pulmonary and Infectious Diseases |
| Madsen | Birgitte Lindegaard | M.D. | North Zealand University Hospital, Department of Pulmonary and Infectious Diseases |
| Pedersen | Thomas Ingemann | M.D. | North Zealand University Hospital, Department of Pulmonary and Infectious Diseases |
| Harboe | Zitta Barrella | M.D. | North Zealand University Hospital, Department of Pulmonary and Infectious Diseases |
| Roge | Birgit Thorup | M.D., Ph.D. | Kolding Hospital, Department of Medicine |
| Hansen | Thomas Michael | M.D. | Kolding Hospital, Department of Medicine |
| Glesner | Matilde Kanstrup | M.D. | Kolding Hospital, Department of Medicine |
| Lofberg | Sandra Valborg | M.D. | Kolding Hospital, Department of Medicine |
| Nielsen | Ariella Denize | M.D. | Kolding Hospital, Department of Medicine |
| Leicht von Huth | Sebastian | M.D., Ph.D. | Kolding Hospital, Department of Medicine |
| Nielsen | Henrik | M.D., D.M.Sci. | Aalborg University Hospital, Department of Infectious Diseases |
| Thisted | Rikke Krog | R.N. | Aalborg University Hospital, Department of Infectious Diseases |
| Petersen | Kristine Toft | R.N. | Aalborg University Hospital, Department of Infectious Diseases |
| Juhl | Maria Ruwald | R.N | Aalborg University Hospital, Department of Infectious Diseases |
| Podlekareva | Daria | M.D., Ph.D. | Department of Respiratory Medicine, Bispebjerg Hospital, Copenhagen, Denmark |
| Johnsen | Stine | M.D., Ph.D. | Department of Respiratory Medicine, Bispebjerg Hospital, Copenhagen, Denmark |
| Andreassen | Helle Frost | M.D., Ph.D. | Department of Respiratory Medicine, Bispebjerg Hospital, Copenhagen, Denmark |
| Pedersen | Lars | M.D., Ph.D. | Department of Respiratory Medicine, Bispebjerg Hospital, Copenhagen, Denmark |
| Lindnér | Cecilia Ebba Clara Ellinor | M.D. | Department of Respiratory Medicine, Bispebjerg Hospital, Copenhagen, Denmark |
| Wiese | Lothar | M.D., Ph.D. | Department of Infectious Diseases, Zealand University Hospital Roskilde and Department of Internal Medicine, Zealand University Hospital Koge |
| Knudsen | Lene Surland | M.D., Ph.D. | Department of Infectious Diseases, Zealand University Hospital Roskilde and Department of Internal Medicine, Zealand University Hospital Koge |
| Nytofte | Nikolaj Julian Skrøder | M.D., Ph.D. | Department of Infectious Diseases, Zealand University Hospital Roskilde and Department of Internal Medicine, Zealand University Hospital Koge |
| Havmøller | Signe Ravn | M.D. | Department of Infectious Diseases, Zealand University Hospital Roskilde and Department of Internal Medicine, Zealand University Hospital Koge |
| Expósito | Maria | B.Sc. | Spain INSIGHT SCC Spain, Hospital Universitari Germans Trias i Pujol, Badalona |
| Badillo | José | B.Sc. | Spain INSIGHT SCC Spain, Hospital Universitari Germans Trias i Pujol, Badalona |
| Martínez | Ana | B.Sc. | Spain INSIGHT SCC Spain, Hospital Universitari Germans Trias i Pujol, Badalona |
| Abad | Elena | B.Sc. | Spain INSIGHT SCC Spain, Hospital Universitari Germans Trias i Pujol, Badalona |
| Chamorro | Ana | B.Sc. | Spain INSIGHT SCC Spain, Hospital Universitari Germans Trias i Pujol, Badalona |
| Figuerola | Ariadna | B.Sc. | Spain INSIGHT SCC Spain, Hospital Universitari Germans Trias i Pujol, Badalona |
| Mateu | Lourdes | M.D., Ph.D. | Hospital Universitari Germans Trias i Pujol, Badalona |
| España | Sergio | M.D. | Hospital Universitari Germans Trias i Pujol, Badalona |
| Lucero | Maria Constanza | MD., Ph.D. | Hospital Universitari Germans Trias i Pujol, Badalona |
| Santos | José Ramón | M.D., Ph.D. | Hospital Universitari Germans Trias i Pujol, Badalona |
| Lladós | Gemma | M.D. | Hospital Universitari Germans Trias i Pujol, Badalona |
| Lopez | Cristina | M.D., Ph.D. | Hospital Universitari Germans Trias i Pujol, Badalona |
| Carabias | Lydia | M.D. | Hospital Universitari Germans Trias i Pujol, Badalona |
| Molina-Morant | Daniel | M.D., Ph.D. | Hospital Universitari Germans Trias i Pujol, Badalona |
| Loste | Cora | M.D., Ph.D. | Hospital Universitari Germans Trias i Pujol, Badalona |
| Bracke | Carmen | M.D. | Hospital Universitari Germans Trias i Pujol, Badalona |
| Siles | Adrian | B.Sc. | Hospital Universitari Germans Trias i Pujol, Badalona |
| Fernández-Cruz | Eduardo | M.D., Ph.D. | Hospital General Universitario Gregorio Marañón, Madrid |
| Natale | Marisa Di | M.D. | Hospital General Universitario Gregorio Marañón, Madrid |
| Padure | Sergiu |  | Hospital General Universitario Gregorio Marañón, Madrid |
| Gomez | Jimena | M.D. | Hospital General Universitario Gregorio Marañón, Madrid |
| Ausin | Cristina | M.D. | Hospital General Universitario Gregorio Marañón, Madrid |
| Cervilla | Eva | M.D. | Hospital General Universitario Gregorio Marañón, Madrid |
| Balastegui | Héctor | M.D. | Hospital General Universitario Gregorio Marañón, Madrid |
| Sainz | Carmen Rodríguez | Ph.D. | Hospital General Universitario Gregorio Marañón, Madrid |
| Lopez | Paco | M.D. | Hospital General Universitario Gregorio Marañón, Madrid |
| Carbone | Javier | M.D., Ph.D. | Hospital General Universitario Gregorio Marañón, Madrid |
| Escobar | Mariam | R.N. | Hospital General Universitario Gregorio Marañón, Madrid |
| Balerdi | Leire | M.D. | Barcelona Institute for Global Health (ISGlobal), Hospital Clínic - Universitat de Barcelona, Barcelona |
| Legarda | Almudena |  | Barcelona Institute for Global Health (ISGlobal), Hospital Clínic - Universitat de Barcelona, Barcelona |
| Roldan | Montserrat |  | Barcelona Institute for Global Health (ISGlobal), Hospital Clínic - Universitat de Barcelona, Barcelona |
| Letona | Laura | M.D. | Barcelona Institute for Global Health (ISGlobal), Hospital Clínic - Universitat de Barcelona, Barcelona |
| Muñoz | José | M.D., Ph.D. | Barcelona Institute for Global Health (ISGlobal), Hospital Clínic - Universitat de Barcelona, Barcelona |
| Camprubí | Daniel | M.D. | Barcelona Institute for Global Health (ISGlobal), Hospital Clínic - Universitat de Barcelona, Barcelona |
| Arribas | Jose R. | M.D. | Hospital Universitario La Paz, IdiPAZ, Madrid |
| Sánchez | Rocio Montejano | M.D., Ph.D. | Hospital Universitario La Paz, IdiPAZ, Madrid |
| Díaz-Pollán | Beatriz | M.D., Ph.D. | Hospital Universitario La Paz, IdiPAZ, Madrid |
| Stewart | Stefan Mark | M.D. | Hospital Universitario La Paz, IdiPAZ, Madrid |
| Garcia | Irene | M.D. | Hospital Universitario La Paz, IdiPAZ, Madrid |
| Borobia | Alberto | M.D., Ph.D. | Hospital Universitario La Paz, IdiPAZ, Madrid |
| Mora-Rillo | Marta | M.D., Ph.D. | Hospital Universitario La Paz, IdiPAZ, Madrid |
| Estrada | Vicente | M.D., Ph.D. | Hospital Clínico San Carlos, Madrid |
| Cabello | Noemi | M.D. | Hospital Clínico San Carlos, Madrid |
| Nuñez-Orantos | M.J. | M.D. | Hospital Clínico San Carlos, Madrid |
| Sagastagoitia | I. | M.D. | Hospital Clínico San Carlos, Madrid |
| Homen | J.R. | MD | Hospital Clínico San Carlos, Madrid |
| Orviz | E. | MD. | Hospital Clínico San Carlos, Madrid |
| Montalvá | Adrián Sánchez | M.D., P.hD. | Hospital Universitary Vall d'Hebron, Barcelona |
| Espinosa-Pereiro | Juan | M.D. | Hospital Universitary Vall d'Hebron, Barcelona |
| Bosch-Nicolau | Pau | M.D. | Hospital Universitary Vall d'Hebron, Barcelona |
| Salvador | Fernando | M.D., P.hD. | Hospital Universitary Vall d'Hebron, Barcelona |
| Burgos | Joaquin | M.D., Ph.D. | Hospital Universitary Vall d'Hebron, Barcelona |
| Morales-Rull | Jose Luis | M.D., Ph.D. | Internal Medicine Department. University Hospital Arnau de Vilanova, Lleida |
| Pena | Anna Maria Moreno | M.D. | Internal Medicine Department. University Hospital Arnau de Vilanova, Lleida |
| Acosta | Cristina | M.D. | Internal Medicine Department. University Hospital Arnau de Vilanova, Lleida |
| Solé-Felip | Cristina | M.D. | Internal Medicine Department. University Hospital Arnau de Vilanova, Lleida |
| Horcajada | Juan P. | M.D., Ph.D. | Hospital del Mar-IMIM, Barcelona |
| Sendra | Elena | M.D. | Hospital del Mar-IMIM, Barcelona |
| Castañeda | Silvia | M.D. | Hospital del Mar-IMIM, Barcelona |
| López-Montesinos | Inmaculada | M.D. | Hospital del Mar-IMIM, Barcelona |
| Gómez-Junyent | Joan | M.D., Ph.D. | Hospital del Mar-IMIM, Barcelona |
| Gonzáles | Carlota Gudiol | M.D. | Infectious Diseases Department, Bellvitge University Hospital, IDIBELL, University of Barcelona, CIBERINFEC |
| Cuervo | Guilermo | M.D., Ph.D. | Infectious Diseases Department, Bellvitge University Hospital, IDIBELL, University of Barcelona, CIBERINFEC |
| Pujol | Miquel | M.D., Ph.D. | Infectious Diseases Department, Bellvitge University Hospital, IDIBELL, University of Barcelona, CIBERINFEC |
| Carratalà | Jordi | M.D., Ph.D. | Infectious Diseases Department, Bellvitge University Hospital, IDIBELL, University of Barcelona, CIBERINFEC |
| Videla | Sebastià | M.D., Ph.D. | Infectious Diseases Department, Bellvitge University Hospital, IDIBELL, University of Barcelona, CIBERINFEC |
| Günthard | Huldrych | M.D. | Switzerland Department of Infectious Diseases and Hospital Epidemiology, University Hospital Zurich and Institute of Medical Virology, University of Zurich, Zurich Switzerland |
| Braun | Dominique L. | M.D. | Switzerland Department of Infectious Diseases and Hospital Epidemiology, University Hospital Zurich and Institute of Medical Virology, University of Zurich, Zurich Switzerland |
| West | Emily | M.D. | Switzerland Department of Infectious Diseases and Hospital Epidemiology, University Hospital Zurich and Institute of Medical Virology, University of Zurich, Zurich Switzerland |
| M’Rabeth-Bensalah | Khadija | M.D. | Switzerland Department of Infectious Diseases and Hospital Epidemiology, University Hospital Zurich and Institute of Medical Virology, University of Zurich, Zurich Switzerland |
| Eichinger | Mareile L. | M.D. | Switzerland Department of Infectious Diseases and Hospital Epidemiology, University Hospital Zurich and Institute of Medical Virology, University of Zurich, Zurich Switzerland |
| Grüttner-Durmaz | Manuela | R.N. | Switzerland Department of Infectious Diseases and Hospital Epidemiology, University Hospital Zurich and Institute of Medical Virology, University of Zurich, Zurich Switzerland |
| Grube | Christina | R.N. | Switzerland Department of Infectious Diseases and Hospital Epidemiology, University Hospital Zurich and Institute of Medical Virology, University of Zurich, Zurich Switzerland |
| Zink | Veronika | M.Sc., pharmacist | Switzerland Department of Infectious Diseases and Hospital Epidemiology, University Hospital Zurich and Institute of Medical Virology, University of Zurich, Zurich Switzerland |
| Goes | Josefine | pharmacist | Switzerland Department of Infectious Diseases and Hospital Epidemiology, University Hospital Zurich and Institute of Medical Virology, University of Zurich, Zurich Switzerland |
| Fätkenheuer | Gerd | M.D. | Department I of Internal Medicine, Division of Infectious Diseases, University of Cologne, Germany |
| Malin | Jakob J. | M.D. | Department I of Internal Medicine, Division of Infectious Diseases, University of Cologne, Germany |
| Tsertsvadze | Tengiz | M.D., Ph.D. | Georgia SCC, Infectious Diseases, AIDS and Clinical Immunology Research Center, Tbilisi, Georgia |
| Abutidze | Akaki | M.D., M.P.H., Ph.D. | Georgia SCC, Infectious Diseases, AIDS and Clinical Immunology Research Center, Tbilisi, Georgia |
| Chkhartishvili | Nikoloz | M.D., M.S., Ph.D. | Georgia SCC, Infectious Diseases, AIDS and Clinical Immunology Research Center, Tbilisi, Georgia |
| Metchurtchlishvili | Revaz | M.D. | Georgia SCC, Infectious Diseases, AIDS and Clinical Immunology Research Center, Tbilisi, Georgia |
| Endeladze | Marina | M.D. | Georgia SCC, Infectious Diseases, AIDS and Clinical Immunology Research Center, Tbilisi, Georgia |
| Paciorek | Marcin | M.D., Ph.D. | Poland SCC, Wojewodzki Szpital Zakazny Warsaw |
| Bursa | Dominik | M.D., Ph.D. | Poland SCC, Wojewodzki Szpital Zakazny Warsaw |
| Krogulec | Dominika | M.D. | Poland SCC, Wojewodzki Szpital Zakazny Warsaw |
| Pulik | Piotr | M.D. | Poland SCC, Wojewodzki Szpital Zakazny Warsaw |
| Ignatowska | Anna | M.D. | Poland SCC, Wojewodzki Szpital Zakazny Warsaw |
| Horban | Andrzej | M.D., Ph.D. | Poland SCC, Wojewodzki Szpital Zakazny Warsaw |
| Bakowska | Elzbieta | M.D. | Poland SCC, Wojewodzki Szpital Zakazny Warsaw |
| Kowaska | Justyna | M.D., Ph.D. | Poland SCC, Wojewodzki Szpital Zakazny Warsaw |
| Bednarska | Agnieszka | M.D., Ph.D. | Poland SCC, Wojewodzki Szpital Zakazny Warsaw |
| Jurek | Natalia | M.D. | Poland SCC, Wojewodzki Szpital Zakazny Warsaw |
| Skrzat-Klapaczynska | Agata | M.D., Ph.D. | Poland SCC, Wojewodzki Szpital Zakazny Warsaw |
| Bienkowski | Carlo | M.D. | Poland SCC, Wojewodzki Szpital Zakazny Warsaw |
| Hackiewicz | Malgorzata | M.D. | Poland SCC, Wojewodzki Szpital Zakazny Warsaw |
| Makowiecki | Michal | M.D. | Poland SCC, Wojewodzki Szpital Zakazny Warsaw |
| Platowski | Antoni | M.D. | Poland SCC, Wojewodzki Szpital Zakazny Warsaw |
| Fishchuk | Roman | M.D | Ukraine Central City Clinical Hospital of Ivano-Frankivsk City, Ukraine |
| Kobrynska | Olena | M.D | Ukraine Central City Clinical Hospital of Ivano-Frankivsk City, Ukraine |
| Levandovska | Khrystyna | M.D | Ukraine Central City Clinical Hospital of Ivano-Frankivsk City, Ukraine |
| Kirieieva | Ivanna |  | Ukraine Central City Clinical Hospital of Ivano-Frankivsk City, Ukraine |
| Kuziuk | Mykhailo |  | Ukraine Central City Clinical Hospital of Ivano-Frankivsk City, Ukraine |
| Naucler | Pontus | M.D., Ph.D. | Sweden, Dept. Of Infectious Diseases, Karolinska University Hospital and Division of Infectious Diseases, Dept. Of Medicine, Solna, Karolinska Instituttet |
| Perlhamre | Emma | M.Sc. | Sweden, Dept. Of Infectious Diseases, Karolinska University Hospital and Division of Infectious Diseases, Dept. Of Medicine, Solna, Karolinska Instituttet |
| Mazouch | Lotta | M.Sc. | Sweden, Dept. Of Infectious Diseases, Karolinska University Hospital and Division of Infectious Diseases, Dept. Of Medicine, Solna, Karolinska Instituttet |
| Matthews | Gail | MRCP, PhD | INSIGHT Sydney ICC, The Kirby Institute, University of New South Wales, Sydney, Australia |
| Kelleher | Anthony | M.B.B.S., Ph.D. | INSIGHT Sydney ICC, The Kirby Institute, University of New South Wales, Sydney, Australia |
| Polizzotto | Mark | M.D., Ph.D. | INSIGHT Sydney ICC, The Kirby Institute, University of New South Wales, Sydney, Australia |
| Carey | Catherine | B.A., M.Sc. | INSIGHT Sydney ICC, The Kirby Institute, University of New South Wales, Sydney, Australia |
| Chang | Christina C. | M.D., Ph.D. | INSIGHT Sydney ICC, The Kirby Institute, University of New South Wales, Sydney, Australia |
| Hough | Sally | B.Sc. | INSIGHT Sydney ICC, The Kirby Institute, University of New South Wales, Sydney, Australia |
| Virachit | Sophie | B.Sc., Ph.D. | INSIGHT Sydney ICC, The Kirby Institute, University of New South Wales, Sydney, Australia |
| Davidson | Sarah | B.N. | INSIGHT Sydney ICC, The Kirby Institute, University of New South Wales, Sydney, Australia |
| Bice | Daniel J. | B.MSc. | INSIGHT Sydney ICC, The Kirby Institute, University of New South Wales, Sydney, Australia |
| Ognenovska | Katherine | B.Sc., Ph.D. | INSIGHT Sydney ICC, The Kirby Institute, University of New South Wales, Sydney, Australia |
| Cabrera | Gesalit | B.MSc., M.I.P.H. | INSIGHT Sydney ICC, The Kirby Institute, University of New South Wales, Sydney, Australia |
| Flynn | Ruth | B.App.Sc., M.App.Sc. | INSIGHT Sydney ICC, The Kirby Institute, University of New South Wales, Sydney, Australia |
| Young | Barnaby E. | M.B.B.S., Ph.D. | National Centre for Infectious Diseases; Tan Tock Seng Hospital; Lee Kong Chian School of Medicine; Singapore |
| Chia | Po Ying | M.B.B.S. | National Centre for Infectious Diseases; Tan Tock Seng Hospital; Lee Kong Chian School of Medicine; Singapore |
| Lee | Tau Hong | M.B.B.S. | National Centre for Infectious Diseases; Tan Tock Seng Hospital; Lee Kong Chian School of Medicine; Singapore |
| Lin | Ray J. | M.B.B.S. | National Centre for Infectious Diseases; Tan Tock Seng Hospital; Woodlands Health; Singapore |
| Lye | David C. | M.B.B.S. | National Centre for Infectious Diseases; Tan Tock Seng Hospital; Yong Loo Lin School of Medicine; Lee Kong Chian School of Medicine; Singapore |
| Ong | Sean W.X. | M.B.B.S. | National Centre for Infectious Diseases; Tan Tock Seng Hospital; Singapore |
| Puah | Ser Hon | M.B.B.S. | Tan Tock Seng Hospital; Singapore |
| Yeo | Tsin Wen | M.B.B.S., Ph.D. | National Centre for Infectious Diseases; Tan Tock Seng Hospital; Lee Kong Chian School of Medicine; Singapore |
| Diong | Shiau Hui | B.Bio., M.Sc. | National Centre for Infectious Diseases; Tan Tock Seng Hospital; Singapore |
| Ongko | Juwinda | B.Sc. | National Centre for Infectious Diseases; Tan Tock Seng Hospital; Singapore |
| Yeo | He Ping | B.Sc. | National Centre for Infectious Diseases; Tan Tock Seng Hospital; Singapore |
| Eriobu | Nnakelu | M.D., M.P.H. | Institute of Human Virology-Nigeria (IHVN) |
| Kwaghe | Vivian | M.D. | Institute of Human Virology-Nigeria (IHVN) |
| Zaiyad | Habib | M.D. | Institute of Human Virology-Nigeria (IHVN) |
| Idoko | Godwin | M.D. | Institute of Human Virology-Nigeria (IHVN) |
| Uche | Blessing | R.M., R.N. | Institute of Human Virology-Nigeria (IHVN) |
| Selvamuthu | Poongulali | M.B.B.S., M.Sc., Ph.D. | Chennai Antiviral Research and Treatment Clinical Research Site, India |
| Kumarasamy | Nagalingeswaran | M.B.B.S., Ph.D. | Chennai Antiviral Research and Treatment Clinical Research Site, India |
| Beulah | Faith Ester | B.Sc., M.A., M.Sc. | Chennai Antiviral Research and Treatment Clinical Research Site, India |
| Govindarajan | Narayan | B.Pharm. | Chennai Antiviral Research and Treatment Clinical Research Site, India |
| Mariyappan | Kowsalya | B.Pharm. | Chennai Antiviral Research and Treatment Clinical Research Site, India |
| Losso | Marcelo H. | M.D., M.S. | INSIGHT SCC Argentina, Coordinación en Investigación Clínica Académica en Latinoamérica |
| Abela | Cecilia | R.N., B.S.N. | INSIGHT SCC Argentina, Coordinación en Investigación Clínica Académica en Latinoamérica |
| Moretto | Renzo | M.D. | INSIGHT SCC Argentina, Coordinación en Investigación Clínica Académica en Latinoamérica |
| Belloc | Carlos G. | B.Sc., Ph.D. | INSIGHT SCC Argentina, Coordinación en Investigación Clínica Académica en Latinoamérica |
| Ludueña | Jael | T.B.A. | INSIGHT SCC Argentina, Coordinación en Investigación Clínica Académica en Latinoamérica |
| Amar | Josefina |  | INSIGHT SCC Argentina, Coordinación en Investigación Clínica Académica en Latinoamérica |
| Losso | Marcelo H. | M.D., M.S. | Hospital General de Agudos JM Ramos Mejia, Buenos Aires |
| Toibaro | Javier | M.D., B.C. | Hospital General de Agudos JM Ramos Mejia, Buenos Aires |
| Macias | Laura Moreno | M.D. | Hospital General de Agudos JM Ramos Mejia, Buenos Aires |
| Fernandez | Lucia | M.D. | Hospital General de Agudos JM Ramos Mejia, Buenos Aires |
| Frare | Pablo S. | M.D. | Hospital General de Agudos JM Ramos Mejia, Buenos Aires |
| Chaio | Sebastian R. | M.D. | Hospital General de Agudos JM Ramos Mejia, Buenos Aires |
| Pachioli | Valeria | M.D. | Hospital General de Agudos JM Ramos Mejia, Buenos Aires |
| Timpano | Stella M. | B.Pharm. | Hospital General de Agudos JM Ramos Mejia, Buenos Aires |
| Sanchez | Marisa del Lujan | M.D. | Hospital Italiano de Buenos Aires, Buenos Aires |
| Sierra | Mariana de Paz | M.D. | Hospital Italiano de Buenos Aires, Buenos Aires |
| Stanek | Vanina | M.D. | Hospital Italiano de Buenos Aires, Buenos Aires |
| Belloso | Waldo | M.D. | Hospital Italiano de Buenos Aires, Buenos Aires |
| Cilenti | Flavia L. |  | Hospital Italiano de Buenos Aires, Buenos Aires |
| Valentini | Ricardo N. | M.D. | Centro de Educacion Medica e Investigaciones Clinicas, Buenos Aires |
| Stryjewski | Martin E. | M.D. | Centro de Educacion Medica e Investigaciones Clinicas, Buenos Aires |
| Locatelli | Nicolas | M.D. | Centro de Educacion Medica e Investigaciones Clinicas, Buenos Aires |
| Soler Riera | Maria C. | M.D. | Centro de Educacion Medica e Investigaciones Clinicas, Buenos Aires |
| Salgado | Clara | M.D. | Centro de Educacion Medica e Investigaciones Clinicas, Buenos Aires |
| Baeck | Ines M. | M.D. | Centro de Educacion Medica e Investigaciones Clinicas, Buenos Aires |
| Di Castelnuovo | Valentina | M.D. | Centro de Educacion Medica e Investigaciones Clinicas, Buenos Aires |
| Zarza | Stella M. | M.D. | Centro de Educacion Medica e Investigaciones Clinicas, Buenos Aires |
| Hudson | Fleur | B.A. | INSIGHT London ICC, MRC Clinical Trials Unit at UC, London, UK |
| Parmar | Mahesh K.B. | Ph.D. | INSIGHT London ICC, MRC Clinical Trials Unit at UC, London, UK |
| Goodman | Anna L. | F.R.C.P., Dphil | INSIGHT London ICC, MRC Clinical Trials Unit at UC, London, UK |
| Badrock | Jonathan | B.Sc. | INSIGHT London ICC, MRC Clinical Trials Unit at UC, London, UK |
| Gregory | Adam | M.A. | INSIGHT London ICC, MRC Clinical Trials Unit at UC, London, UK |
| Goodall | Katharine | B.A. | INSIGHT London ICC, MRC Clinical Trials Unit at UC, London, UK |
| Harris | Nicola |  | INSIGHT London ICC, MRC Clinical Trials Unit at UC, London, UK |
| Wyncoll | James | B.Sc. | INSIGHT London ICC, MRC Clinical Trials Unit at UC, London, UK |
| Bhagani | S. | M.D. | United Kingdom SCC: Royal Free Hospital |
| Rodger | A. | Ph.D. | United Kingdom SCC: Royal Free Hospital |
| Luntiel | A. | M.D. | United Kingdom SCC: Royal Free Hospital |
| Patterson | C. | M.D. | United Kingdom SCC: Royal Free Hospital |
| Morales | J. | B.Sc | United Kingdom SCC: Royal Free Hospital |
| Witele | E. | B.Sc. | United Kingdom SCC: Royal Free Hospital |
| Preston | A-M | B.Sc. | United Kingdom SCC: Royal Free Hospital |
| Nandani | A. | M.Pharm. | United Kingdom SCC: Royal Free Hospital |
| Price | D.A. | M.D. | Royal Victoria Infirmary |
| Hanrath | Aiden | M.B.B.S. | Royal Victoria Infirmary |
| Nell | Jeremy | M.D. | Royal Victoria Infirmary |
| Patel | Bijal | M.Sc. | Royal Victoria Infirmary |
| Hays | Carole | A.D.N.S. | Royal Victoria Infirmary |
| Jones | Geraldine | B.Sc. | Royal Victoria Infirmary |
| Davidson | Jade | B.T.E.C. Pharm | Royal Victoria Infirmary |
| Goodman | Anna L. | F.R.C.P., D.Phil. | Guy’s & St. Thomas’ NHS Foundation Trust |
| Bawa | T. | M.B.B.S. | Guy’s & St. Thomas’ NHS Foundation Trust |
| Mathews | M. | M.Sc., B.Pharm. | Guy’s & St. Thomas’ NHS Foundation Trust |
| Mazzella | A. | M.R.C.P., M.Sc. | Guy’s & St. Thomas’ NHS Foundation Trust |
| Bisnauthsing | K. | B.Sc. | Guy’s & St. Thomas’ NHS Foundation Trust |
| Aguilar-Jimenez | L. | B.Sc. | Guy’s & St. Thomas’ NHS Foundation Trust |
| Borchini | F. | B.Sc. | Guy’s & St. Thomas’ NHS Foundation Trust |
| Hammett | S. | B.Sc. | Guy’s & St. Thomas’ NHS Foundation Trust |
| Touloumi | Giota | Ph.D. | Greece SCC, National & Kapodistrian University of Athens Medical School |
| Pantazis | Nikos | Ph.D. | Greece SCC, National & Kapodistrian University of Athens Medical School |
| Gioukari | Vicky | B.Sc. | Greece SCC, National & Kapodistrian University of Athens Medical School |
| Souliou | Tania |  | Greece SCC, National & Kapodistrian University of Athens Medical School |
| Antoniadou | A. | M.D | Attikon University General Hospital |
| Protopapas | K. | M.D. | Attikon University General Hospital |
| Kavatha | D. | M.D. | Attikon University General Hospital |
| Grigoropoulou | S. | M.D. | Attikon University General Hospital |
| Tziolos | R-N. | M.D. | Attikon University General Hospital |
| Oikonomopoulo | C. |  | Attikon University General Hospital |
| Moschopoulos | C. | M.D. | Attikon University General Hospital |
| Koulouris | N.G. | M.D. | 1st Respiratory Medicine Department, Athens University Medical School |
| Tzimopoulos | K. | M.D. | 1st Respiratory Medicine Department, Athens University Medical School |
| Koromilias | A. | M.D. | 1st Respiratory Medicine Department, Athens University Medical School |
| Argyraki | K. | M.D. | 1st Respiratory Medicine Department, Athens University Medical School |
| Lourida | P. | M.D. | 1st Respiratory Medicine Department, Athens University Medical School |
| Bakakos | P. | M.D. | 1st Respiratory Medicine Department, Athens University Medical School |
| Kalomenidis | I. | M.D. | Department of Critical Care and Pulmonary Medicine, Evangelismos General Hospital |
| Vlachakos | V. | M.D. | Department of Critical Care and Pulmonary Medicine, Evangelismos General Hospital |
| Barmparessou | Z. | M.D. | Department of Critical Care and Pulmonary Medicine, Evangelismos General Hospital |
| Balis | E. | M.D., Ph.D. | Department of Critical Care and Pulmonary Medicine, Evangelismos General Hospital |
| Zakynthinos | S. | M.D. | Department of Critical Care and Pulmonary Medicine, Evangelismos General Hospital |
| Sigala | I. | M.D. | Department of Critical Care and Pulmonary Medicine, Evangelismos General Hospital |
| Gianniou | N. | M.D. | Department of Critical Care and Pulmonary Medicine, Evangelismos General Hospital |
| Dima | E. | M.D. | Department of Critical Care and Pulmonary Medicine, Evangelismos General Hospital |
| Magkouta | S. | M.D. | Department of Critical Care and Pulmonary Medicine, Evangelismos General Hospital |
| Synolaki | E. | M.D. | Department of Critical Care and Pulmonary Medicine, Evangelismos General Hospital |
| Konstanta | S. | M.D. | Department of Critical Care and Pulmonary Medicine, Evangelismos General Hospital |
| Vlachou | M. | M.Sc., Ph.D. | Department of Critical Care and Pulmonary Medicine, Evangelismos General Hospital |
| Stathopoulou | P. | M.Sc. | Department of Critical Care and Pulmonary Medicine, Evangelismos General Hospital |
| Panagopoulos | P. | MD | Democritus University of Thrace |
| Petrakis | V. | MD | Democritus University of Thrace |
| Papazoglou | D. | MD | Democritus University of Thrace |
| Tompaidou | E. | M.Sc. | Democritus University of Thrace |
| Isaakidou | E. |  | Democritus University of Thrace |
| Poulakou | G. | M.D. | 3rd Department of Medicine, Medical School, NKUA |
| Rapti | V. | M.D. | 3rd Department of Medicine, Medical School, NKUA |
| Leontis | K. | M.D. | 3rd Department of Medicine, Medical School, NKUA |
| Nitsotolis | T. | M.D. | 3rd Department of Medicine, Medical School, NKUA |
| Athanasiou | K. | M.D. | 3rd Department of Medicine, Medical School, NKUA |
| Syrigos | K. | M.D. | 3rd Department of Medicine, Medical School, NKUA |
| Argyraki | K. | M.D. | 3rd Department of Medicine, Medical School, NKUA |
| Myrodia | M-D. | M.D. | 3rd Department of Medicine, Medical School, NKUA |
| Kyriakoulis | K. | M.D. | 3rd Department of Medicine, Medical School, NKUA |
| Trontzas | I. | M.D. | 3rd Department of Medicine, Medical School, NKUA |
| Arfara-Melanini | M. | M.D. | 3rd Department of Medicine, Medical School, NKUA |
| Kolonis | V. | M.D. | 3rd Department of Medicine, Medical School, NKUA |
| Kityo | Cissy | M.D. | Uganda SCC, JCRC/MRC/UVRI Uganda Research Unit |
| Mugerwa | Henry | M.D. | Uganda SCC, JCRC/MRC/UVRI Uganda Research Unit |
| Kiweewa | Francis | M.D., M.P.H. | Uganda SCC, JCRC/MRC/UVRI Uganda Research Unit |
| Kimuli | Ivan | M.D. | Uganda SCC, JCRC/MRC/UVRI Uganda Research Unit |
| Lukaakome | Joseph | M.D. | MRC/UVRI & LSHTM Uganda Research Unit |
| Nsereko | Christoher | M.D. | MRC/UVRI & LSHTM Uganda Research Unit |
| Lubega | Gloria | M.D. | MRC/UVRI & LSHTM Uganda Research Unit |
| Kibirige | Moses | M.D. | MRC/UVRI & LSHTM Uganda Research Unit |
| Nakahima | William |  | MRC/UVRI & LSHTM Uganda Research Unit |
| Wangi | Deus |  | MRC/UVRI & LSHTM Uganda Research Unit |
| Aguti | Evelyne | M.D. | MRC/UVRI & LSHTM Uganda Research Unit |
| Generous | Lilian |  | MRC/UVRI & LSHTM Uganda Research Unit |
| Massa | Rosemary |  | MRC/UVRI & LSHTM Uganda Research Unit |
| Nalaki | Margaret |  | MRC/UVRI & LSHTM Uganda Research Unit |
| Magala | Felix | M.D. | MRC/UVRI & LSHTM Uganda Research Unit |
| Nabaggala | Phiona Kaweesi |  | MRC/UVRI & LSHTM Uganda Research Unit |
| Kidega | Robert | M.D. | Gulu Regional Referral Hospital |
| Kityo | Cissy | M.D., Ph.D. | Gulu Regional Referral Hospital |
| Mugerwa | Henry | M.D. | Gulu Regional Referral Hospital |
| Faith | Oryem Daizy | R.N. | Gulu Regional Referral Hospital |
| Florence | Apio | R.N. | Gulu Regional Referral Hospital |
| Emmanuel | Ocung | B.B.L.T. | Gulu Regional Referral Hospital |
| Beacham | Mugoonyi Paul | M.D. | Gulu Regional Referral Hospital |
| Geoffrey | Amone | B.Sc. | Gulu Regional Referral Hospital |
| Nakiboneka | Dridah | B.Stat. | Gulu Regional Referral Hospital |
| Apiyo | Paska | M.D. | Gulu Regional Referral Hospital |
| Kiweewa | Francis | M.D., M.P.H. | Makerere University Lung Institute |
| Kirenga | Bruce | MBChB, NMED Ph.D. | Makerere University Lung Institute |
| Kimuli | Ivan | MBChB, NMED, M.P.H. | Makerere University Lung Institute |
| Atukunda | Angella | MBChB, NMED | Makerere University Lung Institute |
| Muttamba | Winters | MBChB, NMED | Makerere University Lung Institute |
| Remmy | Kyeyume | B.MLT. | Makerere University Lung Institute |
| Segawa | Ivan | B.Pharm. | Makerere University Lung Institute |
| Pheona | Nsubuga | B.Pharm., M.P.H. | Makerere University Lung Institute |
| Kigere | David | D.N. | Makerere University Lung Institute |
| Mbabazi | Queen Lailah | MBChB | Makerere University Lung Institute |
| Boersalino | Ledra | MBChB | Makerere University Lung Institute |
| Nyakoolo | Grace | B.Sc. | Makerere University Lung Institute |
| Kiweewa | Francis | MD., M.P.H. | Lira Regional Referral Hospital |
| Fred | Aniongo | B.MLT. | Lira Regional Referral Hospital |
| Alupo | Alice | R.N. | Lira Regional Referral Hospital |
| Ebong | Doryn | B.S.N. | Lira Regional Referral Hospital |
| Monday | Edson | B.S.N. | Lira Regional Referral Hospital |
| Nalubwama | Ritah Norah | M.D. | Lira Regional Referral Hospital |
| Kainja | Milton | M.D. | Lira Regional Referral Hospital |
| Ambrose | Munu | D.M.L.T. | Lira Regional Referral Hospital |
| Kwehayo | Vanon | R.N. | Lira Regional Referral Hospital |
| Nalubega | Mary Grace | R.N. | Lira Regional Referral Hospital |
| Ongoli | Augustine | MBChB | Lira Regional Referral Hospital |
| Obbo | Stephen | MBChB, NMED, M.P.H. | Lira Regional Referral Hospital |
| Sebudde | Nicholus | MBChB | Lira Regional Referral Hospital |
| Alaba | Jeniffer |  | Lira Regional Referral Hospital |
| Magombe | Geoffrey | B.Pharm. | Lira Regional Referral Hospital |
| Tino | Harriet | B.Pharm., M.Sc.,P.H.S.M. | Lira Regional Referral Hospital |
| Obonya, E.E. | Emmanuel |  | Lira Regional Referral Hospital |
| Lutaakome | Joseph | M.D. | Masaka Regional Referral Hospital |
| Kitonsa | Jonathan | M.D. | Masaka Regional Referral Hospital |
| Onyango | Martin | M.D. | Masaka Regional Referral Hospital |
| Naboth | Tukamwesiga | M.D. | Masaka Regional Referral Hospital |
| Naluyinda | Hadijah |  | Masaka Regional Referral Hospital |
| Nanyunja | Regina |  | Masaka Regional Referral Hospital |
| Irene | Muttiibwa |  | Masaka Regional Referral Hospital |
| Jane | Biira |  | Masaka Regional Referral Hospital |
| Wimfred | Kyobejja |  | Masaka Regional Referral Hospital |
| Leonard | Ssemazzi |  | Masaka Regional Referral Hospital |
| Deus | Tkiinomuhisha |  | Masaka Regional Referral Hospital |
| Babra | Namasaba |  | Masaka Regional Referral Hospital |
| Taire | Paul |  | Masaka Regional Referral Hospital |
| Lutaakome | Joseph | M.D. | St. Francis Hospital, Nsambya |
| Nabankema | Evelyn | M.D. | St. Francis Hospital, Nsambya |
| Ogavu | Joseph | M.D. | St. Francis Hospital, Nsambya |
| Mugerwa | Oscar | M.D. | St. Francis Hospital, Nsambya |
| Okoth | Ivan | M.D. | St. Francis Hospital, Nsambya |
| Mwebaze | Raymond | M.D. | St. Francis Hospital, Nsambya |
| Mugabi | Timothy | M.D. | St. Francis Hospital, Nsambya |
| Makhoba | Anthony | M.D. | St. Francis Hospital, Nsambya |
| Arikiriza | Phiona |  | St. Francis Hospital, Nsambya |
| Theresa | Nabuuma |  | St. Francis Hospital, Nsambya |
| Nakayima | Hope |  | St. Francis Hospital, Nsambya |
| Frank | Kisuule |  | St. Francis Hospital, Nsambya |
| Ramgi | Patrícia | M.D. | CISPOC:  Centro de Investigaçäo e Treino em Saúde da Polana Caniço, Maputo, Mozambique |
| Pereira | Kássia | M.D., and all site team | CISPOC:  Centro de Investigaçäo e Treino em Saúde da Polana Caniço, Maputo, Mozambique |
| Osinusi | Anu | M.D., M.P.H. | Gilead Sciences, Foster City, CA, USA |
| Cao | Huyen | M.D. | Gilead Sciences, Foster City, CA, USA |
| Klekotka | Paul | M.D., Ph.D. | Eli Lilly and Company, Indianapolis, IN |
| Price | Karen | Ph.D. | Eli Lilly and Company, Indianapolis, IN |
| Nirula | Ajay | M.D., Ph.D. | Eli Lilly and Company, Indianapolis, IN |
| Osei | Suzette | M.D., Ph.D. | Vir Biotechnology / GlaxoSmithKline |
| Tipple | Craig | M.B.B.S., M.R.C.P., Ph.D. | Vir Biotechnology / GlaxoSmithKline |
| Wills | Angela | R.N., M.S.N., M.B.A. | Vir Biotechnology / GlaxoSmithKline |
| Peppercorn | Amanda | M.D. | Vir Biotechnology / GlaxoSmithKline |
| Watson | Helen | B.Sc., M.Sc. | Vir Biotechnology / GlaxoSmithKline |
| Gupta | Rajesh | M.D.,M.S., M.P.H. | Vir Biotechnology / GlaxoSmithKline |
| Alexander | Elizabeth | M.D., M.Sc., F.I.D.S.A. | Vir Biotechnology / GlaxoSmithKline |
| Mogalian | Erik | Pharm.D., Ph.D. | Vir Biotechnology / GlaxoSmithKline |
| Lin | Leo | M.D. | Vir Biotechnology / GlaxoSmithKline |
| Ding | Xiao | Ph.D. | Vir Biotechnology / GlaxoSmithKline |
| Margolis | David | M.D., M.P.H. | Brii Biosciences |
| Yan | Li | M.D., Ph.D. | Brii Biosciences |
| Girardet | Jean-Luc | Ph.D. | Brii Biosciences |
| Ma | Ji | Ph.D. | Brii Biosciences |
| Hong | Zhi | Ph.D. | Brii Biosciences |
| Zhu | Quing | Ph.D. | Brii Biosciences |
| Seegobin | Seth | PhD | AstraZeneca |
| Gibbs | Michael | PhD | AstraZeneca |
| Latchman | Mickel | BSc | AstraZeneca |
| Hasior | Katarzyna | MSc | AstraZeneca |
| Bouquet | Jerome | PhD | AstraZeneca |
| Wei | Jianxin | PhD | AstraZeneca |
| Streicher | Katie | PhD | AstraZeneca |
| Schmelzer | Albert | PhD | AstraZeneca |
| Brooks | Dennis | MD, PhD | AstraZeneca |
| Butcher | Jonny | BSc | AstraZeneca |
| Tonev | Dimitar | MD | AstraZeneca |
| Arbetter | Douglas | MPH | AstraZeneca |
| Damstetter | Philippe | MSc | AstraZeneca |
| Legenne | Philippe | M.D | Molecular Partners and Novartis |
| Stumpp | Michael | PhD | Molecular Partners and Novartis |
| Goncalves | Susana | Pharm. D | Molecular Partners and Novartis |
| Ramanathan | Krishnan | Ph.D | Molecular Partners and Novartis |
| Chandra | Richa | M.D. | Molecular Partners and Novartis |
| Baseler | Beth | M.S. | Leidos Biomedical Research, Inc., Frederick, MD, USA |
| Teitelbaum | Marc | M.D. | Leidos Biomedical Research, Inc., Frederick, MD, USA |
| Schechner | Adam | M.D. | Leidos Biomedical Research, Inc., Frederick, MD, USA |
| Holley | H. Preston | M.D. | Leidos Biomedical Research, Inc., Frederick, MD, USA |
| Jankelevich | Shirley | M.D. | Leidos Biomedical Research, Inc., Frederick, MD, USA |
| Adams | Amy | M.S. | Leidos Biomedical Research, Inc., Frederick, MD, USA |
| Becker | Nancy | B.S.N. | Leidos Biomedical Research, Inc., Frederick, MD, USA |
| Dolney | Suzanne | B.S.N. | Leidos Biomedical Research, Inc., Frederick, MD, USA |
| Hissey | Debbie |  | Leidos Biomedical Research, Inc., Frederick, MD, USA |
| Simpson | Shelly | M.S. | Leidos Biomedical Research, Inc., Frederick, MD, USA |
| Kim | Mi Ha | Ph.D. | Leidos Biomedical Research, Inc., Frederick, MD, USA |
| Beeler | Joy | M.P.H. | Leidos Biomedical Research, Inc., Frederick, MD, USA |
| Harmon | Liam | B.A. | Leidos Biomedical Research, Inc., Frederick, MD, USA |
| Asomah | Mabel | M.S.H.S | Leidos Biomedical Research, Inc., Frederick, MD, USA |
| Jato | Yvonne | M.P.H. | Leidos Biomedical Research, Inc., Frederick, MD, USA |
| Stottlemyer | April | A.A. | Leidos Biomedical Research, Inc., Frederick, MD, USA |
| Tang | Olivia | B.S. | Leidos Biomedical Research, Inc., Frederick, MD, USA |
| Vanderpuye | Sharon | B.A. | Leidos Biomedical Research, Inc., Frederick, MD, USA |
| Yeon | Lindsey | B.S. | Leidos Biomedical Research, Inc., Frederick, MD, USA |
| Buehn | Molly | M.S. | Leidos Biomedical Research, Inc., Frederick, MD, USA |
| Eccard-Koons | Vanessa | M.S. | Leidos Biomedical Research, Inc., Frederick, MD, USA |
| Frary | Sadie | M.S. | Leidos Biomedical Research, Inc., Frederick, MD, USA |
| MacDonald | Leah | M.S. | Leidos Biomedical Research, Inc., Frederick, MD, USA |
| Cash | Jennifer | B.S. | Leidos Biomedical Research, Inc., Frederick, MD, USA |
| Hoopengardner | Lisa | M.S. | Leidos Biomedical Research, Inc., Frederick, MD, USA |
| Linton | Jessica | M.S. | Leidos Biomedical Research, Inc., Frederick, MD, USA |
| Schaffhauser | Marylu | B.A. | Leidos Biomedical Research, Inc., Frederick, MD, USA |
| Nelson | Michaela | B.S. | Leidos Biomedical Research, Inc., Frederick, MD, USA |
| Spinelli-Nadzam | Mary | B.S. | Leidos Biomedical Research, Inc., Frederick, MD, USA |
| Proffitt | Calvin | M.A. | Leidos Biomedical Research, Inc., Frederick, MD, USA |
| Lee | Christopher | B.S. | Leidos Biomedical Research, Inc., Frederick, MD, USA |
| Engel | Theresa | M.F.S. | Leidos Biomedical Research, Inc., Frederick, MD, USA |
| Fontaine | Laura | B.S.N. | Leidos Biomedical Research, Inc., Frederick, MD, USA |
| Osborne | C.K. | B.S. | Leidos Biomedical Research, Inc., Frederick, MD, USA |
| Hohn | Matt | M.B.A. | Leidos Biomedical Research, Inc., Frederick, MD, USA |
| Galcik | Michael | M.S. | Leidos Biomedical Research, Inc., Frederick, MD, USA |
| Thompson, | DeeDee | A.A. | Leidos Biomedical Research, Inc., Frederick, MD, USA |
| Kopka | Stacey | M.S. | Leidos Biomedical Research, Inc., Frederick, MD, USA |
| Shelley | Denise M. | M.S. | Leidos Biomedical Research, Inc., Frederick, MD, USA |
| Mendez | Gregg | Ph.D. | Frederick National Laboratory for Cancer Research/Leidos Biomedical Research, Inc., Frederick, MD. |
| Brown | Shawn | M.S. | Frederick National Laboratory for Cancer Research/Leidos Biomedical Research, Inc., Frederick, MD. |
| Albert | Sara | M.P.H. | Leidos Biomedical Research, Inc., Frederick, MD, USA |
| Balde | Abby | M.P.H. | Leidos Biomedical Research, Inc., Frederick, MD, USA |
| Baracz | Michelle | M.S. | Leidos Biomedical Research, Inc., Frederick, MD, USA |
| Bielica | Mona | M.Ed | Leidos Biomedical Research, Inc., Frederick, MD, USA |
| Billouin-Frazier | Shere | M.Sc. | Leidos Biomedical Research, Inc., Frederick, MD, USA |
| Choudary | Jay | M.B.A. | Leidos Biomedical Research, Inc., Frederick, MD, USA |
| Dixon | Mary | A.A. | Leidos Biomedical Research, Inc., Frederick, MD, USA |
| Eyler | Carolyn |  | Leidos Biomedical Research, Inc., Frederick, MD, USA |
| Frye | Leanne | M.A. | Leidos Biomedical Research, Inc., Frederick, MD, USA |
| Gertz | Jensen | M.B.A. | Leidos Biomedical Research, Inc., Frederick, MD, USA |
| Giebeig | Lisa | M.S. | Leidos Biomedical Research, Inc., Frederick, MD, USA |
| Gulati | Neelam | B.S. | Leidos Biomedical Research, Inc., Frederick, MD, USA |
| Hankinson | Liz | B.S. | Leidos Biomedical Research, Inc., Frederick, MD, USA |
| Hogarty | Debi |  | Leidos Biomedical Research, Inc., Frederick, MD, USA |
| Huber | Lynda |  | Leidos Biomedical Research, Inc., Frederick, MD, USA |
| Krauss | Gary | B.S. | Leidos Biomedical Research, Inc., Frederick, MD, USA |
| Lake | Eileen |  | Leidos Biomedical Research, Inc., Frederick, MD, USA |
| Manandhar | Meryan | M.P.H. | Leidos Biomedical Research, Inc., Frederick, MD, USA |
| Rudzinski | Erin | B.S. | Leidos Biomedical Research, Inc., Frederick, MD, USA |
| Sandrus | Jen | A.A. | Leidos Biomedical Research, Inc., Frederick, MD, USA |
| Suders | Connie | M.B.A. | Leidos Biomedical Research, Inc., Frederick, MD, USA |
| Natarajan | Ven | Ph.D. | Frederick National Laboratory for Cancer Research/Leidos Biomedical Research, Inc., Frederick, MD. |
| Rupert | Adam W. | B.S., MT(ASCP) | Frederick National Laboratory for Cancer Research/Leidos Biomedical Research, Inc., Frederick, MD. |
| Baseler | Michael | Ph.D. | Frederick National Laboratory for Cancer Research/Leidos Biomedical Research, Inc., Frederick, MD. |
| Lynam | Danielle | M.S. | Frederick National Laboratory for Cancer Research/Leidos Biomedical Research, Inc., Frederick, MD. |
| Imamichi | Tom | Ph.D. | Frederick National Laboratory for Cancer Research/Leidos Biomedical Research, Inc., Frederick, MD. |
| Laverdure | Sylvain | Ph.D. | Frederick National Laboratory for Cancer Research/Leidos Biomedical Research, Inc., Frederick, MD. |
| McCormack | Ashley | M.P.S. | Frederick National Laboratory for Cancer Research/Leidos Biomedical Research, Inc., Frederick, MD. |
| Paudel | Sharada | Ph.D. | Frederick National Laboratory for Cancer Research/Leidos Biomedical Research, Inc., Frederick, MD. |
| Cook | Kyndal | B.S. | Frederick National Laboratory for Cancer Research/Leidos Biomedical Research, Inc., Frederick, MD. |
| Haupt | Kendra | B.S. | Frederick National Laboratory for Cancer Research/Leidos Biomedical Research, Inc., Frederick, MD. |
| Khan | Ayub | Ph.D. | Frederick National Laboratory for Cancer Research/Leidos Biomedical Research, Inc., Frederick, MD. |
| Hazen | Allison | M.S. | Frederick National Laboratory for Cancer Research/Leidos Biomedical Research, Inc., Frederick, MD. |
| Badralmaa | Yunden | M.S. | Frederick National Laboratory for Cancer Research/Leidos Biomedical Research, Inc., Frederick, MD. |
| Smith | Kenneth |  | Advanced Biomedical Laboratories, LLC., Cinnaminson, NJ, USA |
| Patel | Bhakti |  | Advanced Biomedical Laboratories, LLC., Cinnaminson, NJ, USA |
| Kubernac | Amanda |  | Advanced Biomedical Laboratories, LLC., Cinnaminson, NJ, USA |
| Kubernac | Robert |  | Advanced Biomedical Laboratories, LLC., Cinnaminson, NJ, USA |
| Hoover | Marie L. | Ph.D. | Advanced Biomedical Laboratories, LLC., Cinnaminson, NJ, USA |
| Solomon | Courtney |  | Advanced Biomedical Laboratories, LLC., Cinnaminson, NJ, USA |
| Rashid | Marium |  | Advanced Biomedical Laboratories, LLC., Cinnaminson, NJ, USA |
| Murphy | Joseph |  | Advanced Biomedical Laboratories, LLC., Cinnaminson, NJ, USA |
| Brown | Craig |  | PCI Pharma Services |
| DuChateau | Nadine |  | PCI Pharma Services |
| Ellis | Sadie |  | PCI Pharma Services |
| Flosi | Adam |  | PCI Pharma Services |
| Fox | Lisa |  | PCI Pharma Services |
| Johnson | Les |  | PCI Pharma Services |
| Nelson | Rich |  | PCI Pharma Services |
| Stojanovic | Jelena |  | PCI Pharma Services |
| Treagus | Amy |  | PCI Pharma Services |
| Wenner | Christine |  | PCI Pharma Services |
| Williams | Richard |  | PCI Pharma Services |
